# Molecular Mechanism of Disease-Associated Mutations in the Pre-M1 Helix of NMDA Receptors and Potential Rescue Pharmacology

**DOI:** 10.1371/journal.pgen.1006536

**Published:** 2017-01-17

**Authors:** Kevin K. Ogden, Wenjuan Chen, Sharon A. Swanger, Miranda J. McDaniel, Linlin Z. Fan, Chun Hu, Anel Tankovic, Hirofumi Kusumoto, Gabrielle J. Kosobucki, Anthony J. Schulien, Zhuocheng Su, Joseph Pecha, Subhrajit Bhattacharya, Slavé Petrovski, Adam E. Cohen, Elias Aizenman, Stephen F. Traynelis, Hongjie Yuan

**Affiliations:** 1 Department of Pharmacology, Emory University School of Medicine, Atlanta, Georgia, United States of America; 2 Department of Neurology, Xiangya Hospital, Central South University, Changsha, China; 3 Department of Chemistry and Chemical Biology, Howard Hughes Medical Institute, Harvard University, Cambridge, Massachusetts, United States of America; 4 Department of Neurobiology, University of Pittsburgh School of Medicine and Pittsburgh Institute for Neurodegenerative Diseases, Pittsburgh, Pennsylvania, United States of America; 5 Department of Medicine, The University of Melbourne, Austin Health and Royal Melbourne Hospital, Melbourne, Victoria, Australia; 6 Center for Functional Evaluation of Rare Variants (CFERV), Emory University School of Medicine, Rollins Research Center, Atlanta, Georgia, United States of America; Florey Institute of Neuroscience and Mental Health, AUSTRALIA

## Abstract

N-methyl-D-aspartate receptors (NMDARs), ligand-gated ionotropic glutamate receptors, play key roles in normal brain development and various neurological disorders. Here we use standing variation data from the human population to assess which protein domains within NMDAR GluN1, GluN2A and GluN2B subunits show the strongest signal for being depleted of missense variants. We find that this includes the GluN2 pre-M1 helix and linker between the agonist-binding domain (ABD) and first transmembrane domain (M1). We then evaluate the functional changes of multiple missense mutations in the NMDAR pre-M1 helix found in children with epilepsy and developmental delay. We find mutant GluN1/GluN2A receptors exhibit prolonged glutamate response time course for channels containing 1 or 2 GluN2A-P552R subunits, and a slow rise time only for receptors with 2 mutant subunits, suggesting rearrangement of one GluN2A pre-M1 helix is sufficient for rapid activation. GluN2A-P552R and analogous mutations in other GluN subunits increased the agonist potency and slowed response time course, suggesting a functionally conserved role for this residue. Although there is no detectable change in surface expression or open probability for GluN2A-P552R, the prolonged response time course for receptors that contained GluN2A-P552R increased charge transfer for synaptic-like activation, which should promote excitotoxic damage. Transfection of cultured neurons with GluN2A-P552R prolonged EPSPs, and triggered pronounced dendritic swelling in addition to excitotoxicity, which were both attenuated by memantine. These data implicate the pre-M1 region in gating, provide insight into how different subunits contribute to gating, and suggest that mutations in the pre-M1 helix can compromise neuronal health. Evaluation of FDA-approved NMDAR inhibitors on the mutant NMDAR-mediated current response and neuronal damage provides a potential clinical path to treat individuals harboring similar mutations in NMDARs.

## Author Summary

The increased use of next-generation sequencing for neurological patients has led to a growing catalog of patient-ascertained variants in N-methyl-D-aspartate receptor (NMDAR) subunits, which play important roles in normal brain development and have been implicated in epilepsy, language disorders, motor disorders, learning disorders, autism, attention deficit hyperactivity disorder, developmental delay, and schizophrenia. Studies that provide functional analysis of the mutant proteins produced by missense mutations are lacking. Here, we use the largest currently available sample of human standing variation to illustrate the landscape of missense intolerance within the GluN1, GluN2A and GluN2B subunits, and provide the first evaluation of the molecular mechanisms of mutations in NMDAR pre-M1 helix that links the agonist binding domain to the channel pore in patients with epilepsy and/or intellectual disability. This region of the subunit is depleted of missense variants in the healthy population, which from the population genetics view, is consistent with what we would expect if mutations in these regions were associated with severe disorders. Our functional results suggest that mutations in this region of the receptor have profound effects on receptor and neuronal function, which may contribute to patient symptoms and could contribute to neuronal damage. This finding further suggests that evaluation of strategies to treat patients with similar mutations in NMDAR that are neurotoxic may preserve grey matter.

## Introduction

Recent analysis of whole exome data has shown that genes encoding excitatory post synaptic receptors, including the *GRIN* family, are some of the least tolerant genes in the body [1]. They show significantly less non-synonymous variation than expected in specific regions [2], and harbor a large number of disease-associated *de novo* mutations ([3] [4]). To better understand the previously reported genic intolerance, here we illustrate the distribution of missense depletion within the relevant *GRIN* genes to highlight sub-regions within these *GRIN* genes that appear to have been under the strongest purifying selection in the human population. We then further focus on a series of *GRIN* patient-ascertained missense mutations that reside among some of the least tolerant components of the NMDA receptor (NMDAR), which mediates a slow Ca^2+^-permeable component of excitatory postsynaptic signaling in the central nervous system following release of glutamate into the synaptic cleft.

**Fig 1:**
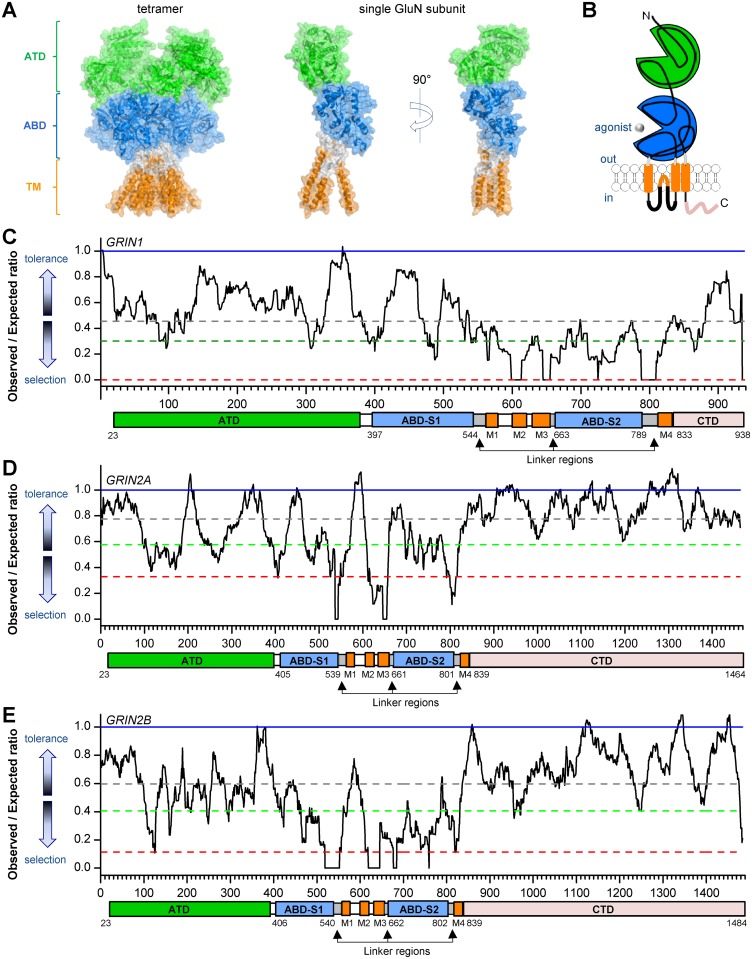
Intolerance analysis of genetic variation across functional domains of GluN1, GluN2A and GluN2B. **A**, Ribbon structure of homology model of a tetrameric NMDAR and a single GluN subunit [10,12]. **B**, A cartoon illustrating the domain arrangement of an individual GluN subunit. ATD—amino terminal domain (in GREEN), S1 and S2 –first and second polypeptide sequences comprising the agonist binding domain (ABD, in BLUE), linker regions (S1-M1 linker, M3-S2 linker, and S2-M4 linker; in GRAY), M1, M3, and M4 –transmembrane domains (TM, in ORANGE), M2 –re-entrant pore loop (in ORANGE), and CTD—carboxy-terminal domain (in PINK). **C**, *GRIN1*; **D**, *GRIN2A*; **E**, *GRIN2B*: Sliding window OE-ratio estimates (black full line), Neutrality expected OE-ratio estimate (blue full line), Median OE-ratio for the gene (dark grey dashed line), 25^th^ percentile of OE-ratio (green dashed line), 5^th^ percentile of OE-ratio (red dashed line).

NMDARs are tetrameric complexes of subunits, each of which contains four semiautonomous domains: the amino-terminal domain (ATD), the agonist-binding domain (ABD), the transmembrane domain (TMD), and a cytosolic carboxyl terminal domain (CTD) [5]. The ABDs of all glutamate receptor ion channels fold into a bi-lobed clamshell-shaped structure (Fig 1A and 1B), with an upper and lower lobe referred to as D1 and D2, respectively. Crystal structures of isolated ABDs of glutamate receptor ion channels revealed that upon agonist binding, atomic contacts between the agonist and the D1 and D2 lobes promote a closed-cleft conformation of the ABD, which is translated into a rearrangement of short linkers connected to the transmembrane helices [6–15]. For AMPA receptors, the degree of cleft closure correlates with activation of the receptor [13,16,17], which has been hypothesized to involve translation of the M3 transmembrane helices away from the central axis of the pore, creating a path for ions to traverse the lipid bilayer [18]. However, a functional and structural understanding of the conformational changes that follow agonist binding and coupling of ABD cleft closure to opening of the ion channel pore is lacking. X-ray crystal structures of tetrameric, membrane-spanning AMPAR and NMDAR revealed several novel structural features well-positioned to transduce agonist binding into channel opening [10,12,18]. In particular, the linker between the ABD and first transmembrane helix contains a short helix parallel to the lipid bilayer referred to as the pre-M1 helix.

Recent whole exome sequencing studies of patients with idiopathic neurological and psychiatric disorders have revealed that genes encoding NMDAR subunits are frequently mutated in neurodevelopmental brain disorders [3,19,20]. Indeed, several missense *de novo* mutations have been identified in NMDAR genes within exons encoding the pre-M1 region. For example, two *GRIN2A* mutations leading to GluN2A-A548T and GluN2A-P552R were identified in patients with seizure disorders and intellectual disability [20,21], a *GRIN2B* mutation encoding GluN2B-P553L was found in a patient with severe developmental delay [20], and two *GRIN1* mutations corresponding to GluN1-D552E and GluN1-P557R were identified in patients with intellectual disability and/or epilepsy [22,23]. By contrast, no missense variants exist in the ExAC data set in the region encoding the S1-M1 linker in *GRIN1*, *GRIN2A or GRIN2B* (Exome Aggregation Consortium, Cambridge, MA http://exac.broadinstitute.org, accessed 4-27-2016). The lack of missense variation in the S1-M1 linker coupled with over-representation of disease-associated *de novo* variants is unlikely due to chance (Table 1, S1 Table), consistent with the idea that these non-synonymous variants in this region may be harmful. Although the occurrence of these mutations suggests that the pre-M1 region is critically involved in NMDAR function, no information currently exists about the functional effects of these mutations [3,20–26].

**Table 1:** Analysis of genetic variation on S1-M1 linker in *GRIN1*, *GRIN2A*, *and GRIN2B*.

	S1-M1 linker missense				Total missense		P-value*
	Residues	Synonymous	gnomAD	Disease	gnomAD	Disease (*de novo*)	
***GRIN1***	545–561	5	3	3	166	12	0.0076
***GRIN2A***	540–556	4	0	2	628	13	0.0005
***GRIN2B***	541–557	5	0	1	429	21	0.04667

*Fisher's exact test comparing the rate of missense variants found among the gnomAD cohort to the rate of missense variants found among patient-ascertained pathogenic-reported *de novo* mutations (Supplemental S1 Table), α_corrected_ = 0.016

Since mutations in the pre-M1 region appear to be associated with disease states and the region is well-positioned to control channel gating, we explored the contribution of pre-M1 residues to channel gating and receptor function. We used mutagenesis coupled with whole cell and single channel patch clamp recordings of receptor currents to directly monitor the gating isomerization and infer properties of mutant and wild type (WT) receptors. To further examine the mechanism by which human mutations alter channel function and thereby contribute to human disease, we introduced a disease-causing mutant channel into cultured cortical neurons to assess the effects of the mutation on synaptic transmission and neuronal health. Our results support the hypothesis that the pre-M1 cuff helix is critically involved in the process of channel opening and closing, and suggest that mutations in this region have profound effects on receptor and neuronal function that contribute to patient symptoms. Several of the disease-associated mutations produce a gain-of-function. The resulting increase in transmembrane cation flux caused by GluN2A-P552R transfected into neurons triggered pronounced dendritic blebbing and excitotoxic death *in vitro*. Further, the NMDAR-mediated neuronal damage could be attenuated by the FDA-approved NMDAR inhibitor memantine, providing a potential clinical path forward to treat individuals harboring similar gain of function NMDAR mutations.

## Results

### Regional variation of purifying selection within *GRIN1*, *GRIN2A* and *GRIN2B*

Using standing variation data from the human population to identify genic sub-regions where missense variation is most depleted has been shown to be a useful measure of where clinically relevant variants are likely to reside [27]. Here we screen the protein-coding sequence of *GRIN1*, *GRIN2A* and *GRIN2B* to highlight particular sub-regions of these genes that appear to have been under the strongest purifying selection within these overall intolerant genes. To do this we use a sliding window approach of 31 residues (93 nucleotides) to compare the proportion of observed missense variants from a large human sample of 141,352 unrelated individuals (the gnomAD data set; beta release accessed November 2016; [28]) to the expected proportion of missense variants given the underlying sequence context of the window (Methods and Fig 1C–1E). We refer to this as the sliding window Observed proportion / Expected proportion ratio (OE-ratio). As previously reported [1], given the total observed genetic variation in these genes, it is apparent that all three genes are overall depleted of missense variants. This is illustrated by the drop of the median OE-ratio from the y-axis value of 1, which is the median OE-ratio we would expect to be near if this sequence had not been under purifying selection (Methods and Fig 1C–1E).

**Fig 2:**
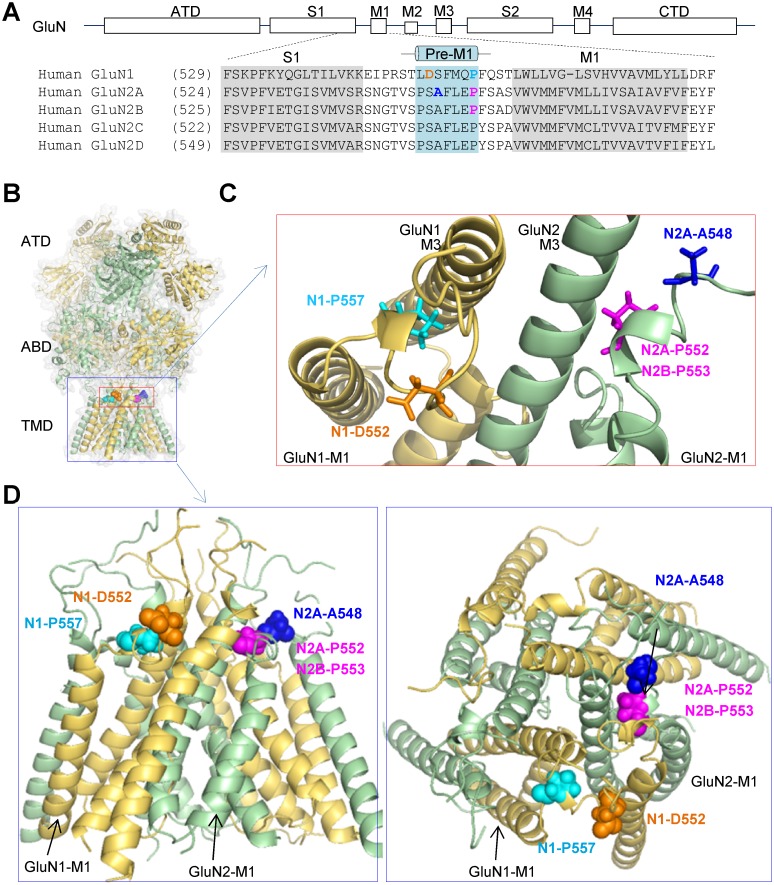
Locations of pre-M1 mutations. **A**, Domain architecture of NMDARs and protein sequence alignment showing pre-M1 helix across NMDAR subunits. **B-D**, Ribbon structure of homology model of GluN1/GluN2A built from GluN1/GluN2B [10,12]. Five residues harboring mutations in human patients are highlighted: GluN1-D552E (in GOLD), GluN1-P557R (in LIGHT BLUE), GluN2A-A548T (in BLUE), GluN2A-P552R (in MAGENTA), and GluN2B-P553L (in MAGENTA). The mutation labels refer to GluN1 as N1 and GluN2 as N2. ATD—amino terminal domain, S1 and S2 –first and second polypeptide sequences comprising the agonist binding domain (ABD), M1, M3, and M4 –transmembrane helices, and M2 –re-entrant pore loop. The mutation information is given in Table 2.

The linker connecting the agonist binding domain and the first transmembrane helix in AMPAR and NMDAR subunits contains a short two-turn helix, referred to as the pre-M1 helix [10,12,18]. This region is conserved across all NMDAR GluN2 subunits [29] (Fig 2A) and is in van der Waals contact with the M3 helix (Fig 2B–2D). The proximity to the D2 domain that moves with agonist binding may allow the pre-M1 region to influence movement and position of the M3 helices that form the channel gate (Fig 2B–2D). Focusing on the sliding windows that make up the lowest OE-ratio estimates (5^th^ percentile) within these genes, we find that these regions overlap with the S1-M1 linker region in *GRIN2A* (with 12 / 19 S1-M1 linker region residues being among the lowest 5% OE-ratio scores) and *GRIN2B* (with 12 / 19 S1-M1 linker region residues being among the lowest 5% OE-ratio scores) (Fig 1D and 1E).

### Pre-M1 mutations in human neurological diseases

**Fig 3:**
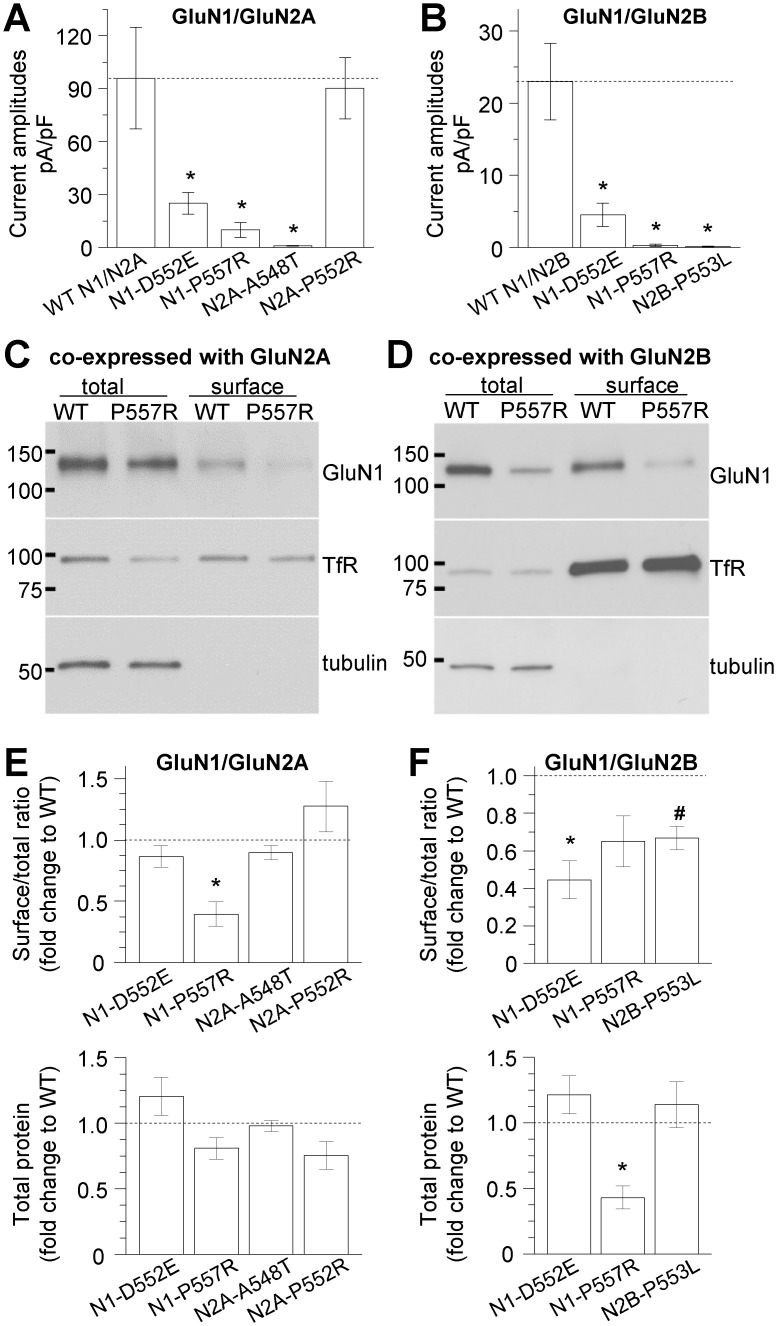
Pre-M1 mutations affect current amplitudes and NMDAR surface expression. **A,B**, Summary of current amplitudes evoked by 1 mM glutamate and 100 μM glycine in whole cell voltage clamp recordings from HEK293 cells expressing human NMDARs (holding at -60 mV). *p < 0.05, see Table 3 and S4 Table for statistical analyses. The graph legends refer to GluN1 as N1 and GluN2 as N2. **C-F**, The surface proteins of HEK293 cells transiently expressing WT or mutated human NMDARs were labeled with biotin and pulled down with avidin-conjugated beads. The total and surface protein fractions were immunoblotted for GluN1, GluN2A or GluN2B, transferrin receptor (TfR), and tubulin. Representative blots are shown for HEK293 cells expressing GluN1/GluN2A and GluN1-P557R/GluN2A (**C**), and GluN1/GluN2B and GluN1-P557R/GluN2B (**D**). Chemiluminescence signals were quantified by densitometry, and the ratio of surface-to-total protein and total protein levels for each mutant were plotted as the fold-change from WT (dashed line). Total protein levels were normalized to tubulin levels. The data were analyzed by one-way ANOVA and paired t-tests between respective mutant and WT controls, with Benjamini-Hochberg correction for multiple comparisons (**E**, GluN1/GluN2A surface/total: F(7,28) = 5.081, p < 0.001; *p = 0.019; GluN1-D552E/GluN2A p = 0.475; 2A-A548T p = 0.453; GluN1/GluN2A-P552R p = 0.404; GluN1/GluN2A total (lower panel): F(7,28) = 2.050, p = 0.084; **F**, GluN1/GluN2B surface/total: F(5,28) = 7.585, p < 0.001; *p = 0.025; ^#^p = 0.018; GluN1-P557R/GluN2B p = 0.102; GluN1/GluN2B total (lower panel): F(5,28) = 4.903, p = 0.002; *p = 0.018; GluN1-D552E/GluN2B p = 0.473; GluN1/GluN2B-P553L p = 0.649). See also S1 Fig.

Multiple *de novo* mutations (GluN1-D552E, GluN1-P557R, GluN2A-A548T, GluN2A-P552R, GluN2B-P553L) (Fig 2B–2D) (Table 2) have been identified in the pre-M1 region in patients with epilepsy and/or developmental delay [20–23], however there is no information available about the effects of these missense mutations on NMDAR function. We therefore evaluated the current response, agonist potency, and cell surface expression of each of these mutations as a step toward understanding the role of this region in channel function. NMDARs containing GluN1-D552E, GluN1-P557R, and GluN2A-A548T showed reduced current responses to 1000 μM glutamate and 100 μM glycine assessed by whole cell voltage clamp current recordings on transfected HEK293 cells (Fig 3A) (Table 3), and differential effects on agonist potency evaluated by two-electrode voltage clamp current recordings on *Xenopus* oocytes (Table 3). The mutation GluN2B-P553L rendered the receptors virtually non-functional (Fig 3B) (Table 3). Surface protein biotinylation and subsequent western blotting of transfected HEK293 cells showed that the ratio of surface-to-total protein expression was clearly reduced for both GluN1-P557R when co-expressed with GluN2A and for GluN1-D552E when co-expressed with GluN2B, whereas the total expression of GluN1-P557R was significantly reduced when co-expressed with GluN2B (Fig 3C–3F and S1 Fig), consistent with the reduced current response amplitudes. However, the expression levels of GluN2A-A548T were unchanged, suggesting that the reduced response amplitude for this mutation is due to a functional change in the receptor. The ratio of surface-to-total protein levels for GluN2B-P553L was reduced to 67 ± 15% of wild type (WT) (Fig 3F and S1 Fig), which suggests that the complete loss of current responses for GluN2B-P553L is not due to a lack of surface expression but rather a functional alteration in the receptor. Whereas NMDARs containing GluN2A-P552R showed surface expression and current response amplitudes similar to WT GluN1/GluN2A receptors (Fig 3 and S1 Fig), they exhibited a highly unusual response time course, which we set out to further investigate. GluN2A-P552R was identified in a patient with delayed psychomotor development, intellectual disability, inability to speak, and epilepsy since 9 months of age [20] (Table 2). In that study, a cerebral CT scan revealed pronounced peripheral liquor spaces mainly in the frontal region, suggesting a reduction in grey matter. However, at the age of 18 patient height and head circumference were normal. The missense mutation was Chr16(GRCh37):g.9928084G>C, which corresponds to c.1655C>G, resulting in the amino acid change Pro552Arg. This mutation, which was absent in the parents, was rated as “probably damaging” with a PolyPhen-2 [30] score of 1.00. The Scale Invariant Feature Transformation (SIFT) [31] also predicted that GluN2A-P552R would affect protein function with a score of 0.00. This GluN2A-P552R variant is not reported in any existing SNP databases (ncbi.nlm.nih.gov/SNP or exac.broadinstitute.org, accessed in 4/2016), suggesting it is not found in healthy individuals and consistent with the idea that mutations at this site contribute to the neurological complications observed in these patients.

**Table 2:** Patients’ and mutations’ information.

	Patient-1	Patient-2	Patient-3	Patient-4	Patient-5
**Mutation**	GluN1-D552E	GluN1-P557R	GluN2A-A548T	GluN2A-P552R	GluN2B-P553L
**Genotype**	c.1656C>G	c.1670C>G	c.1642G>A	c.1655C>G	c.1658C>T
**Gender**	M	M	F	F	M
**Phenotype**	Epilepsy / ID	ID	Epilepsy	Epilepsy / DD	ID
**Seizure Type**	GTCS		LKS		
**Age of Onset**	at birth			9 months	
**Delays**	Severe ID	Severe ID, poor speech, severe hypotonia and feeding disorders in early infancy, developmental delay (walking/speech), behavioral and mood disorders	NA	At 3 months her development stagnated. Severely delayed psychomotor development, didn't develop speech.	Started to smile at one year of age, started to reach at one and a half years of age; didn't develop speech and did not learn to walk.
**Source**	[22]	[23]	[21]	[20]	[20]

DD: developmental delay; ID: intellectual disability; LKS Landau-Kleffner Syndrome, GTCS: Generalized tonic-clonic seizure, NA: not available.

**Table 3:** Summary of pharmacological and functional properties of pre-M1 mutations.

	Amplitude (peak, pA/pF)^Φ^	Glutamate, EC_50_, μM (n)^Σ^	Glycine, EC_50_, μM (n)^Σ^
**GluN1/GluN2A**	96 ± 29 (10)	4.1 ± 0.32 (27)	1.4 ± 0.14 (18)
**GluN1-D552E/GluN2A**	25 ± 6.1 (14)*	17 ± 2.2 (11)*	3.7 ± 0.32 (9)*
**GluN1-P557R/GluN2A**	9.9 ± 4.2 (7)*	0.33 ± 0.01 (7)*	0.18 ± 0.02 (6)*
**GluN1/GluN2A-A548T**	0.87 ± 0.21 (7)*	17 ± 1.3 (10)*	2.4 ± 0.09 (9)*
**GluN1/GluN2B**	23 ± 5.3 (11)	1.5 ± 0.13 (12)	0.39 ± 0.03 (11)
**GluN1-D552E/GluN2B**	4.5 ± 1.6 (8)*	3.3 ± 0.43 (6)*	0.66 ± 0.07 (6)*
**GluN1-P557R/GluN2B**	0.28 ± 0.18 (6)*	0.09 ± 0.01 (6)*	0.07 ± 0.01 (5)*
**GluN1/GluN2B-P553L**	0.12 ± 0.08 (6)*	─^$^	─^$^

Human GluN1 and GluN2 were used for all experiments

^$^ current < 50 nA at 1000 μM glutamate and 100 μM glycine

^Φ^ data were from whole-cell voltage clamp current recordings of transfected HEK293 cells

^Σ^ the data were from two-electrode voltage clamp current recording on *Xenopus* oocytes

* p < 0.05 compared to corresponding WT receptors; one-way ANOVA, Tukey post hoc, see S4 Table for F statistics and p-values.

### GluN2A-P552R pre-M1 mutation enhances agonist potency

**Fig 4:**
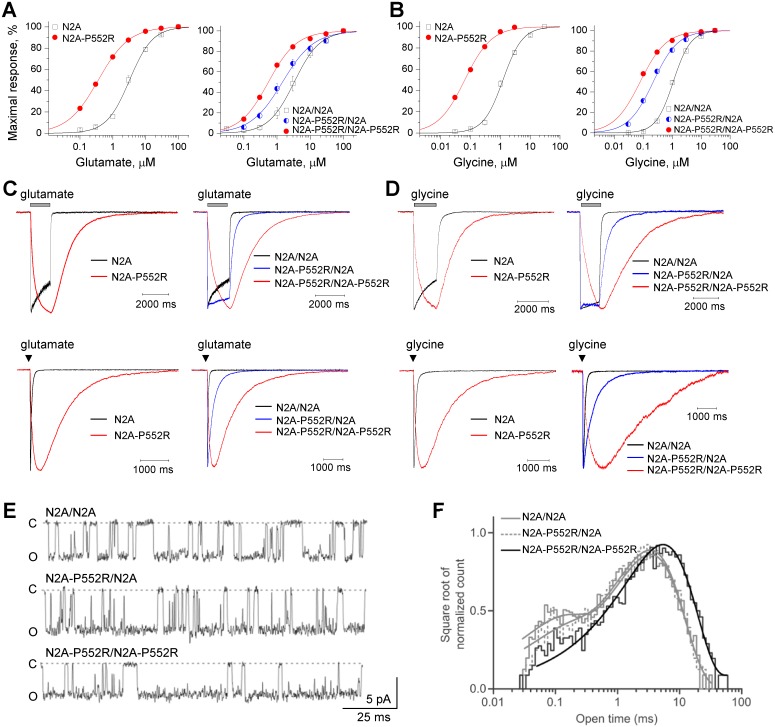
GluN2A-P552R increases agonist potency and alters the NMDAR response time course. **A**, Steady-state concentration-response relationship for glutamate activation of human di-heteromeric (*left panel*) GluN1/GluN2A receptors and rat tri-heteromeric GluN1/GluN2A (*right panel*) containing 0, 1, or 2 copies of GluN2A-P552R receptors (labelled N2A/N2A, N2A/N2A-P552R, N2A-P552/N2A-P552R) expressed in *Xenopus* oocytes with 100 μM glycine present in all solutions. **B**, Concentration-effect relationship for glycine activation of human di-heteromeric (*left panel*) and rat tri-heteromeric (*right panel*) GluN1/GluN2A and GluN1/GluN2A-P552R receptors with 100 μM glutamate present in all solutions. **C**, Whole cell voltage clamp recording of di-heteromeric (*left panels*) and tri-heteromeric (*right panels*) GluN1/GluN2A-P552R receptor responses to rapid application of long (1.5 sec, *upper panels*) and brief (5 ms, *lower panels*) application of 1000 μM glutamate; saturating glycine (30 μM) was present in all of the solutions. Fitted parameters describing the time course of the response to long glutamate application are given in Table 4. The rise time and weighted tau describing the response to brief 5 ms application of glutamate were 7 ± 0.7 and 42 ± 4.3 ms for WT GluN1/GluN2A and 146 ± 20 and 689 ± 49 ms for diheteromeric GluN1/GluN2A-P552R, respectively (p < 0.0001 for both, t-test, n = 7–9). The rise time and weighted tau for receptors with 0, 1, or 2 copies of GluN2A-P552R were 8 ± 1and 38 ± 3.4 ms, 9 ± 1 and 201 ± 30 ms, 210 ± 16 and 1283 ± 153 ms, respectively (n = 8). **D**, Whole cell voltage clamp recording of di-heteromeric (*left panels*) and tri-heteromeric (*right panels*) GluN1/GluN2A-P552R receptor responses to rapid application (1.5 sec) of glycine; saturating glutamate (100 μM) was present in all of the solutions. See Table 4 for all mean ± s.e.m. values and S5 Table for statistical analyses for panels **A-D**. **E**, Single channel currents were recorded from HEK293 Tet-On cells selectively expressing GluN1/GluN2A receptors with 0, 1 or 2 copies of GluN2A-P552R. Currents were recorded in the outside-out configuration at -80 mV. The closed state (C) is indicated by a dashed line and openings are downwards deflections of current and the open state is indicated by an O. **F**, Normalized open time histograms from the three current recordings shown in (**E**) reveal an increase in the slower open time component of GluN1/GluN2A-P552R. Smooth lines show fitted dual component exponential functions. See also S2 Fig.

Analysis of glutamate concentration-response curves for GluN1/GluN2A-P552R yielded an EC_50_ value for glutamate that was 10-fold lower (i.e. 10-fold more potent) than that observed for WT GluN1/GluN2A receptors (Fig 4A) (Table 4). Similar analysis of the glycine concentration-response relationship suggested that the EC_50_ for glycine was 20 times lower (i.e. more potent) at NMDARs containing GluN2A-P552R compared to WT GluN2A (Fig 4B) (Table 4). Because the patient has a single copy of the mutation, it is likely that the NMDARs in this patient will be a mixture of those with 0, 1 or 2 copies of the mutant GluN2A subunit. We have previously described a pair of modified GluN2A subunits that contain complementary sets of coiled-coil domains followed by an endoplasmic reticulum (ER) retention signal [32,33]. Use of this pair of constructs allows us to control the identity of the two GluN2A subunits within each NMDAR complex at the surface of the cell. To compare the effects of a single copy of GluN2A-P552R to receptors with two copies of GluN2A-P552R, we used this system to express tetrameric GluN1/GluN2A receptors that contained 0, 1, or 2 copies of GluN2A-P552R, and evaluated glutamate and glycine potency at these receptors. Receptors with one copy of this mutation showed an intermediate increase in glutamate and glycine potency (Fig 4A and 4B) (Table 4), confirming that a single copy of the mutant subunit can significantly alter agonist potency.

**Table 4:** Pharmacological and functional properties of di-heteromeric and tri-heteromeric GluN2A-P552R receptors.

	Di-heteromeric NMDA Receptors		Tri-heteromeric NMDA Receptors		
	GluN2A	GluN2A-P552R	GluN2A/GluN2A	GluN2A-P552R/ GluN2A	GluN2A-P552R/ GluN2A-P552R
**Glutamate, EC**_**50**_, **μM (n)**	3.3 ± 0.35 (9)	0.37 ± 0.04 (13)*	3.9 ± 0.26 (32)	1.2 ± 0.13 (23)^#^	0.64 ± 0.05 (25)^#^^$^
**Glycine, EC**_**50**_, **μM (n)**	1.2 ± 0.07 (10)	0.06 ± 0.01 (10)*	1.1 ± 0.04 (26)	0.24 ± 0.02 (28)^#^	0.08 ± 0.08 (20)^#^^$^
**Amplitude (peak, pA/pF)**	94 ± 26	90 ± 17	53 ± 24	56 ± 16	52 ± 18
**Amplitude (SS, pA/pF)**	60 ± 17	---	38 ± 17	49 ± 16	---
**I**_**SS**_**/I**_**PEAK**_**%**	67 ± 3.8%	---	71 ± 3.9%	83 ± 6.2%	---
**Rise time (ms)**	9.3 ± 0.9	576 ± 62*	8.8 ± 1.1	11 ± 1.1	1098 ± 26^#^^$^
**τ**_**FAST**_ **(ms)**	39 ± 5.0	780 ± 73	42 ± 6.8	171 ± 31	1474 ± 124^#^^$^
**τ**_**SLOW**_ **(ms)**	543 ± 119	3383	318 ± 71	845 ± 163^#^	4812 ± 1179^#^^$^
**%τ**_**FAST**_	96 ± 1.4%	99 ± 0.4%	97 ± 1.4%	77 ± 5.5%	96 ± 1.9%
**τ**_**W**_**(ms)**	52 ± 4.6	794 ± 71*	49 ± 5.1	279 ± 29	1585 ± 110^#^^$^
**Charge transfer pA × ms/pF**	4,378 ± 1,184	33,130 ± 7,553*	2,578 ± 1,094	13,460 ± 4,034	30,514 ± 14,400^#^
**n**	9	6	8–9	7–8	6–9
**Mean Open Time (ms)**	---	---	2.3 ± 0.20	2.3 ± 0.18	5.0 ± 0.33^#^^,^^$^
**Current Amplitude (pA)**	---	---	-5.9 ± 0.09	-6.1 ± 0.16	-5.0 ± 0.04^#^^,^^$^
**Conductance (pS)**	---	---	74 ± 1.2	77 ± 1.9	62 ± 0.5^#^^,^^$^
**Open τ1 (ms)**	---	---	0.07 ± 0.007	0.06 ± 0.006	0.15 ± 0.04^$^
**Open τ2 (ms)**	---	---	2.9 ± 0.23	2.9 ± 0.21	5.5 ± 0.32^#^^,^^$^
**Open Area 1 (%)**	---	---	0.22 ± 0.02	0.22 ± 0.02	0.10 ± 0.02^#^^,^^$^
**Open Area 2 (%)**	---	---	0.78 ± 0.02	0.78 ± 0.02	0.90 ± 0.02^#^^,^^$^
**n**	---	---	8	12	11
**Open probability**	0.17 ± 0.01	0.20 ± 0.02	---	---	---
**n**	12	27	---	---	---

Human GluN1 and GluN2 were used for diheteromeric receptors and rat GluN1 and GluN2 were used for triheteromeric experiments. Open probability was assessed as described in the ***Methods*** using the potentiation by MTSEA of GluN1 mutant subunit.

* p < 0.001 compared to WT GluN2A, unpaired t-test

^#^ p < 0.05 compared to GluN2A/GluN2A; one way ANOVA, Tukey post hoc

^$^ p < 0.05 compared to GluN2A-P552R/GluN2A; one way ANOVA, Tukey post hoc

See S5 Table for F statistics and p values.

### GluN2A-P552R pre-M1 mutation slows the response time course

In order to assess the actions of this mutation on NMDAR kinetics, we expressed WT GluN1/GluN2A and GluN1/GluN2A-P552R receptors in HEK293 cells and measured the time course of the whole cell current responses to a maximally effective concentration of glutamate applied for either a long or brief duration. In these experiments, the solution exchange time around the cell (10–90% rise time) was approximately 4 ms [34], and glycine was present at saturating concentration (30–100 μM) in all solutions unless otherwise indicated. Both the activation and deactivation time course were dramatically slower in receptors containing GluN2A-P552R compared to WT GluN2A. GluN2A-P552R-containing receptors had an almost 60-fold increase in the 10–90% rise time in response to prolonged glutamate application (*left panels*, Fig 4C) (Table 4). The deactivation time course following removal of glutamate for both WT GluN2A and GluN2A-P552R could be adequately fitted by a dual exponential decay. Tau_FAST_ was slowed 25-fold and tau_SLOW_ increased almost 3-fold for GluN2A-P552R compared to WT receptors (*left panels*, Fig 4C) (Table 4).

For brief (5 ms) glutamate application, the receptor-mediated current response continued to rise after the glutamate application had terminated and the cell had been returned to glutamate-free wash solution. Hence glutamate was able to bind during the 5 ms application, which suggests that the slow current response time course reflects a slowing of conformational changes that precede channel opening rather than slow glutamate binding. The deactivation time course following removal of glutamate for both WT GluN2A and GluN2A-P552R could be adequately fitted by a dual exponential decay. Tau_FAST_ was slowed 25-fold and tau_SLOW_ increased almost 3-fold for GluN2A-P552R compared to WT receptors (*left panels*, Fig 4C). Similar changes in the response time course were obtained when 30 μM glycine was rapidly applied to activate the receptor (in the presence of 100 μM glutamate) (*left panels*, Fig 4D) (S2 Table). Charge transfer during synaptic transmission can be determined from the integral of experimentally recorded EPSCs, which can be approximated by an instantaneously rising and exponentially decaying function. The integral of this function is the product of the amplitude and the weighted time constant describing exponential decay. We thus used the response amplitude and deactivation time courses measured from the response to brief 5 ms pulse of glutamate to estimate the synaptic charge transfer, which was markedly increased for GluN2A-P552R compared to WT GluN2A (Table 4). These data suggest that this mutation in the pre-M1 region alters the channel activation rate, agonist dissociation, and synaptic function.

Receptors with either 1 or 2 copies of the mutation had a slower deactivation time course, which increased the predicted synaptic charge transfer. Unexpectedly, we found that a single copy of GluN2A-P552R mutation did not slow the response rise time, even though the deactivation time course was slower (*right panels*, Fig 4C) (Table 4). By contrast, receptors with two copies of GluN2A-P552R showed both slow rise time and slow deactivation time course. A similar result was obtained when we pre-equilibrated the cells with 100 μM glutamate and activated the receptor by rapidly applying 30 μM glycine (which binds GluN1) with 100 μM glutamate (Fig 4D) (S2 Table). These data suggest non-equivalent contribution to gating by the two GluN2 pre-M1 helices within each NMDAR complex.

### NMDARs with 2 GluN2A-P552R subunits have altered single channel properties

To determine whether the pronounced alterations in the response time course to glutamate and glycine could be understood in terms of single channel properties, we performed single channel recordings from outside-out patches with NMDAR channels that contained 0, 1, or 2 copies of the GluN2A-P552R mutant subunit (see Methods). Analysis of single channel currents showed that a single copy of this mutant subunit did not alter mean open time or chord conductance of the channel (Fig 4E and 4F and S2 Fig) (Table 4). Furthermore, there was no change in the fitted time constants describing the dual component open duration histogram. By contrast, when two copies of GluN2A-P552R were included in the receptor complex, we observed a significant increase in the mean open time (5.0 ± 0.3 ms for GluN2A-P552R/GluN2A-P552R *vs*. 2.3 ± 0.2 ms for GluN2A/GluN2A and 2.3 ± 0.2 ms GluN2A-P552R/GluN2A), which reflected a slowing of both the fast and slow components describing the open time distribution (Fig 4E and 4F and S2 Fig) (Table 4). In addition, the chord conductance was significantly reduced for two copies of GluN2A-P552R (GluN2A-P552R/GluN2A-P552R: 62 ± 0.5 pS) channels compared to WT GluN1/GluN2A (GluN2A/GluN2A: 74 ± 1.2 pS) and a single copy of GluN2A-P552R channels (GluN2A-P552R/GluN2A: 77 ± 1.9 pS) (Fig 4E and 4F) (Table 4). Although the determinants of conductance in NMDARs are poorly understood, these data suggest that GluN2A-P552R can alter the stability and conformation of the open pore or its access portals only when both GluN2A subunits harbor the Pro552Arg mutation in a functional receptor. The altered single channel conductance might reflect sustained contact, for example, of the M3 transmembrane helix with the mutant pre-M1 helix, which may adopt a different position than WT pre-M1 in the open state thereby altering the position or electrostatic charge of the pore-lining M3 transmembrane helix. Estimates of open probability from MTSEA modification of GluN1(A652C) subunit co-expressed with GluN2A (see Methods) in oocytes did not reveal a detectable difference (Table 4), consistent with the opposing effects of GluN2A-P552R on mean open time and chord conductance (Table 4), as well as the lack of effect of the mutation on the mean current amplitude (Table 4). These data suggest that the primary effect of GluN2A-P552R on macroscopic currents is to dramatically slow and prolong the time course of the current response.

### GluN2A-P552R enhances voltage-independent Zn^2+^inhibition but not Mg^2+^ or proton block

An important property of NMDARs is that they are blocked by extracellular Mg^2+^ at resting membrane potentials in a voltage-dependent manner [35,36]. Evaluation of the potency with which extracellular Mg^2+^ blocked NMDAR showed similar sensitivity to Mg^2+^ for GluN2A-P552R as for WT GluN2A (S3 Table). We also evaluated the potencies of two additional endogenous modulators of NMDAR function, extracellular Zn^2+^ and protons (H^+^) [37,38]. Zn^2+^ potency was modestly increased (i.e. IC_50_ values decreased) two-fold at GluN1/GluN2A-P552R, and the maximal degree of voltage-independent inhibition by Zn^2+^ increased from 60% at GluN2A to 90% at GluN2A-P552R. There were no detectable effects of GluN2A-P552R on the proton sensitivity of the NMDARs (S3 Table), which represents an intriguing property given the strong coupling between downstream mechanisms that mediate proton and Zn^2+^ inhibition [39–42].

### Functional conservation of pre-M1 proline within the GluN2 subunit

**Fig 5:**
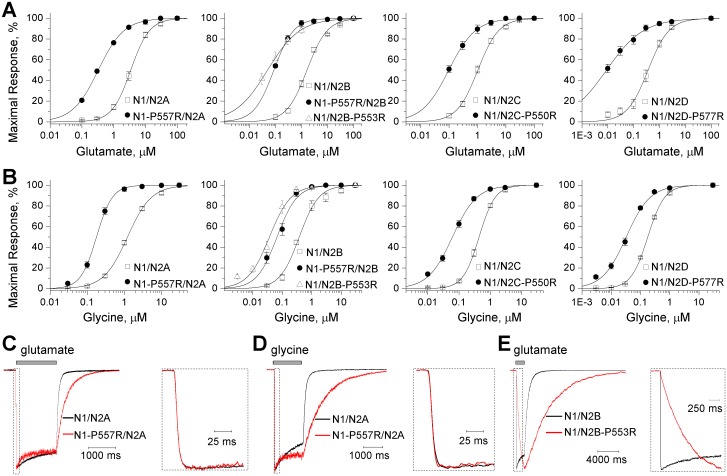
Conserved effects of the Pro552Arg mutation across GluN2 subunits. **A**,**B**, Composite concentration-response curves of glutamate in the presence of 100 μM glycine (**A**) and glycine in the presence of 100 μM glutamate (**B**) for human GluN1-P557R/GluN2A, GluN1-P557R/GluN2B, GluN1/GluN2B-P553R, and rat GluN1/GluN2C-P550R, and GluN1/GluN2D-P577R. The graph legends refer to GluN1 as N1 and GluN2 as N2. Fitted EC_50_ values are summarized in Tables 3 and 5. **C**,**D**, human GluN1-P557R/GluN2A significantly prolongs deactivation time course after removal of glutamate (**C**) or removal of glycine (**D**) on transfected HEK293 cells, but does not slow the rise time when the receptors were activated by the agonists. **E**, GluN1/GluN2B-P553R significantly slows rise time and prolongs deactivation time course. Fitted parameters describing the response time course are given in Table 6.

A number of structural features of the NMDAR pore and key gating elements are strongly conserved across the glutamate receptor family [5,10,12,18,33]. The *de novo* missense mutations studied here at the Pro residue in GluN1, GluN2A, and GluN2B pre-M1 helix occur at a position conserved across all NMDAR subunits. Moreover, the pre-M1 region is depleted of missense variants in the control cohort of exomes across the NMDAR subunits, further suggesting that this Pro residue and the pre-M1 region play a conserved and critical role across all NMDARs. To test this idea, we substituted Arg for the similarly positioned Pro in GluN1-P557R, GluN2B-P553R, GluN2C-P550R, GluN2D-P580R and tested whether these mutations had similar effects on NMDAR pharmacology and response time course. We first evaluated the agonist concentration-effect curves for all mutations. Similar to GluN2A-P552R (Table 4), each Pro-Arg mutation increased both the glutamate and glycine potency (Fig 5A and 5B) (Tables 3 and 5). We performed whole-cell patch recording of the current response to rapid application of maximally effective glutamate (in the presence of saturating glycine). When Arg was substituted for the conserved Pro in GluN1, the deactivation time course following glutamate removal or glycine removal was slowed similar to GluN2A-P552R. However, in contrast to GluN2A-P552R, NMDARs containing GluN1-P557R showed a rapid response rise time to both glutamate and glycine (Fig 5C and 5D) (Table 6). These results suggest that perturbations in the GluN1 pre-M1 helix do not detectably alter the rate at which NMDARs reach the open state after agonist binding. We also evaluated the response time course for NMDARs that contained GluN2B-P553R. Similar to GluN2A-P552R, this mutation slowed both response rise time and deactivation time course following glutamate removal (Fig 5E) (Table 6). The differential effect of GluN1 and GluN2 Pro-Arg pre-M1 mutations observed on rise time suggest distinct pre-gating conformational changes in the pre-M1 region for GluN1 and GluN2 subunits.

**Table 5:** Agonist potency of GluN2A-P552R equivalent mutations in GluN2B, GluN2C, and GluN2D.

	Glutamate, EC_50_, μM (n)	Glycine, EC_50_, μM (n)
**GluN1/GluN2B**	1.6 ± 0.11 (12)	0.45 ± 0.04 (6)
**GluN1/GluN2B-P553R**	0.07 ± 0.03 (8)*	0.05 ± 0.01 (8)*
**GluN1/GluN2C**	1.1 ± 0.12 (8)	0.45 ± 0.04 (10)
**GluN1/GluN2C-P550R**	0.11 ± 0.02 (6)*	0.06 ± 0.01 (8)*
**GluN1/GluN2D**	0.4 ± 0.07 (7)	0.18 ± 0.01 (7)
**GluN1/GluN2D-P577R**	0.01 ± 0.002 (5)*	0.03 ± 0.004 (7)*

Data are from human GluN1/GluN2B, rat GluN1/GluN2C, and rat GluN1/GluN2D

* p < 0.001 compared to corresponding WT NMDAR, unpaired t-test

**Table 6:** Glutamate response time course of GluN2A-P552R equivalent amino acid changes in GluN1 and GluN2B.

	WT GluN1/GluN2A	GluN1-P557R/GluN2A	WT GluN1/GluN2B	GluN1/GluN2B-P553R
**Amplitude (peak, pA/pF)**	83 ± 29	9.9 ± 4.2*	31 ± 6.9	24 ± 13
**Amplitude (SS, pA/pF)**	52 ± 16	8.4 ± 3.5*	23 ± 5.3	---
**I**_**SS**_**/I**_**PEAK**_**%**	68 ± 3.0%	89 ± 4.1%*	74 ± 2.5%	---
**Rise time (ms)**	10 ± 0.8	10 ± 1.7	15 ± 3.2	1033 ± 28*
**τ**_**FAST**_ **(ms)**	39 ± 5.1	337 ± 37*	410 ± 62	2992 ± 450*
**τ**_**SLOW**_ **(ms)**	559 ± 120	696 ± 108	1370 ± 185	8797 ± 2764*
**%τ**_**FAST**_	96 ± 1.7%	81 ± 6.0%*	62 ± 6.0%	63 ± 13%
**τ**_**W**_ **(ms)**	54 ± 5.7	395 ± 32*	765 ± 66	4590 ± 417*
**n**	9	8	11	8

Data are from human NMDARs.

* p < 0.05 compared to corresponding WT, unpaired t-test.

See S7 Table for p values.

**Fig 6:**
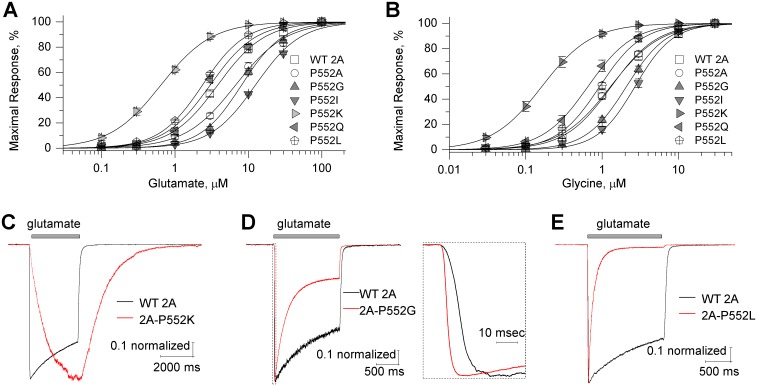
Assessment of alternative Pro552 substitutions in GluN2A. **A,B**, the composite glutamate (in the presence of 100 μM glycine) and glycine (in the presence of 100 μM glutamate) concentration-response curves of GluN2A- P552A, P552G, P552I, P552K, P552Q, P552L constructs. Error bars are SEM and shown when larger than symbol. **C,D,E**, The response time courses are shown of GluN1/GluN2A(P552K), GluN1/GluN2A(P552G), and GluN1/GluN2A(P552L) receptors activated by rapid application of 100 μM glutamate; 100 μM glycine was present in all solutions. For panel **D** the rise time is expanded as an inset. The data (mean ± SEM) are given in Table 7.

The substitution of an arginine for a proline brings about multiple changes to the amino acid side chain at position 552, including a change in size, charge, and hydrogen binding capacity, as well as an alteration of polypeptide chain flexibility. To assess possible features of this residue that could influence receptor function, we separately evaluated the effects of mutations that altered the hydrogen bonding properties of the side chain (Arg, Lys, Gln) or the size of the side chain (Gly, Ala, Ile, Leu) substituted for Pro on glutamate and glycine potency of NMDARs. We found that the glutamate potency for GluN1/GluN2A-P552L and GluN1/GluN2A-P552Q were not detectably altered compared to WT receptors (Fig 6A and 6B) (Table 7). Receptors comprised of GluN1 co-expressed with GluN2A-P552A, GluN2A-P552G, or GluN2A-P552I all showed modestly reduced potency of glutamate. By contrast, glutamate (0.66 μM) and glycine (0.19 μM) potency was markedly enhanced for GluN1/GluN2A-P552K receptors (Fig 6A and 6B) (Table 7). Whereas substituting residues with side chains of similar volume did not enhance glutamate potency, substitution of a residue (Lys) with an ionizable amino group had a similar effect on potency as the mutation (Arg) with an ionizable guanidino group, suggesting an important role for the charge of this side chain (Table 7).

**Table 7:** Pharmacology and response time course for substitution of GluN2A-ProP552 with Ala, Gly, Lys, Gln, Ile, or Leu.

	WT 2A	P552Q	P552K	WT 2A	P552G	P552A	P552I	P552L
**Glutamate, EC**_**50**_, **μM (n)**	4.0 ± 0.27 (7)	3.1 ± 0.30 (6)	0.66 ± 0.07 (6)^#^		8.2 ± 0.51 (6)^#^	6.9 ± 0.74 (6)^#^	13 ± 0.3 (6)^#^	2.4 ± 0.12 (6)
**Glycine, EC**_**50**_, **μM (n)**	1.4 ± 0.20 (9)	0.80 ± 0.13 (14)^#^	0.19 ± 0.02 (15)^#^		2.1 ± 0.14 (16)^#^	1.4 ± 0.08 (13)	2.8 ± 0.24 (13)^#^	0.94 ± 0.06 (6)
**Amplitude (peak, pA/pF)**	131 ± 23	35 ± 6.4^#^	5.8 ± 2.9^#^	96 ± 17	101 ± 21	115 ± 21	46 ± 13	61 ± 23
**Amplitude (SS, pA/pF)**	74 ± 15	5.4 ± 0.9^#^	---	52 ± 9.5	21 ± 5.5^#^	33 ± 7.7^#^	14 ± 3.9^#^	0.7 ± 0.13^#^
**I**_**SS**_**/I**_**PEAK**_**%**	56 ± 5.0%	18 ± 2.1%^#^	---	58 ± 3.7%	20 ± 2.3%^#^	29 ± 4.8%^#^	29 ± 2.7%^#^	1.5 ± 0.1%^#^
**τ**_**W**_ **desensitization (ms)**	735 ± 76	211 ± 21^#^	---	769 ± 52	235 ± 32^#^	374 ± 59^#^	742 ± 120	91 ± 4.5^#^
**Rise time (ms)**	7.7 ± 0.51	5.7 ± 0.7	1063 ± 14^#^	10 ± 0.9	5.2 ± 0.4^#^	11 ± 1.1	8.3 ± 0.9	11 ± 1.5
**τ**_**FAST**_ **(ms)**	36 ± 3.3	32 ± 2.0	555 ± 47^#^	37 ± 2.9	16 ± 1.5^#^	26 ± 1.3	17 ± 1.4^#^	21 ± 4.4^#^
**τ**_**SLOW**_ **(ms)**	271 ± 85	315 ± 60	---	583 ± 85	416 ± 138	705 ± 188	384 ± 118	123 ± 32^#^
**%τ**_**FAST**_	94 ± 1.9%	86 ± 2.1%	95 ± 3.7%	91 ± 4.4%	90 ± 2.7%	96 ± 1.3%	92 ± 6.8%	79 ± 6.8%
**τ**_**W**_**(ms)**	47 ± 5.7	65 ± 6.5	610 ± 40^#^	55 ± 4.2	37 ± 6.1	46 ± 5.8	25 ± 2.8^#^	40 ± 5.7
**n**	8	11	9	22	12	10	8	12

Human GluN1 and GluN2 were diheteromeric receptors and rat GluN1 and GluN2 were used for triheteromeric experiments.

^#^ p < 0.05 compared to GluN2A/GluN2A; one way ANOVA, Tukey post hoc.

See S8 Table for F statistics and p values.

To elucidate the effect of these two groups of mutations on response time courses of NMDARs, including activation time, deactivation time, and desensitization, we measured whole cell current responses of HEK293 cells expressing the mutant receptors. The GluN2A-P552K mutation produced a delay in rise time compared to WT receptors, consistent with the delay observed for GluN2A-P552R. Similarly, receptors containing GluN2A-P552K deactivated with a time course 12-fold slower than that of WT receptors, closely resembling that measured for the receptors containing the Arg mutation (Fig 6C) (Table 7). Retention of hydrogen binding capacity without charge in GluN2A-P552Q altered response amplitude and some features of desensitization, but did not detectably alter the response rise time or deactivation time course. Only GluN2A-P552G, which reduced side chain volume and increased chain flexibility, accelerated the response activation time (10–90% rise time 5.2 ± 0.4 ms) compared to the WT receptors (10 ± 0.9 ms) (Fig 6D) (Table 7). The fast time constant describing deactivation was accelerated by changes of this Pro to Gly, Ile, and Leu. Mutations of Pro to Ala, Gly, Ile, Leu as well as Gln increased the degree of desensitization relative to WT GluN2A, achieving steady-state levels that were only 20%, 29%, 29%, 1.5%, and 18% of the peak response, respectively, compared to 56% for WT GluN1/GluN2A (Fig 6D) (Table 7). This is consistent with a recent report showing strong effects on receptor desensitization by residues in the pre-M1 region [29]. NMDARs containing GluN2A-P552L showed no change in surface expression (S1 Fig), but underwent profound desensitization, showing greater than 98% reduction in current at steady-state (Fig 6E) (Table 7). Notably, the slow response rise time prevented receptors containing Arg and Lys from reaching steady state during even prolonged agonist application, and therefore, desensitization of these mutant receptors could not be determined.

### Pre-M1 Pro-Arg mutation slows and prolongs the EPSP

**Fig 7:**
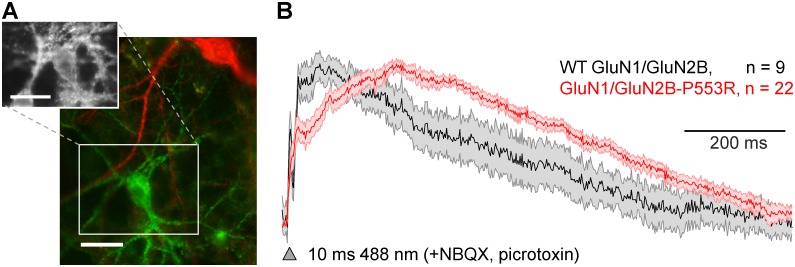
Effect of GluN2B-P553R on the NMDAR component of the EPSP. **A**, Red shows the presynaptic neuron expressing a channelrhodopsin variant (CheRiff) and green shows postsynaptic neuron transfected with GFP-GluN2B or GFP-GluN2B-P553R and a QuasAr voltage indicator (inset). Scale bars, 30 μm. **B**, Optically-evoked and optically-monitored EPSPs. The mean postsynaptic EPSP is given for neurons expressing GFP-GluN2B-P553R (red) or GFP-GluN2B (black). Shading represents s.e.m; n = number of neurons. EPSPs are shown normalized to peak (see Methods).

To determine whether the changes in the NMDAR time course produced by mutations in the pre-M1 helix could impact synaptic transmission in neurons, we made optical recordings of EPSPs in cultured neurons transiently transfected with either GFP-tagged GluN2B or GFP-tagged GluN2B-P553R. We produced a mixed culture containing neurons expressing either a channelrhodopsin optogenetic actuator (CheRiff) or a fluorescent voltage sensor (QuasAr) and the GFP-tagged NMDAR (see Methods; *Linlin Z*. *Fan*, *Ralda Nehme*, *Yoav Adam*, *Hao Wu*, *Eun Sun Jeung*, *Donald B*. *Arnold*, *Kevin Eggan*, *Adam E*. *Cohen*, *“All-optical electrophysiology of synaptic transmission and synaptic plasticity” in submission*). QuasAr voltage indicators have been shown to report membrane voltage changes in cultured neurons with good fidelity and linearity [43]. Optical stimulation of the presynaptic cells evoked EPSPs which were detected via fluorescence from the postsynaptic cells. Optical recordings of the EPSP closely follow conventional electrical current clamp recordings of membrane voltage during the EPSP (Fig 7, S7 Fig). To isolate the effects of NMDARs, measurements were performed in the presence of blockers for AMPA and GABA receptors (see Methods). Inhibitory neurons express GluN2A, GluN2B, and GluN2D subunits while excitatory neurons predominantly express GluN2A and GluN2B. Immunostaining revealed that only 5.3% of the neurons in the culture were inhibitory, establishing that our data almost exclusively probed synaptic transmission onto excitatory glutamatergic post-synaptic neurons.

We observed a pronounced slowing of the 20–80% rise time of the NMDAR-component EPSP from 18 ms for GFP-GluN2B (*n* = 9 neurons) to 108 ms for GFP-GluN2B-P553R (*n* = 22 neurons), which was consistent with the slowing of the rise time for voltage clamp current response to brief synaptic like application of glutamate in HEK293 cells transfected with GluN2A-P552R. In addition, the half-width of the NMDAR-mediated EPSP was prolonged from 357 ms for GFP-GluN2B transfected neurons to 557 ms for GFP-GluN2B-P553R neurons (Fig 7). These data confirm that the properties we observed in heterologous expression systems under voltage-clamp conditions influence synaptic signaling under physiological conditions where the voltage is not clamped and the response can be influenced by voltage-dependent channels.

### Pre-M1 GluN2A-P552R mutation induces neurotoxicity

**Fig 8:**
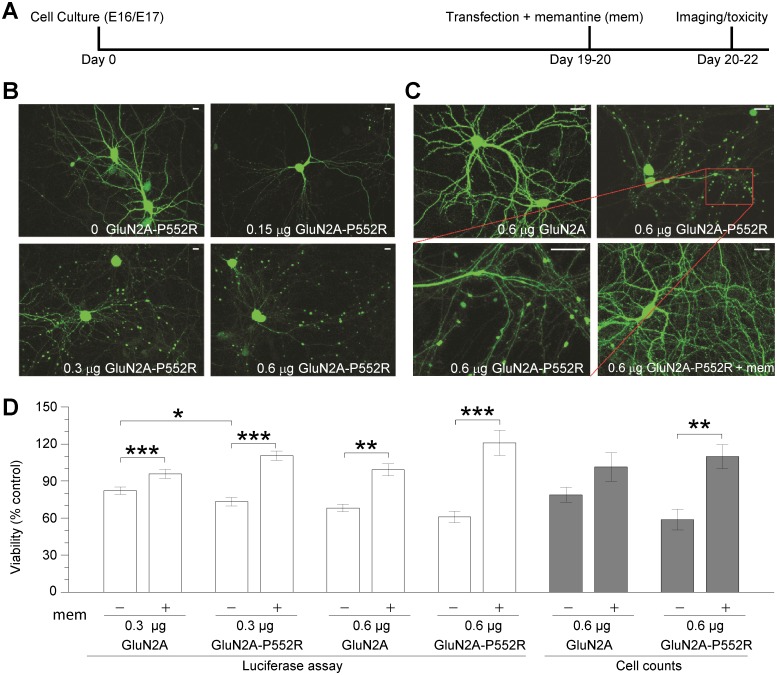
Neurotoxic consequences of GluN2A-P552R expression and rescue pharmacology. **A**, Schematic of experimental timeline indicates the relative dates of neuronal cell culture from embryonic day 16/17 (E16/17), transfection along with memantine/vehicle treatment, and toxicity studies (luciferase assays, cell counts, and confocal imaging). **B**, Confocal images of cortical neurons transfected with a GFP-expressing construct, with various concentrations of the human GluN2A-P552R-expressing plasmid illustrate the cell morphology. Images were acquired 24 h post-transfection at 20× magnification (scale bars 10 μm). **C**, Confocal images of cortical neurons transfected with GFP-expressing construct with either 0.6 μg cDNA per well (40% of total transfection cDNA) of WT GluN2A or GluN2A-P552R, the latter in the absence or presence of memantine (50 μM). Images were acquired 24 h post-transfection at 20× magnification, with the exception of the bottom left panel (40×), which highlights GluN2A-P552R-induced dendritic swelling and blebs (scale bar 10 μm). **D**, The mean cell viability values are shown as a percent of control. Luciferase assays: neuronal cultures were transfected with GFP-N1 plasmid (0.525 μg or 0.825 μg per well) luciferase cDNA (0.375 μg/well) for cell viability assaying, with varied concentrations (0.3 μg or 0.6 μg per well) of pCIneo-vector, WT GluN2A, or GluN2A-P552R cDNA (1.5 μg total DNA per well). Each transfection condition was performed in pairs, either supplemented with vehicle (–) or memantine (20 μM for 0.3 μg; 50 μM for 0.6 μg) treatment (+). Luciferase assays were performed 48 h following transfection and treatment. Experiments were performed in quadruplicate, and independent experiments were repeated (0.3 μg cDNA, n = 7; 0.6 μg cDNA, n = 8). Each condition was normalized to its relevant vector-transfected group to obtain relative viability values, expressed as % control. Data are mean ± SEM of viability (% control) for each condition (ANOVA/Bonferroni; *p <0.05, **p < 0.01, ***p < 0.001). Cell counts: Neuronal cultures were transfected with GFP-N1 plasmid for cell visualization, with either 0.6 μg pCIneo including vector, WT GluN2A, or GluN2A-P552R cDNA (40% of total transfection cDNA). Each transfection condition was performed in duplicate. Cell counts were performed 48 h post-transfection (Methods). Data are mean ± SEM of viability (% control) for each condition in 6 independent experiments. ANOVA/Bonferroni (**p < 0.01). See S9 Table for statistics.

A cerebral CT-scan of the patient with GluN2A-P552R mutation showed pronounced peripheral liquor spaces, mainly in the frontal region [20], suggesting that expression of the mutant GluN2A-P552R subunit is associated with a loss of grey matter. The increased charge transfer associated with mutant GluN2A-P552R receptors raises the possibility that this mutation could be neurotoxic by exaggerating Ca^2+^ influx during synaptic transmission, which may contribute to observed neuropathological changes. Neurotoxicity caused by this mutation could lead to spine loss or parenchymal cell loss, possibly underlying certain features of the patient’s neurologic condition. Because GluN2A-P552R had no detectable effect on surface expression, we were able to compare expression of WT GluN2A with GluN2A-P552R in neurons. To test whether this mutation *per se* was neurotoxic, we transfected cultured cortical neurons with GFP, WT human GluN2A or GluN2A-P552R. We also co-transfected a luciferase construct, which allowed quantification of the proportion of live cells [44], and corroborated the results by automated cell counts in GFP-co-transfected cells. Co-transfection of the mutant GluN2A-P552R subunit produced rapid, highly pronounced swelling of dendritic processes (blebs) in a plasmid concentration-dependent manner (0.0–0.6 μg DNA per tissue culture well; Fig 8A–8D). The blebbing was prominent primarily in GluN2A-P552R-transfected neurons, and virtually absent in GFP or WT GluN2A transfected neurons; this trend was observed without exception in all experiments (see S4 and S5 Figs). The swelling observed in cultures transfected with 0.3 μg mutant GluN2A-P552R cDNA was accompanied by a significant decrease in viability when compared to WT GluN2A. Nonetheless, this difference in toxicity between mutant and WT receptor was not discernible at the higher DNA concentrations used (e.g. 0.6 μg; Fig 8D). However, overexpression of WT GluN2A cDNA seldom produced any morphological changes in the dendrites, even at high plasmid concentrations (0.6 μg; Fig 8C, S4 Fig), suggesting that cell death and dendritic blebbing might be mediated by two separate neuropathological processes.

We tested whether FDA-approved NMDAR antagonists could be neuroprotective against the neurotoxic effect observed. We first evaluated the IC_50_ for inhibition of mutant and WT GluN1/GluN2A receptors by channel blockers memantine, dextromethorphan, dextrorphan, amantadine, and ketamine as well as the non-competitive GluN2A-selective inhibitor TCN-201, which binds at the ligand binding domain dimer interface to reduce glycine affinity [45]. Based on the mechanism of action of TCN-201, we predicted that the 20-fold increase in the glycine potency in GluN2A-P552R would render TCN-201 ineffective in the presence of 3 μM glycine, which was confirmed by our results (Table 4). By contrast, the channel blockers all inhibited recombinant NMDAR responses with similar potency at WT and mutant GluN1/GluN2A receptors in the absence of Mg^2+^ (Table 8) (S3 Fig). We subsequently tested a concentration of memantine that was several-fold higher than the IC_50_ value observed in the presence of Mg^2+^ for GluN1/GluN2A receptors (13 μM) [46] for neuroprotective actions, since the culture media contains extracellular Mg^2+^. Inclusion of 20–50 μM memantine in the culture media following co-transfection with mutant GluN2A reduced swelling of dendrites in all cultures (Fig 8C; 4 of 4 experiments) and rectified the decrease in viability (Fig 8D), suggesting that memantine was neuroprotective in cultured neurons against the neurotoxic actions of GluN2A-P552R.

**Table 8:** Potency and efficacy of NMDAR inhibitors on GluN1/GluN2A-WT and -P552R.

Drug	IC_50_ (n; max inhibition)		
	GluN2A-WT	GluN2A-P552R	p-value^#^
**Memantine**	4.8 ± 0.4 (10; 96%)	3.7 ± 0.7 (9; 98%)	0.09
**Dextromethorphan**	18 ± 2.9 (11; 94%)	15 ± 4.6 (10; 93%)	0.108
**Dextrorphan**	1.1 ± 0.2 (12; 97%)	0.90 ± 0.2 (6; 95%)	0.092
**Amantadine**	126 ± 17 (9; 88%)	86 ± 22 (9; 94%)	0.051
**Ketamine**	4.7 ± 0.8 (7; 94%)	1.3 ± 0.2 (6; 96%)	< 0.001
**TCN 201**	0.18 ± 0.01 (9; 97%)	>10 (8; 35%)	-

The data are expressed as mean IC_50_ value, μM ± SEM (n, maximal inhibition % at 100 μM for memantine, 300 μM for dextromenthorphan, 30 μM for dextrorphan, 1000 μM for amantadine, 100 μM for ketamine, and 10 μM for TCN-201).

^**#**^ p-values for unpaired t-tests comparing logIC_50_ values for WT vs. P552R.

## Discussion

In this study, we analyzed the intolerance of genetic variation in protein domains within GluN1, GluN2A and GluN2B subunits. Our analysis indicates the pre-M1 regions of the GluN2 subunits are among the most heavily missense depleted sub-regions of the protein-coding sequence of the relevant genes. We further evaluated NMDAR functional changes caused by five *GRIN* missense mutations in the pre-M1 region of the receptor found in children with developmental delay, intellectual disability, and/or epilepsy (Table 2). Among these mutations, we focused on the mutant GluN2A-P552R, since this Pro residue is conserved across GluN subunits in multiple species (Fig 2; [29]). Our data show that GluN2A-P552R should be considered a gain-of-function variant because it shows increased glutamate / glycine potency and pronounced slowing of the glutamate current response time course, without concomitant changes in receptor surface expression or response amplitude. In the absence of extracellular Zn^2+^, the sum of these effects is predicted to cause a ~10-fold increase in charge transfer upon a brief synaptic-like pulse of glutamate for receptors. The modest increase in Zn^2+^ inhibition will not circumvent this gain-of-function. The increased charge transfer led us to hypothesize that GluN2A-P552R receptors expressed after birth should damage neuronal health by causing NMDAR overactivation and excessive Ca^2+^ entry into the neuron, which may contribute to the clinical manifestation of this condition. Consistent with this hypothesis, we observed swollen dendrites in all cultured cortical neurons exogenously expressing GluN2A-P552R but not WT GluN2A, which could be prevented by inclusion of memantine in the culture media. Together, our data implicate neuronal damage caused by overactivation of NMDARs in the pathophysiology of GluN2A-P552R mutations.

The Pro residue in the pre-M1 helix is conserved across the NMDAR subunit family, and the corresponding Pro-Arg mutation in GluN1, GluN2B, GluN2C, and GluN2D brings about a similar enhancement in glutamate and glycine potency. For GluN2B, slowing of the response time course is similar to that observed for GluN2A, arguing that this GluN2 Pro plays a similar role in receptor activation throughout the GluN2 subunits. Given this apparent functional conservation within the GluN2 family, it is not surprising that other disease-causing mutations have been found at this site in both the GluN1 and GluN2 pre-M1 helix in NMDAR subunits (Table 2), including GluN2B-P553L [20] and GluN1-P557R [23], leading us to further investigate the scope of function of the pre-M1 cuff helix. Evaluation of these human mutations showed strong effects on NMDAR gating that shared some similarities with GluN2A-P552R. The mutation GluN1-P557R caused an increase in glutamate and glycine potency when expressed with GluN2A or GluN2B that was similar to that observed for GluN2A-P552R. However, the rise time of receptors containing GluN1-P557R was identical to that observed in WT receptors, an effect that is distinct from the prominent slowing of the rise time observed for GluN2A-P552R. This difference implies that the pre-M1 region of GluN1 and GluN2 contribute to channel gating by unique mechanisms, suggesting different roles of these two subunits in gating. These results are consistent with the distinct structural movement of the GluN1 and GluN2 linkers as NMDARs open (see Figs 5–6 in [47]), as well as the hypothesis that conformational changes in the GluN2 subunit may be rate-limiting [48] (but see Kussius and Popescu [49]). The diminished genetic selection of the GluN1 pre-M1 cuff helix compared to strong purifying selection of the GluN2 pre-M1 helix (Fig 1) also supports functional differences in the contribution of GluN1 *vs* GluN2 to channel gating.

The amino acid exchange from Pro to Arg has several potentially important actions. Evaluation of the effect of other residues introduced in place of this proline provides some insight into the potential nature of the actions of the Pro552Arg mutation. Only substitution of a Lys residue, predicted to possess a positive charge similar to Arg at physiological pH, could mimic the effects of GluN2A-P552R, suggesting the charge of this side chain is more important than either chain flexibility or steric considerations. Interestingly, introduction of Gly, which lacks a side chain, increased the speed of activation, supporting a key role for the pre-M1 helix in the steps leading to channel activation. By contrast, introduction of a leucine residue with a similar side chain size diminishes response amplitude and significantly increases desensitization, yet was without effect on response rise time or deactivation time course. Interestingly, the patient with the GluN2B-P553L mutation shows substantial intellectual disability, consistent with the idea that normal NMDAR function plays a key role in learning and memory [4]. The reduced function of this receptor also might disrupt activity-dependent synapse formation and maturation [50], which could contribute to developmental delay and intellectual disability. Other loss of function mutations in this region (Table 3) (Fig 3) may have similar effects.

Several lines of evidence suggest that the pre-M1 helix may be involved in the process of opening of the channel pore. First, mutations of individual residues within this region to cysteine produce receptors with small currents with abnormal kinetics [51–53]. Second, MTS modification of cysteine residues introduced into this region produced significant changes in leak current [54]. Third, mutation of select pre-M1 residues to alanine can produce spontaneously active receptors [55]. Fourth, the pre-M1 helix and adjacent regions harbor structural determinants for subunit-selective positive allosteric modulators of the GluN2C- and GluN2D-containing NMDARs [56,57]. Fifth, mutations in and near pre-M1 can alter NMDAR function [14,29]. The position of the pre-M1 helix in the closed AMPAR and NMDAR structures [10,12,18] evokes the hypothesis that these helices, in van der Waals contact with the M3 gate, may serve to stabilize the gate in the closed conformation or otherwise impede M3 movement during channel opening. If this is correct, closure of the bi-lobed agonist binding domains could allow the conformation of the linker connecting the lower lobe of the clamshell to the pre-M1 helix to change, and likely alter the position or conformation of the pre-M1 helix. Conformational changes in the linker and pre-M1 helix subsequent to clamshell closure around the agonist could range from subtle (e.g. slight repositioning of a single amino acid side chain) to more pronounced (e.g. rotation or displacement of the pre-M1 helix). Following this idea, we hypothesize that rapid all-or-none pore dilation occurs when a subset of the pre-M1 helices have undergone such independent conformational changes, which together reduce the energy needed for the pore to adopt an open conformation, free from the constraints imposed by the pre-M1 cuff helices.

**Fig 9:**
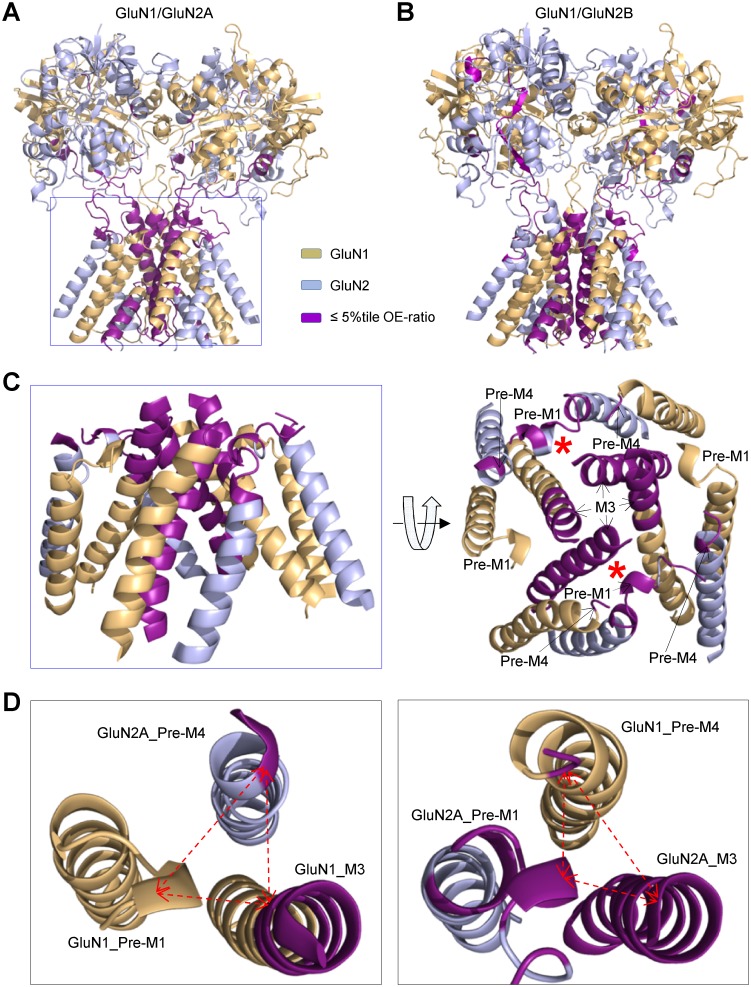
Potential interaction between the pre-M1 and M3 helices. **A**,**B**, Ribbon structures of the GluN1/GluN2A (***A***) and GluN1/GluN2B (***B***) receptors without the amino terminal domain is shown. GluN1 is tan and GluN2 is light blue; regions with an OE-ratio below the 5^th^ percentile are colored purple, and indicate the regions under the strongest purifying selection. **C**, Side and top down view of the pore forming elements M1, M3, M4 in GluN1/GluN2A receptors colored as in (***A***), with regions of purifying selection shown in purple. **D**, Expanded view of the pre-M1 helix for GluN1 (*left panel*) and for GluN2A (*right panel*).

Fig 9A and 9B maps the elements that are under the strongest purifying selection onto the structure of the agonist binding domain and channel pore. Interestingly, in the agonist binding domain, the regions of GluN1 S2 connecting with pre-M4 helix and GluN2B S1 connecting with pre-M1 helix show a strong purifying selection (S8 Fig). Furthermore, the regions within the ion channel pore that are under strongest selection are in close proximity (Fig 9C and 9D); the GluN2 pre-M1 helix / M3 helix and GluN1 pre-M4 region all reside within ~5 Angstroms, and this close arrangement of interacting amino acids could comprise a key gating element. The rearrangement of the position of constituent interacting side chains within this region may be a critical step in channel opening. We suggest that independent, non-sequential changes in the conformation of each pre-M1 helix or linker could constitute the rate limiting steps preceding channel opening, and the rates at which these conformational changes occur might establish two or more kinetically distinct closed channel conformations detectable in single channel recordings [48,58]. An alternative hypothesis that each pre-M1 helix contributes to the activation energy for simultaneous pre-M1 movement and pore dilation can also accommodate differential or asymmetrical contributions of GluN1 and GluN2 subunits to gating, provided each subunit contributes a different portion of the activation energy required for opening.

The different effects of the Pro-Arg substitution in GluN1 *vs* GluN2 response time course are consistent with subunit-specific conformational changes that precede opening of the channel pore [11,48,59,60]. In such a scenario, mutations that stabilize the pre-M1 helix in the channel closed configuration should slow channel opening, but not necessarily agonist binding. Introduction of the Arg residue may enable an additional hydrogen bond or ionic interaction that stabilizes the closed conformation. Interestingly, a slowing is only observed for NMDARs that contain two copies of the mutant GluN2A-P552R subunit. When a single mutant copy of GluN2A-P552R is present in the receptor complex, the channel opens at the same rate, suggesting that perhaps only one of the GluN2A subunits needs to have the pre-M1 free to undergo a conformational change following agonist binding. Mutations introduced into GluN1 do not detectably alter response rise times, consistent with the idea that pre-requisite conformational changes in GluN1 are faster than those in GluN2, not rate limiting, and distinct from those in GluN2.

Two additional properties of the channel are also impacted only when two mutant GluN2 subunits are present. Inclusion of 2 copies of GluN2A-P552R in the receptor modestly reduces single channel conductance and prolongs the mean channel open time. One potential interpretation of this observation is that the M3 domain retains contact with the pre-M1 region even after the channel opens, since a change in conductance must reflect changes in the shape or electrostatic environment of the ion channel pore or access portals that are induced by the pre-M1 helix harboring mutant GluN2A-P552R. When only a single mutation is present in the receptor complex, this contact has minimal effect and the channel can open and conduct ions at the same rate as under normal circumstances. When two mutations are present, there is more substantial interaction between the pre-M1 region and the M3 helices (or other gating elements), which leads to altered conductance and open time. These results provide new insight into the steps that lead to NMDAR opening.

There is a critical need to provide functional assessment of the rapidly growing number of disease-associated rare variants in *GRIN* and other gene families. It is not possible to predict the effects of, for example, the Pro-Arg exchange at position 552 in GluN2A (or other *de novo* pre-M1 mutations) on NMDAR currents without functional data, nor is it possible to conceptualize hypotheses about how missense mutations might impact circuit function or neuronal health without knowing how they change receptor properties. The functional assessment of GluN2A-P552R described here provides clear hypotheses about how this mutation might influence human health, and provides a rationale for study of its impact on neuronal function. The changes in synaptic function and acute neurotoxicity observed in cultured neurons transfected with mutant NMDAR cDNA raise the possibility that parenchymal cell loss observed with epileptic encephalopathy in patients with *GRIN* gain-of-function mutations may reflect excitotoxicity secondary to NMDAR overactivation during both seizures and at rest. We note that in the heterologous expression systems employed here, there is no detectable change in surface expression or response amplitude, suggesting that the changes in time course we observe and increased charge transfer drive dendritic swelling and toxicity in neurons. However, an important caveat to bear in mind when interpreting the overexpression studies described here is the unknown contribution of native receptors *vs* overexpressed receptors at the synapse. We expect that some of the synaptic response will be mediated by receptors that contain one or more WT GluN2 subunits, consistent with the observation that the change in time course observed in neurons transfected with GluN2A-P552R was smaller than that predicted by the prolongation of the response to brief application of glutamate. While these acute experiments do not account for potential homeostatic mechanisms *in vivo* that may be neuroprotective, they nevertheless raise the possibility that inhibition of mutant NMDAR subunits with properties similar to GluN2A-P552R might reduce abnormal excitatory drive in patients, and be neuroprotective, preserving grey matter and perhaps cognitive function if implemented at an early age.

## Materials and Methods

All animal work in this study was conducted according to the guidelines of Emory University, the University of Pittsburgh, and Harvard University. All procedures involving the use of animals were reviewed and approved by the University of Pittsburgh IACUC and the Harvard University IACUC. All reagents were purchased from Sigma Aldrich (St. Louis, MO, USA) unless otherwise noted.

### Identifying where purifying selection has acted strongest within *GRIN* genes

To illustrate the missense variant intolerance landscape of *GRIN1*, *GRIN2A* and *GRIN2B* we use aggregated variant information from a recently available collection of 141,352 unrelated individuals, the gnomAD data set (beta release accessed November 2016; [28]). We focused on the protein-coding missense and synonymous single nucleotide variants (SNVs) reported in the following isoforms:

*GRIN1*, NM_007327.3, CCDS7031.1, ENST00000371561 (Uniprot Q05586-1).

*GRIN2A*, NM_000833.4, CCDS10539.1, ENST00000396573 (Uniprot Q12879-1).

*GRIN2B*, NM_000834.3, CCDS8662.1, ENST00000609686 (Uniprot Q13224-1).

We restricted the gnomAD data to variants that were judged to “PASS” the gnomAD quality criteria. This resulted in 166 distinct missense and 259 distinct synonymous *GRIN1* variants; 628 distinct missense and 422 distinct synonymous *GRIN2A* variants; 429 distinct missense and 428 distinct synonymous *GRIN2B* variants (http://gnomad.broadinstitute.org/; accessed 8^th^ November 2016).

We used a 31 codon (93 nucleotide) sliding window to determine the proportion of observed missense variants reported in gnomAD given the sum of observed missense and synonymous variants in that window. A sliding window approach is adopted to allow our estimates to be agnostic to any existing biological boundaries, such as exons, conserved domains, functional domains, etc. The sliding window contains information about the 15 residues before and 15 residues after the residue of interest. The first and last 15 residues reflect smaller window sizes. To calculate the expected proportion of missense variants in that same window we simulated all possible variants in the window and annotated those simulated variants with Ve!P (Ensembl GRCh37 release 85—July 2016), based on the isoforms defined above.

We then divided the observed proportion of gnomAD missense variants in a window by the expected proportion of missense variants based on the underlying sequence context of that window to give us a regional Observed proportion / Expected proportion ratio (OE-ratio). We used this sliding window OE-ratio to illustrate regional intolerance of missense variants (black line in Fig 1). An OE-ratio of 1 (blue line in Fig 1) reflects windows where we observe the same proportion of missense variants as we would expect. A decreasing OE-ratio corresponds with a greater intolerance to missense variation in that window as reflected by a greater proportion of synonymous variation. We find that the median OE-ratio estimate for each of the three genes is considerably lower than 1, which is the median we would expect to be close to if these genes had not been under purifying selection. The low median OE-ratio for these three genes is what we expect given our previous gene-based residual variation intolerance score (RVIS) highlights *GRIN1* (RVIS = -1.1; 6.7%tile), *GRIN2A* (RVIS = -1.5; 3.9%tile) and *GRIN2B* (RVIS = -2.4; 1.1%tile) as being among the most overall intolerant genes in the exome [1]. Here, like has been introduced previously [27], we take a step forward and identify the regions within these three genes that have been under the strongest purifying selection in the human lineage.

Given the beta version status of the gnomAD data, we have also generated these OE-ratio sliding window plots using the established ExAC data set of 60,706 unrelated individuals (release 0.3.1 accessed October 2015) as S6 Fig.

### Analysis of genetic variation on S1-M1 linker in *GRIN1*, *GRIN2A*, and *GRIN2B*

Literature-based patient-ascertained *de novo* missense variants were identified through two database searches and a review of recent literature. First, a search for “pathogenic” missense variants in ClinVar (accessed May 2016) was performed. Next, a search for DM-classified variants was performed based on HGMD (hgmd2016.1). All source papers for the ClinVar and HGMD pathogenic-reported missense variants were evaluated and only missense variants where the source papers specified that the variants arose *de novo* in the patient were retained. Finally, two recent large-scale trio sequencing studies in developmental delay (http://biorxiv.org/content/early/2016/06/16/049056) and intellectual disability (http://biorxiv.org/content/early/2016/05/11/052670) were also screened for reported *de novo* mutations in the gene of interest.

For *GRIN1* this screen identified two ClinVar-based pathogenic reported *de novo* variants, an additional seven distinct HGMD-based pathogenic reported *de novo* variants and three distinct patient-ascertained missense *de novo* variants from the two trio sequencing papers. For *GRIN2A* this screen identified four ClinVar-based pathogenic reported *de novo* variants, an additional six distinct HGMD-based pathogenic *de novo* reported variants and three distinct patient-ascertained missense *de novo* variants from the two trio sequencing papers. For *GRIN2B* this screen identified eight ClinVar-based pathogenic reported *de novo* variants, an additional four distinct HGMD-based pathogenic reported *de novo* variants and nine distinct patient-ascertained missense *de novo* variants from the two trio sequencing papers. A full list of the resulting 46 literature-based missense *de novo* mutations among *GRIN1*, *GRIN2A* and *GRIN2B* are reported in **Supplemental** S1 Table.

### Mutagenesis

The GenBank accession numbers for rat cDNAs were U08261 for GluN1-1a lacking exon5 but containing exon21 and 22 (hereafter GluN1), D13211 for GluN2A, M91563 for GluN2C, and L31611 for GluN2D. Human cDNAs encoding GluN1, GluN2A, GluN2B, and GluN2D were used for experiments involving diheteromeric mutant or WT GluN1/GluN2 NMDARs. The full open reading frames of human NMDAR subunits were assembled from cDNA fragments obtained from the I.M.A.G.E. Consortium (Carlsbad, CA, USA) and Origene (Rockville, MD), as previously described [61]. We introduced point mutations into NMDAR cDNAs using Quikchange reactions (Agilent Technologies). The C1 and C2 peptide tags that were used to control rat GluN1/GluN2A NMDAR composition were previously described [32,33]. cDNAs for recombinant NMDAR subunits were subcloned into the pCI-neo mammalian expression vector. For expression in *Xenopus* oocytes, DNA constructs were linearized by restriction enzymes and used as templates for *in vitro* cRNA transcription using the mMessage mMachine kit (Ambion, Austin, TX, USA).

### Two electrode voltage clamp recordings from *Xenopus* oocytes

Healthy-looking, defolliculated stage V-VI *Xenopus laevis* oocytes (EcoCyte Bioscience) were injected with cRNA for GluN1 and GluN2 in a 1:2 ratio (5–10 ng total in 20–50 nl water). Experiments utilized human GluN1/GluN2A and GluN1/GluN2B, rat GluN1/GluN2A_C1_/GluN2A_C2_ [33], and rat GluN1/GluN2C and GluN1/GluN2D cRNA. Following injection, oocytes were maintained at 15°C in Barth’s culture medium containing (in mM) 88 NaCl, 2.4 NaHCO_3_, 1 KCl, 0.33 Ca(NO_3_)_2_, 0.41 CaCl_2_, 0.82 MgSO_4_, 5 Tris-HCl (pH 7.4 with NaOH), 1 U/mL penicillin, 0.1 mg/mL gentamicin sulfate, and 1 μg/mL streptomycin. Recordings were performed 2–7 days after injection at 23°C. Oocytes were transferred to a recording chamber and continuously perfused with extracellular recording solution containing (in mM) 90 NaCl, 1 KCl, 10 HEPES, 0.5–1 BaCl_2_, 0.01 EDTA and brought to pH 7.4 with NaOH. Microelectrodes were fabricated from borosilicate glass (World Precision Instruments catalog no. TW150F-4; Sarasota, FL, USA) and voltage clamp and current monitoring were achieved with a two-electrode voltage clamp amplifier (Warner Instruments model OC-725C; Hamden, CT, USA). Currents were low-pass filtered at 10 Hz and digitized at 20 Hz using custom acquisition software written in LabWindows/CVI (National Instruments, Austin, TX, USA), which also controlled solution exchange. Oocytes were held at -40 mV unless otherwise stated. The NMDAR open probability was estimated as previously described using MTSEA to lock open NMDA receptors expressing mutant GluN1(A652C)[62]. Open probability was calculated as (γ_MTSEA_/γ_CONTROL_)/*potentiation* where γ_MTSEA_ and γ_CONTROL_ are chord conductances estimated from control and MTSEA-modified GluN1/GluN2A receptors [62] and *potentiation* is the ratio of current observed after MTSEA modification to control.

### Voltage clamp recordings from transfected HEK293 cells

HEK293 cells (ATCC CRL-1573) were plated on glass coverslips coated with poly-D-lysine (10–100 ug/ml) and were transiently transfected using the calcium phosphate precipitation method) with plasmid cDNAs encoding WT or mutant human GluN1/GluN2A, human GluN1/GluN2B, rat GluN1/GluN2C, rat GluN1/GluN2D; GFP was co-transfected to allow identification of cells expressing recombinant receptors (0.2 g/L total cDNA)[63]. The ratio for GluN1:GluN2:GFP cDNA was 1:1:1. For some experiments rat GluN1/GluN2A_C1_/GluN2A_C2_/GFP were co-transfected at equal ratios into HEK293 Tet-On Advance cells (Clontech Laboratories, Mountain View, CA, USA). 200 μM AP-5 and 200 μM 7-chlorokynuernic acid were added to the culture media to prevent excessive activation of the NMDARs and reduce cell death. Cells were used for recordings 18–24 hours following transfection. For recordings, cells were transferred to a recording chamber and continuously perfused at 2 mL/min with recording solution containing (in mM) 150 NaCl, 3 KCl, 10 HEPES, 0.01 EDTA, 0.5 CaCl_2_, and 11 D-mannitol, with the pH adjusted to 7.4 by addition of NaOH. Microelectrodes were fabricated using thin-walled filamented borosilicate glass (World Precision Instruments catalog TW150F-4; Sarasota, FL, USA) pulled using a vertical puller (Narishige P-10, Tokyo, Japan). The internal pipette solution contained (in mM) 110 D-gluconic acid, 110 CsOH, 30 CsCl, 5 HEPES, 4 NaCl, 0.5 CaCl_2_, 2 MgCl_2_, 5 BAPTA, 2 Na_2_ATP, 0.3 NaGTP adjusted to pH 7.35 with CsOH; the osmolality was adjusted to 300–310 mOsmol/kg using CsCl or water. Pipette tips were filled with internal solution and had resistances of 3–4 MΩ when placed into recording solution. The membrane potential of HEK293 cells was held at -60 mV using an Axopatch 200B patch-clamp amplifier (Molecular Devices, Sunnyvale, CA, USA) and current responses to external application of glutamate (100 μM) and glycine (30-100 μM) were recorded at 23°C and anti-alias filtered at 8 kHz (-3 dB, 8 pole Bessel filter, Frequency Devices, Ottawa, IL, USA) and digitized at 20 kHz using a Digidata 1440A data acquisition system (Molecular Devices) controlled by Clampex 10.3 (Molecular Devices). The current response time course was fitted to the sum of exponential 1 or 2 functions using non-linear least squares algorithms. Charge transfer was estimated as the product of peak amplitude and the weighted deactivation time course divided by the cell capacitance for responses to 5 ms application of glutamate.

### Single channel recordings

Excised outside-out patches were obtained from HEK293 Tet-On cells transiently transfected with rat GluN1, GluN2A_C1_, GluN2A_C2_, and GFP as described above for whole cell recordings. GluN2_C1_ and GluN2_C2_ were either WT or contained the Pro552Arg mutation. The recording solution was the same as in whole-cell recordings, except the pH was adjusted to 8.0. For outside-out patch recordings, micropipettes were fabricated from filamented thick-walled borosilicate glass (Warner Instruments cat. no. GC150F-10; Hamden, CT, USA). Electrodes were pulled using a Flaming/Brown horizontal puller (Sutter Instrument model no. P-1000, Novato, CA, USA), and coated with Sylgard silicone elastomer (DuPont, Santa Barbara, CA, USA). Pipette tips were fire-polished as in whole-cell recordings. For outside-out recordings, pipette tips were filled with the same internal solution as in whole-cell recordings. Tip resistances for outside-out patches were 7–9 MΩ. 1 mM glutamate and 50 μM glycine were used to activate NMDARs in outside-out patch recordings. The holding potential was -80 mV for outside-out patches recorded at 23°C. Currents were anti-aliased low pass filtered at 8 kHz (-3 dB Bessel 8-pole; Frequency Devices, Ottawa, IL, USA) and digitized at 40 kHz.

Digitized records of single channel currents were loaded in QuB Classic software (Milescu, L.S., Nicolai, C., Bannen, J., 2000–2013 QuB Software) and converted into QuB Data Format, joining all segments together. The baseline was corrected by setting a period of the recording with no openings as a baseline and adding baseline nodes throughout the recording. Stretches of openings with no obvious double openings were selected for analysis of amplitudes and channel open times. Single channel openings were idealized using the segmental K-means (SKM) algorithm in QuB [64] with the channel gating mechanism of Model 16 in Auerbach and Zhou [58]. The dead time was set to 0.05 ms and initial amplitudes were estimated using the "grab all amps" function of QuB. After an initial SKM idealization, rates in the gating mechanism were refined using the maximum interval likelihood (MIL) estimation algorithm in QuB [65,66]. Openings were then re-idealized with the refined rates using the SKM algorithm. Idealization statistics reported by QuB were used for statistical analysis.

Average channel amplitudes of the open state and closed stated were estimated as part of the SKM idealization algorithm in QuB. The average amplitudes of the open state and shut state were used for analysis (and not the re-estimated amplitudes). For statistical analysis, open channel amplitudes were calculated for each patch as the difference between the open state amplitude and closed state amplitude, and the channel chord conductance was calculated by dividing the open channel amplitude by the holding potential, assuming a reversal potential of 0 mV. Visual inspection of open time histograms suggested that open times were a mixture of two exponential components. The maximum likelihood estimates for the means of the two fitted exponential components and their corresponding weights were determined for each patch using custom MATLAB functions (available at https://github.com/ogdenkev).

### Optical assay for synaptic transmission

Neurons were harvested from P0 rats and cultured on glial monolayers in NBActiv4 (Brainbits, Springfield, IL, USA) and fed every 4 days. Neurons were electroporated with cDNA encoding either GFP-GluN2B, which has GFP fused in frame to the N-terminal, or GFP-GluN2B-P553R, leading to sparse expression of the NMDARs. We used Cre-dependent constructs to achieve mutually exclusive expression of a channelrhodopsin variant, CheRiff-mOrange, and a genetically encoded voltage indicator, QuasAr in disjoint subsets of primary rat hippocampal neurons. Cells were infected with two lentiviruses containing CAG::Cre-on CheRiff and hSyn::Cre-off QuasAr. Low titer AAV containing hSyn::Cre activated the CheRiff and inactivated the QuasAr in a subset of the neurons. Imaging was performed at 14–19 days *in vitro* at room temperature (23°C) in extracellular medium comprising of (in mM) 125 NaCl, 2.5 KCl, 3 CaCl_2_, 1 MgCl_2_, 15 HEPES, 30 glucose, pH 7.3, and adjusted to 305–310 mOsm with sucrose. To isolate NMDAR-mediated potentials, data were acquired in 20 μM NBQX, 50 μM picrotoxin, and in the nominal absence of Mg^2+^. Cells co-expressing the voltage indicator and the GFP-GluN2B were visually identified. Presynaptic cells were stimulated with pulses of blue light to elicit single action potentials (488 nm, 1 Hz, 10 ms duration, typically 5 to 10 pulses). QuasAr fluorescence in the postsynaptic cell was excited with a red laser (635 nm), collected in the near infrared (667–740 nm), and imaged at a frame rate of 500 Hz. Fluorescence traces from the somas of postsynaptic cells were averaged across all pulses and then across cells. Absolute fluorescence values are not meaningful due to cell-to-cell variations in expression level of the voltage indicator, so responses were normalized and displayed on a scale from 0 to 1.

Quantification of the percentage of inhibitory neurons in the culture was performed via immunostaining with mouse anti Gad67 (Millipore MAB5406) and goat anti mouse 594 (Abcam ab150116). The immunostaining was following the protocol described in [43]. Inhibitory neurons were counted manually in fluorescence images. The total number of neurons was determined from transmitted light images. 72 out of 1358 neurons were Gad67^+^.

### Surface protein biotinylation and western blotting

HEK293 cells were transfected with human cDNA, and surface protein biotinylation was performed as described previously [67]. Briefly, 24 hours after transfection, cells were incubated with 1 mg/ml Sulfo-NHS-biotin for 20 min on ice, and then the biotin was quenched with 50 mM glycine. After cell lysis, protein concentrations were determined by the Bradford assay, and equal amounts of protein from each sample were added to Neutravidin beads (ThermoFisher) and rotated for 2 hr at 4°C. Total protein and pulled-down surface protein fractions were separated by SDS-PAGE, and immunoblotted using the following antibodies: mouse anti-GluN1 (BD Pharmingen, San Jose, CA, USA), rabbit anti-GluN2A (Millipore, AB1557), mouse anti-GluN2B (Alomone Labs, Jerusalem, Israel), mouse anti-transferrin receptor (Sigma), and mouse anti-tubulin. Transferrin receptor levels were used to assess whether biotin labeling was consistent across conditions. Tubulin levels were used as a loading control for total protein samples and to ensure only surface proteins were in biotinylated fractions. Chemiluminescence signals were detected with film, imaged with a Bio Rad Gel Imager, and quantified using ImageJ.

### Transfection, treatment, and luciferase viability assay

Mixed neuronal/glial cortical cultures were derived from E16/17 Sprague Dawley rats of either sex and maintained as previously described [68]. Neurons were transfected at 19–20 DIV utilizing Lipofectamine 2000 [69]. Cells were transfected with the following plasmid mixtures (total of 1.5 μg total DNA/well, corresponding to 2.5 μg DNA/ml): 25% pUHC13-3 Luciferase, 35% or 55% pEGFP-N1, and 20% or 40% of either pCIneo-vector, human WT GluN2A, or human GluN2A-P552R. Transfection efficiency was 6–10%. Half of the transfected cultures were treated with memantine (20 μM or 50 μM) until analysis. A luciferase viability assay was employed 48 h following transfection as previously described [44]. Viability was also confirmed by cell counts (see below). Each condition was normalized to its relevant vector-transfected group to obtain relative viability values expressed as % control for each experiment (i.e. WT GluNA, GluN2A-P552R + memantine was standardized to pCIneo + memantine).

### Confocal imaging and cell viability counts

In order to obtain standardized cell counts, cortical neuronal cultures on glass coverslips were imaged on a Nikon A1R microscope at 20× magnification following transfection and treatment (+ or—memantine) for 48 h. Coverslips were divided into four quadrants, and an image field was obtained from one randomized area in each quadrant. For each image field (649 μm^2^), between 10–20 optical sections (1.5–2.00 μm) were obtained to generate a maximum intensity projection image for analysis. For analysis, maximum intensity projection images (Max-IP) were used along with the object count feature in Nikon NIS-Elements Advanced Research software (Nikon Instruments Inc., NY, USA). Object count parameters were customized to define areas of intense GFP signal with a minimum 2D-cell surface area of 100 μm^2^, representative of neuronal cell bodies. Intensity thresholding was varied slightly based on differential GFP expression in neuronal cultures. Following automated counting, cell counts were manually verified to account for inclusion of non-cellular bodies (debris, autofluorescence, etc. or to separate adhered neurons that would have been counted as a single soma). In order to obtain a viability (% control) value for each experiment, cell count values from the four quadrants were averaged to yield an average # neurons/field for each coverslip. For experimental groups (WT GluN2A, GluN2A-P552R), these values were then standardized to relevant vector-transfected values (i.e. WTGluN2A + memantine was standardized to vector-transfected + memantine). This procedure was then replicated in six independent experiments to yield a mean viability (% control) value for each condition. For qualitative imaging to demonstrate dendritic swellings, cortical neuronal cultures on glass coverslips were imaged by similar procedure, either utilizing 20× or 40× magnification. The object count program was also utilized to detect bleb formation, as described in S5 Fig.

### Statistical analysis

Data were analyzed using OriginLab software v9.0. Data were tested for normality and homogeneity of variances with the Kolmogorov-Smirnov and Levene’s test, respectively. Significance level was set at 0.05. Current amplitudes (Fig 3A and 3B) (Table 3) were tested for differences against WT GluN1/2A or GluN1/2B using the Kruskal-Wallis test with follow-up Mann-Whitney U test. Differences in chord conductance and mean open time between receptors with 0, 1, or 2 copies of GluN2A-P552R were evaluated using a one-way ANOVA and pairwise comparisons between the receptors were assessed using Tukey's honest significant difference tests. Other parameters were assessed by ANOVA or t-test, as appropriate. Statistical tests on potency were performed on logEC_50_ and logIC_50_. Significance was set at *p* < 0.05. Specific statistical tests are stated in the figure legends.

## Supporting Information

S1 Fig Surface expression of pre-M1 mutations (related to Fig 3). The surface proteins of HEK293 cells transiently expressing wild type or mutated human NMDARs were labeled with biotin and pulled down with avidin-conjugated beads. The total and surface protein fractions were run on SDS-PAGE gels and immunoblotted for GluN1, GluN2A or GluN2B, transferrin receptor (TfR), and tubulin. Representative western blots are shown for HEK cells expressing GluN1/GluN2A and GluN1-D552E/GluN2A (A), GluN1/GluN2A and GluN1/GluN2A-A548T (B), GluN1/GluN2A and GluN1/GluN2A-P552R (C), GluN1/GluN2A and GluN1/GluN2A-P552L (D), GluN1/GluN2B and GluN1-D552E/GluN2B (E), and GluN1/GluN2B and GluN1/GluN2B-P553L (F).
(PDF)

S2 Fig Fitted time constants for GluN2A-P552R open time histograms (related to Fig 4). The open times for each patch were modelled as a mixture of two exponential components. The maximum likelihood estimates for the means of the two exponential components and their corresponding weights were determined for each patch. The top panel shows the estimated mean, tau, of the first exponential component and the bottom panel shows the estimated mean of the second component. The size of each point corresponds to the estimated area of that component, and points are colored by the receptor type.
(PDF)

S3 Fig Rescue pharmacology to evaluate the ability of NMDAR antagonists including FDA-approved drugs on inhibition of human NMDAR function (related to Fig 8 and Results). Rescue pharmacology to evaluate the ability of NMDAR antagonists including FDA-approved drugs on inhibition of human NMDAR function by using two-electrode voltage clamp current recordings (holding at -40 mV) on *Xenopus* oocytes. The data are expressed as IC_50_ value ± SEM (n, maximal inhibition % at 100 μM for memantine, 300 μM for dextromenthorphan, 30 μM for dextrorphan, 1000 μM for amantadine, 100 μM for ketamine, 10 μM for TCN-201).
(PDF)

S4 Fig Comparison of blebbing produced by transfection of neurons with GluN2A-P552R cDNA (related to Fig 8, S5 Fig, and Results). Additional experimental results are shown for neurons transfected with GluN2A-P552R. Morphological features of rat cortical neurons in culture (DIV 18–19) expressing GFP and either GluN2A WT (0.6 μ g; see Methods and Fig 8), or GluN2A-P552R (0.6 μ g) for 24 hours. Blebs are a telltale and nearly ubiquitous sign of neuronal expression of GluN2A-P552R, but not GluN2A WT. Panels are representative of 5 independent transfection experiments for each vector, not necessarily paired across rows. Scale bar = 100 μm.
(PDF)

S5 Fig Quantification of GluN2A-P552R induced blebbing in transfected neurons (related to Fig 8, S4 Fig, and Results). Quantification of blebbing in neurons transfected with GluN2A WT and GluN2A-P552R cDNA. Morphological features of rat cortical neurons in culture (DIV 18–19) expressing GFP and either empty vector, GluN2A WT (0.3 μ g; see Methods and Fig 8), or GluN2A-P552R (0.3 μ g) for 24 hours. Although very rarely some dendritic blebs are observed in WT GluN2A-expressing neurons (e.g. see third panel from the top, middle row), blebs are a telltale and nearly ubiquitous sign of neuronal expression of GluN2A-P552R. Panels are representative of 11 different fields obtained from three separate coverslips for each condition for one representative experiment. We utilized an unbiased object count program (NIS elements, Nikon) to obtain the total number of blebs per field. The mean intensity for each field was utilized to set the threshold intensity and all objects from 2–100 μm were counted (cell bodies were excluded). Circularity was set to 0.25, with 1 being a perfect circle. Although these parameters detected objects in vector-expressing cells, these were attributed to spines or intrinsic dendritic tortuosity. Vector: 29.8 ± 6.2 objects/field; GluN2A WT: 59.2 ± 11.8; GluN2A(P552R): 115.4 ± 12.5. No statistical difference was observed between vector and WT; significant differences were observed between vector and mutant, and WT and mutant (p<0.001, 0.01, respectively; ANOVA/Tukey). Please note that we used a different pinhole in the confocal microscope (1.7) than the one used in S4 Fig (1.2) to increase the signal-to-noise ratio, which aided in the quantification procedure. Scale bar = 100 μm.
(PDF)

S6 Fig OE-ratio calculated with data from ExAC (related to Methods, Fig 1, and Results). Sliding window OE-ratio estimates (black full line), neutrality expected OE-ratio estimates derived from ExAC server. A, GluN1, B, GluN2A and C, GluN2B sliding window OE-ratio estimates (black full line), neutrality expected OE-ratio estimates derived from ExAC server accessed April, 2016 (blue full line), median OE-ratio for the gene (dark grey dashed line), 25th percentile of OE-ratio (green dashed line), 5th percentile of OE-ratio (red dashed line). The proportion of the 60,706 ExAC samples that had at least 10-fold coverage to be able to call a variant at the residue (cyan dashed line). The asterisk shows the Pro552 in GluN2A and Pro 553 in GluN2B.
(PDF)

S7 Fig Simultaneous electrical and optical voltage recording from neurons (related to Fig 7 and Results). Simultaneous fluorescence and manual patch clamp measurements of an excitatory post-synaptic potential in a neuron expressing a QuasAr voltage indicator. Presynaptic CheRiff-expressing neurons were stimulated with a single flash of blue light at the start of the measurement. See Lou et al (2016) for current recording methods.
(PDF)

S8 Fig Regional purifying selection of GluN1/GluN2B (related to Fig 9 and Discussion). Left panel shows a ribbon structure of the GluN1/GluN2B receptors without the amino terminal domain. GluN1 is tan and GluN2B is light blue; regions with purple color reflect an OE-ratio below the 5th percentile, indicating the regions under the strongest purifying selection. Side view of agonist binding domain (ABD) in an expanded panel (right) shows a strong purifying selection (in purple) in regions of GluN1 S2 connecting with pre-M4 helix and GluN2B S1 connecting with pre-M1 helix.
(PDF)

S1 Table Patient ascertained *de novo* mutations (related to Table 1). (PDF)

S2 Table Glycine deactivation time course for GluN1-P557R and GluN2A-P552R (related to Fig 4). (PDF)

S3 Table Sensitivity of GluN2A-P552R to endogenous negative modulators (related to Results). (PDF)

S4 Table Statistical analysis for data in Table 3. (PDF)

S5 Table Statistical analysis for data in Table 4. (PDF)

S6 Table Statistical analyses for S2 Table. (PDF)

S7 Table Statistical analysis for Table 6. (PDF)

S8 Table Statistical analysis for Table 7. (PDF)

S9 Table Statistical Data for Fig 8. (PDF)
