# Supplementary material for: Molecular Mechanism of Disease-Associated Mutations in the Pre-M1 Helix of NMDA Receptors and Potential Rescue Pharmacology
Source: PLoS Genet. 2017 Jan 17;13(1):e1006536. doi: 10.1371/journal.pgen.1006536 (PMC5240934; doi:10.1371/journal.pgen.1006536)
Supplement: S1 Fig — The surface proteins of HEK293 cells transiently expressing wild type or mutated human NMDARs were labeled with biotin and pulled down with avidin-conjugated beads. The total and surface protein fractions were run on SDS-PAGE gels and immunoblotted for GluN1, GluN2A or GluN2B, transferrin receptor (TfR), and tubulin. Representative western blots are shown for HEK cells expressing GluN1/GluN2A and GluN1-D552E/GluN2A (A), GluN1/GluN2A and GluN1/GluN2A-A548T (B), GluN1/GluN2A and GluN1/GluN2A-P552R (C), GluN1/GluN2A and GluN1/GluN2A-P552L (D), GluN1/GluN2B and GluN1-D552E/GluN2B (E), and GluN1/GluN2B and GluN1/GluN2B-P553L (F). (PDF) [file pgen.1006536.s001.pdf]

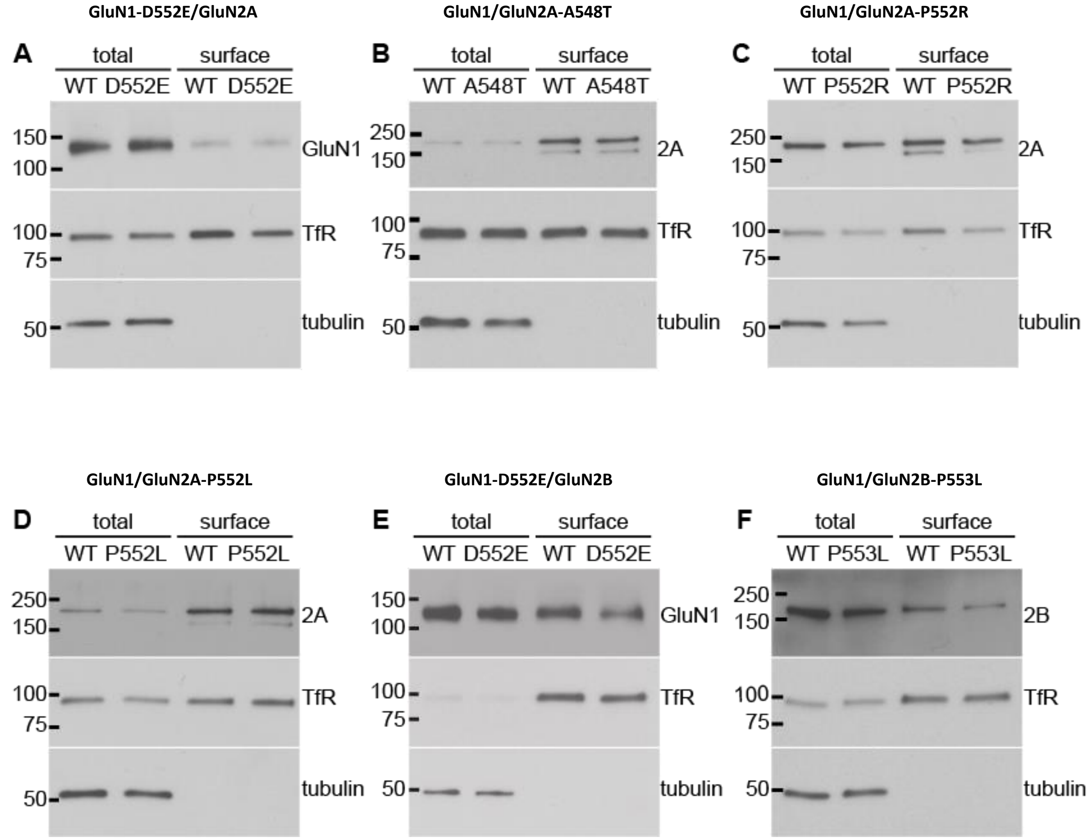

S1 Figure. Surface expression of pre-M1 mutations (related to Figure-3) The surface proteins of HEK293 cells transiently expressing wild type or mutated human NMDA receptors were labeled with biotin and pulled down with avidin-conjugated beads. The total and surface protein fractions were run on SDS-PAGE gels and immunoblotted for GluN1, GluN2A or GluN2B, transferrin receptor (TfR), and tubulin. Representative western blots are shown for HEK cells expressing GluN1/GluN2A and GluN1-D552E/GluN2A (**A**), GluN1/GluN2A and GluN1/GluN2A-A548T (**B**), GluN1/GluN2A and GluN1/GluN2A-P552R (**C**), GluN1/GluN2A and GluN1/GluN2A-P552L (**D**), GluN1/GluN2B and GluN1-D552E/GluN2B (**E**), and GluN1/GluN2B and GluN1/GluN2B-P553L (**F**).
