# Supplementary material for: Molecular Mechanism of Disease-Associated Mutations in the Pre-M1 Helix of NMDA Receptors and Potential Rescue Pharmacology
Source: PLoS Genet. 2017 Jan 17;13(1):e1006536. doi: 10.1371/journal.pgen.1006536 (PMC5240934; doi:10.1371/journal.pgen.1006536)
Supplement: S2 Fig — The open times for each patch were modelled as a mixture of two exponential components. The maximum likelihood estimates for the means of the two exponential components and their corresponding weights were determined for each patch. The top panel shows the estimated mean, tau, of the first exponential component and the bottom panel shows the estimated mean of the second component. The size of each point corresponds to the estimated area of that component, and points are colored by the receptor type. (PDF) [file pgen.1006536.s002.pdf]

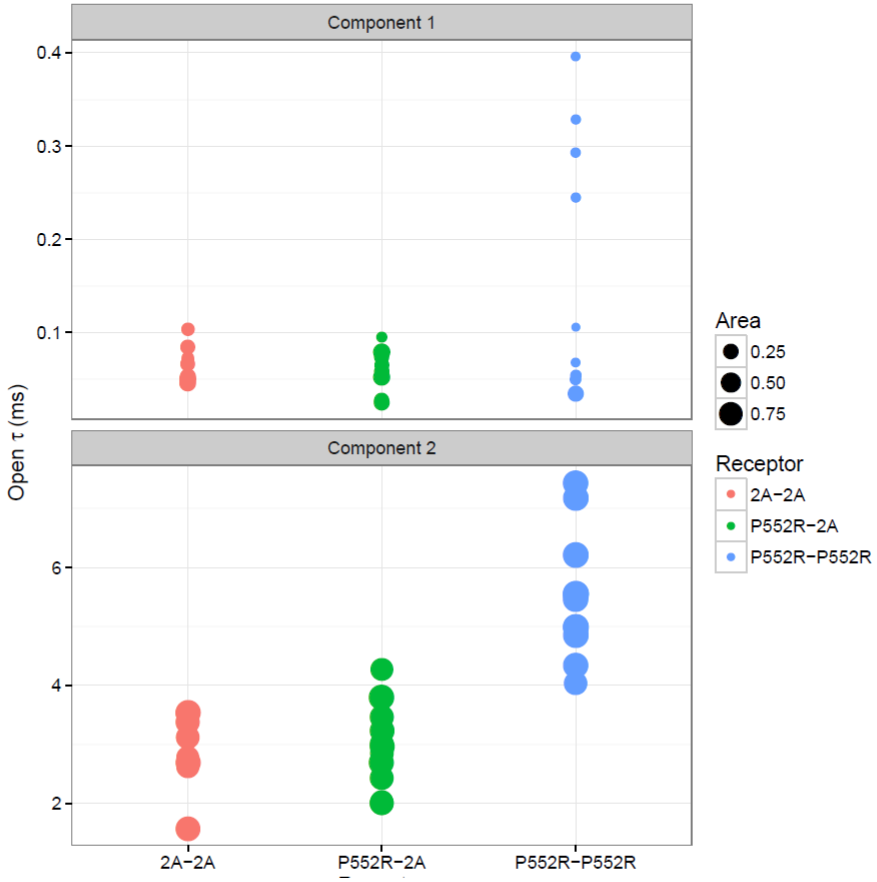

S2 Figure. Fitted time constants for GluN2A-P552R open time histograms (related to Figure-4) The open times for each patch were modelled as a mixture of two exponential components. The maximum likelihood estimates for the means of the two exponential components and their corresponding weights were determined for each patch. The top panel shows the estimated mean, tau, of the first exponential component and the bottom panel shows the estimated mean of the second component. The size of each point corresponds to the estimated area of that component, and points are colored by the receptor type.
