# Supplementary material for: Molecular Mechanism of Disease-Associated Mutations in the Pre-M1 Helix of NMDA Receptors and Potential Rescue Pharmacology
Source: PLoS Genet. 2017 Jan 17;13(1):e1006536. doi: 10.1371/journal.pgen.1006536 (PMC5240934; doi:10.1371/journal.pgen.1006536)
Supplement: S3 Fig — Rescue pharmacology to evaluate the ability of NMDAR antagonists including FDA-approved drugs on inhibition of human NMDAR function by using two-electrode voltage clamp current recordings (holding at -40 mV) on Xenopus oocytes. The data are expressed as IC50 value ± SEM (n, maximal inhibition % at 100 μM for memantine, 300 μM for dextromenthorphan, 30 μM for dextrorphan, 1000 μM for amantadine, 100 μM for ketamine, 10 μM for TCN-201). (PDF) [file pgen.1006536.s003.pdf]

Supporting Information

Molecular Mechanism of Disease-Associated Mutations in the Pre-M1 Helix of NMDA Receptors and Potential Rescue Pharmacology

Kevin K. Ogden[1,7], Wenjuan Chen[1,5,7], Sharon A. Swanger[1], Miranda J. McDaniel[1], Linlin Z. Fan[3], Chun Hu[1], Anel Tankovic[1], Hirofumi Kusumoto[1], Gabrielle J. Kosobucki[2], Anthony J. Schulien[2], Zhuocheng Su[1], Joseph Pecha[1], Subhrajit Bhattacharya[1], Slavé Petrovski[4], Adam E. Cohen[3], Elias Aizenman[2], Stephen F. Traynelis[1,6]*, Hongjie Yuan[1,6]*

[1]Department of Pharmacology, Emory University School of Medicine, Atlanta, GA 30322
[2]Department of Neurobiology, University of Pittsburgh School of Medicine and Pittsburgh Institute for Neurodegenerative Diseases, Pittsburgh PA 15217
[3]Department of Chemistry and Chemical Biology, Howard Hughes Medical Institute, Harvard University, Cambridge, MA 02138
[4]Department of Medicine, The University of Melbourne, Austin Health and Royal Melbourne Hospital, Melbourne, VIC, Australia
[5]Department of Neurology, Xiangya Hospital, Central South University, Changsha, 410013, China
[6]Center for Functional Evaluation of Rare Variants (CFERV), Emory University School of Medicine, Rollins Research Center, 1510 Clifton Road, Atlanta, GA 30322

Supporting Information

S1 Figure. Surface expression of pre-M1 mutations (related to **Figure-3**).

S2 Figure. Fitted time constants for GluN2A-P552R open time histograms (related to **Figure-4**).

S3 Figure. Rescue pharmacology to evaluate the ability of NMDAR antagonists including FDA-approved drugs on inhibition of human NMDAR function (related to **Figure-8** and **RESULTS**).

S4 Figure. Comparison of blebbing produced by transfection of neurons with GluN2A-P552R cDNA (related to **Figure-8, S5 Fig,** and **RESULTS**).

S5 Figure. Quantification of GluN2A-P552R induced blebbing in transfected neurons (related to **Figure-8, S4 Fig,** and **RESULTS**).

S6 Figure. OE-ratio calculated from ExAC (related to **METHODS**, **Figure-1,** and **RESULTS**).

S7 Figure. Simultaneous electrical and optical voltage recording form neurons (related to **Figure-7** and **RESULTS**).

S8 Figure. Regional purifying selection of GluN1/GluN2B (related to **Figure-9** and **Discussion**).

S1 Table. Patient ascertained de novo mutations (related to **Table-1**)

S2 Table. Glycine deactivation time course for GluN1-P557R and GluN2A-P552R (related to **Figure-4**)

S3 Table. Sensitivity of GluN2A-P552R to endogenous negative modulators (related to **RESULTS**)

S4 Table. Statistical analysis for data in **Table-3**.

S5 Table. Statistical analysis for data in **Table-4**.

S6 Table. Statistical analyses for **S2 Table**.

S7 Table. Statistical analysis for **Table-6**.

S8 Table. Statistical analysis for **Table-7**.

S9 Table. Statistical Data for **Figure-8**.

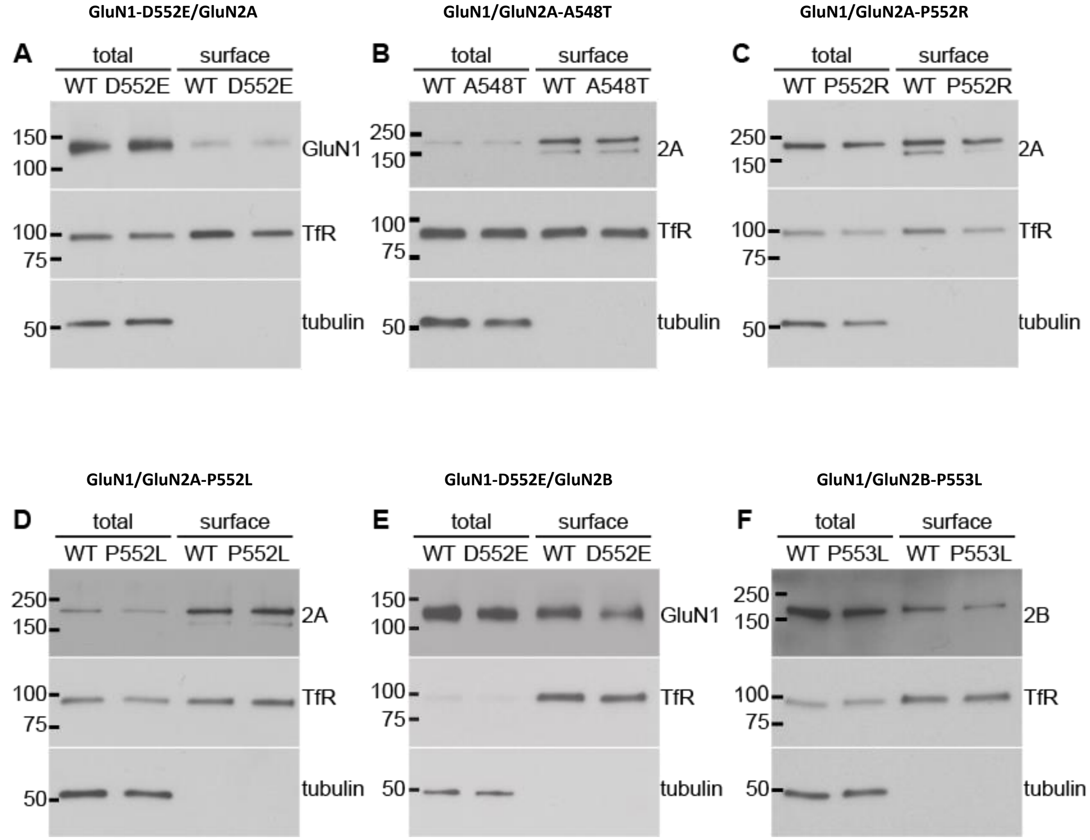

S1 Figure. Surface expression of pre-M1 mutations (related to Figure-3) The surface proteins of HEK293 cells transiently expressing wild type or mutated human NMDA receptors were labeled with biotin and pulled down with avidin-conjugated beads. The total and surface protein fractions were run on SDS-PAGE gels and immunoblotted for GluN1, GluN2A or GluN2B, transferrin receptor (TfR), and tubulin. Representative western blots are shown for HEK cells expressing GluN1/GluN2A and GluN1-D552E/GluN2A (**A**), GluN1/GluN2A and GluN1/GluN2A-A548T (**B**), GluN1/GluN2A and GluN1/GluN2A-P552R (**C**), GluN1/GluN2A and GluN1/GluN2A-P552L (**D**), GluN1/GluN2B and GluN1-D552E/GluN2B (**E**), and GluN1/GluN2B and GluN1/GluN2B-P553L (**F**).

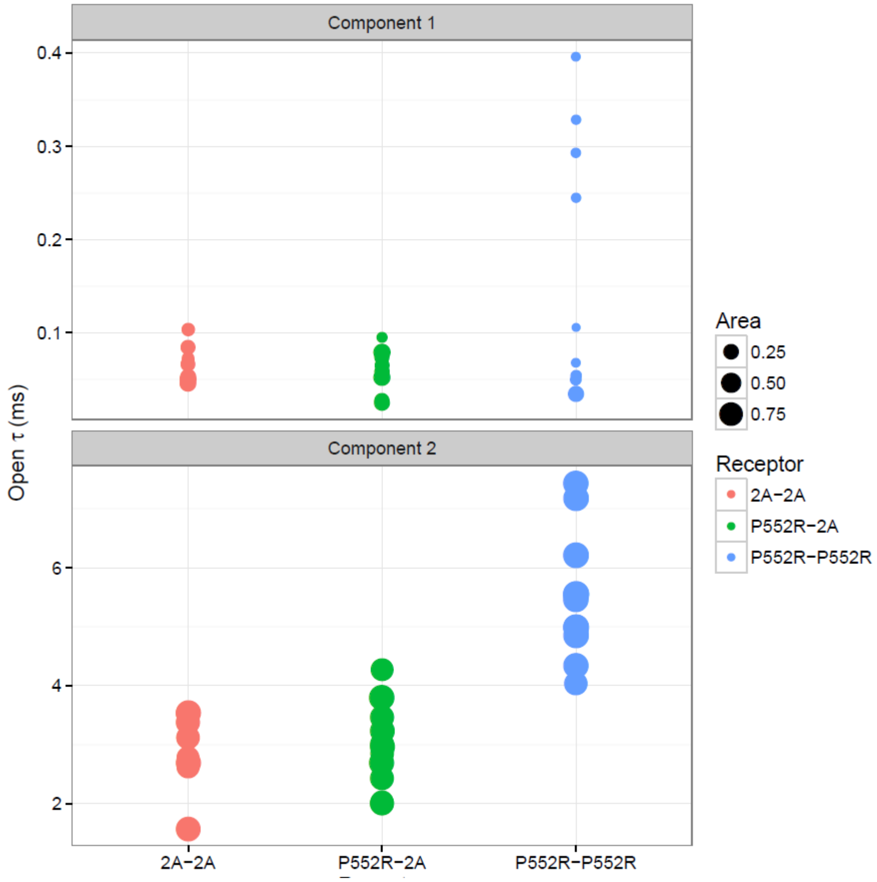

S2 Figure. Fitted time constants for GluN2A-P552R open time histograms (related to Figure-4) The open times for each patch were modelled as a mixture of two exponential components. The maximum likelihood estimates for the means of the two exponential components and their corresponding weights were determined for each patch. The top panel shows the estimated mean, tau, of the first exponential component and the bottom panel shows the estimated mean of the second component. The size of each point corresponds to the estimated area of that component, and points are colored by the receptor type.

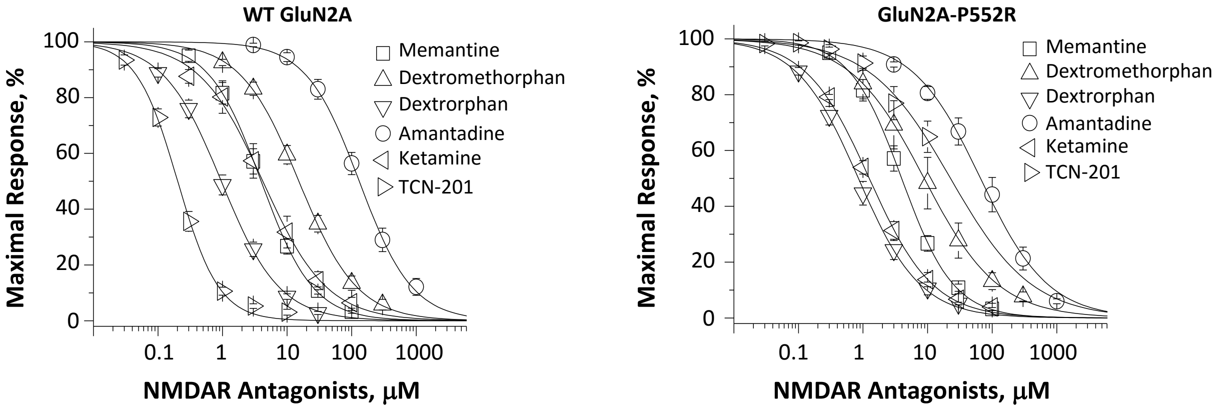

S3 Figure. (related to Figure-8 and RESULTS). Rescue pharmacology to evaluate the ability of NMDAR antagonists including FDA-approved drugs on inhibition of human NMDAR function by using two-electrode voltage clamp current recordings (holding at -40 mV) on *Xenopus* oocytes. The data are expressed as IC_{50} value ± SEM (n, maximal inhibition % at 100 μM for memantine, 300 μM for dextromenthorphan, 30 μM for dextrorphan, 1000 μM for amantadine, 100 μM for ketamine, 10 μM for TCN-201).

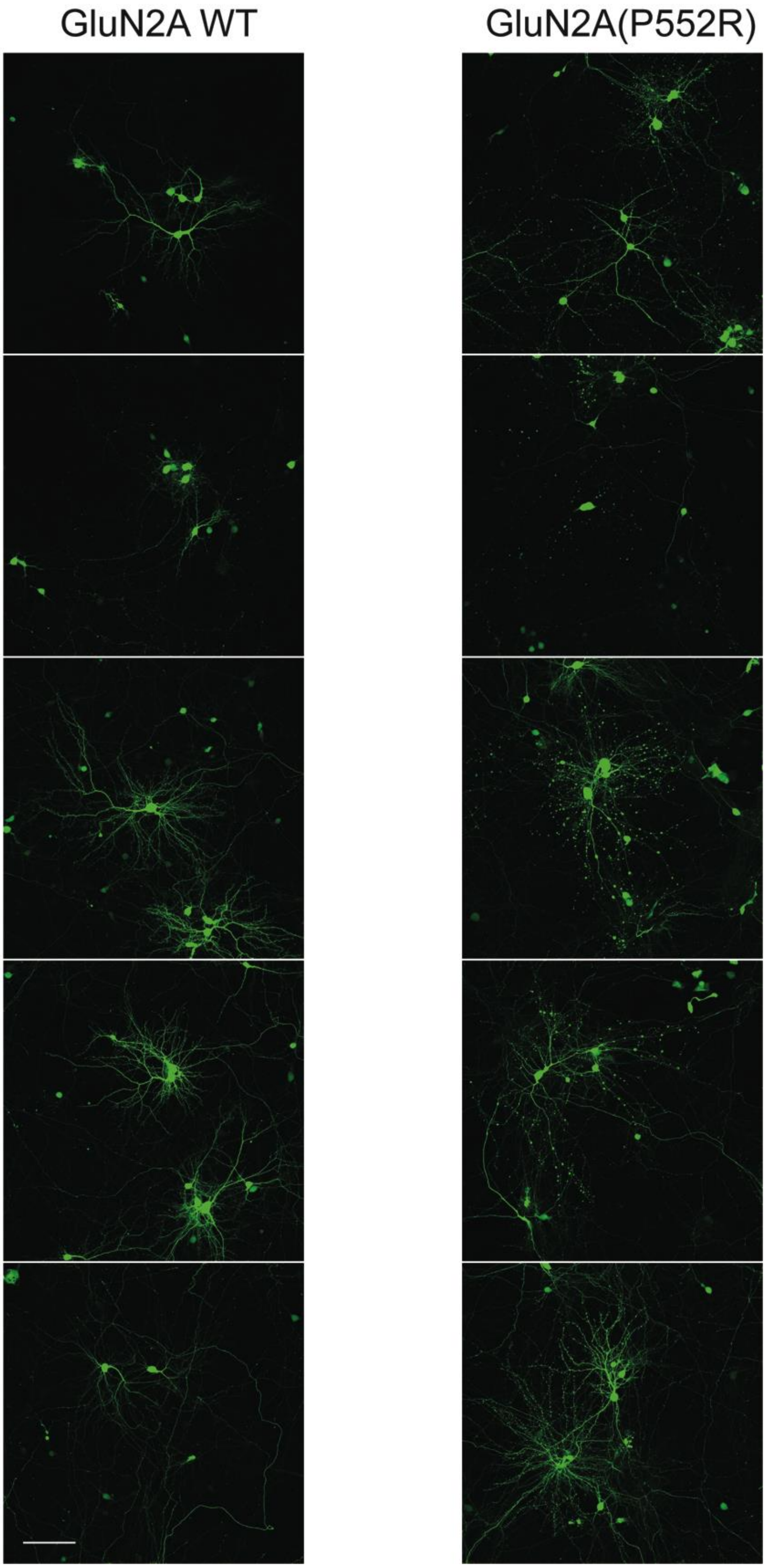

S4 Figure. Comparison of blebbing produced by transfection of neurons with GluN2A-P552R cDNA (related to **Figure-8, S5 Fig,** and **RESULTS**)**.** Morphological features of rat cortical neurons in culture (DIV 18-19) expressing GFP and either GluN2A WT (0.6 μ g; see **Methods** and **Fig. 8**), or GluN2A-P552R (0.6 μ g) for 24 hours. Blebs are a telltale and nearly ubiquitous sign of neuronal expression of GluN2A-P552R, but not GluN2A WT. Panels are representative of 5 independent transfection experiments for each vector, not necessarily paired across rows. Scale bar = 100 μm.

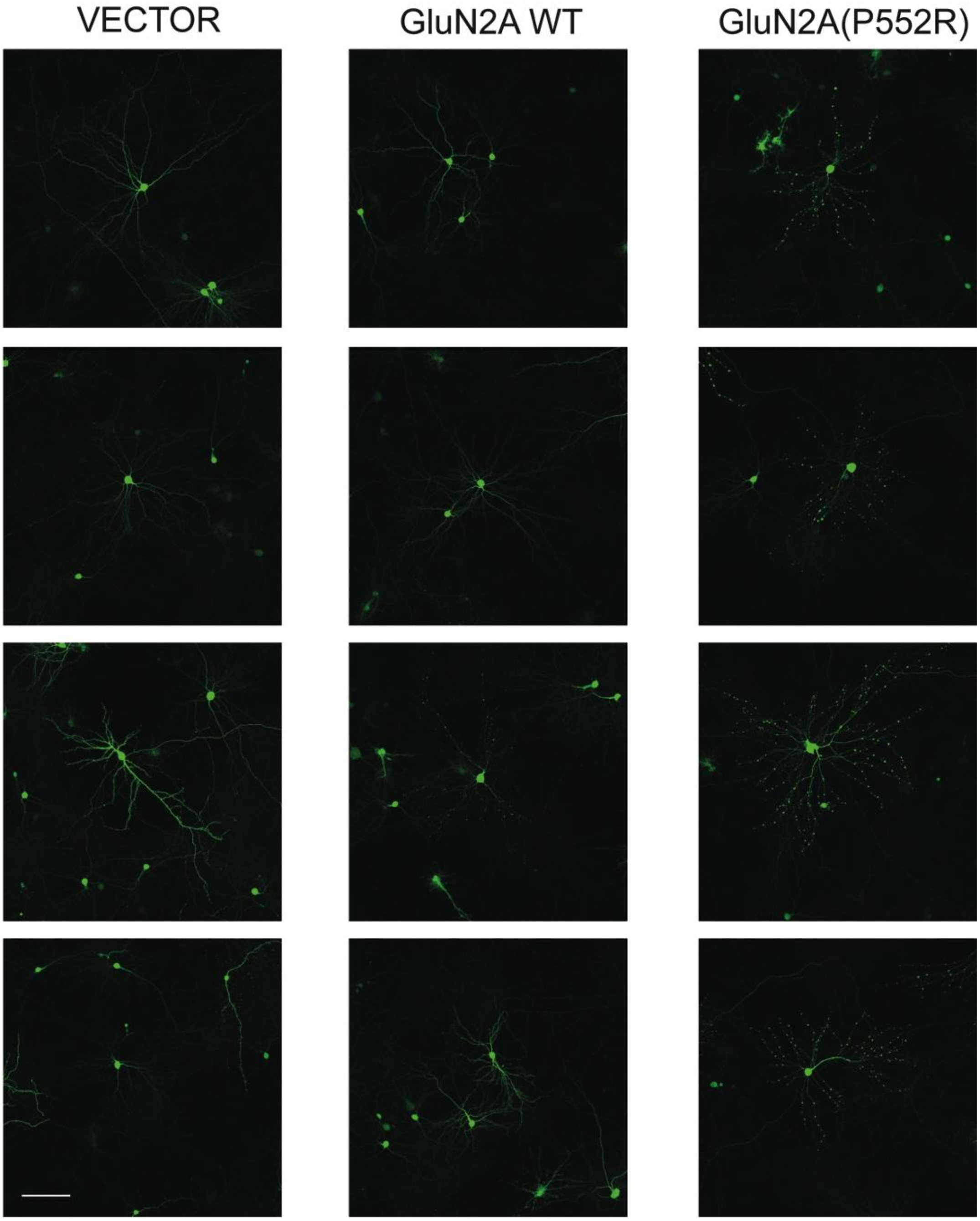

S5 Figure. Quantification of GluN2A-P552R induced blebbing in transfected neurons (related to **Figure-8, S4 Fig,** and **RESULTS**). Morphological features of rat cortical neurons in culture (DIV 18-19) expressing GFP and either empty vector, GluN2A WT (0.3 μ g; see Methods and Fig. 8), or GluN2A-P552R (0.3 μ g) for 24 hours. Although very rarely some dendritic blebs are observed in WT GluN2A-expressing neurons (e.g. see third panel from the top, middle row), blebs are a telltale and nearly ubiquitous sign of neuronal expression of GluN2A-P552R. Panels are representative of 11 different fields obtained from three separate coverslips for each condition for one representative experiment. We utilized an unbiased object count program (NIS elements, Nikon) to obtain the total number of blebs per field. The mean intensity for each field was utilized to set the threshold intensity and all objects from 2-100 μm were counted (cell bodies were excluded). Circularity was set to 0.25, with 1 being a perfect circle. Although these parameters detected objects in vector-expressing cells, these were attributed to spines or intrinsic dendritic tortuosity. Vector: 29.8 ± 6.2 objects/field; GluN2A WT: 59.2 ± 11.8; GluN2A(P552R): 115.4 ± 12.5. No statistical difference was observed between vector and WT; significant differences were observed between vector and mutant, and WT and mutant ($p<0.001$, 0.01, respectively; ANOVA/Tukey). Please note that we used a different pinhole in the confocal microscope (1.7) than the one used in S4 Figure (1.2) to increase the signal-to-noise ratio, which aided in the quantification procedure. Scale bar = 100 μm.

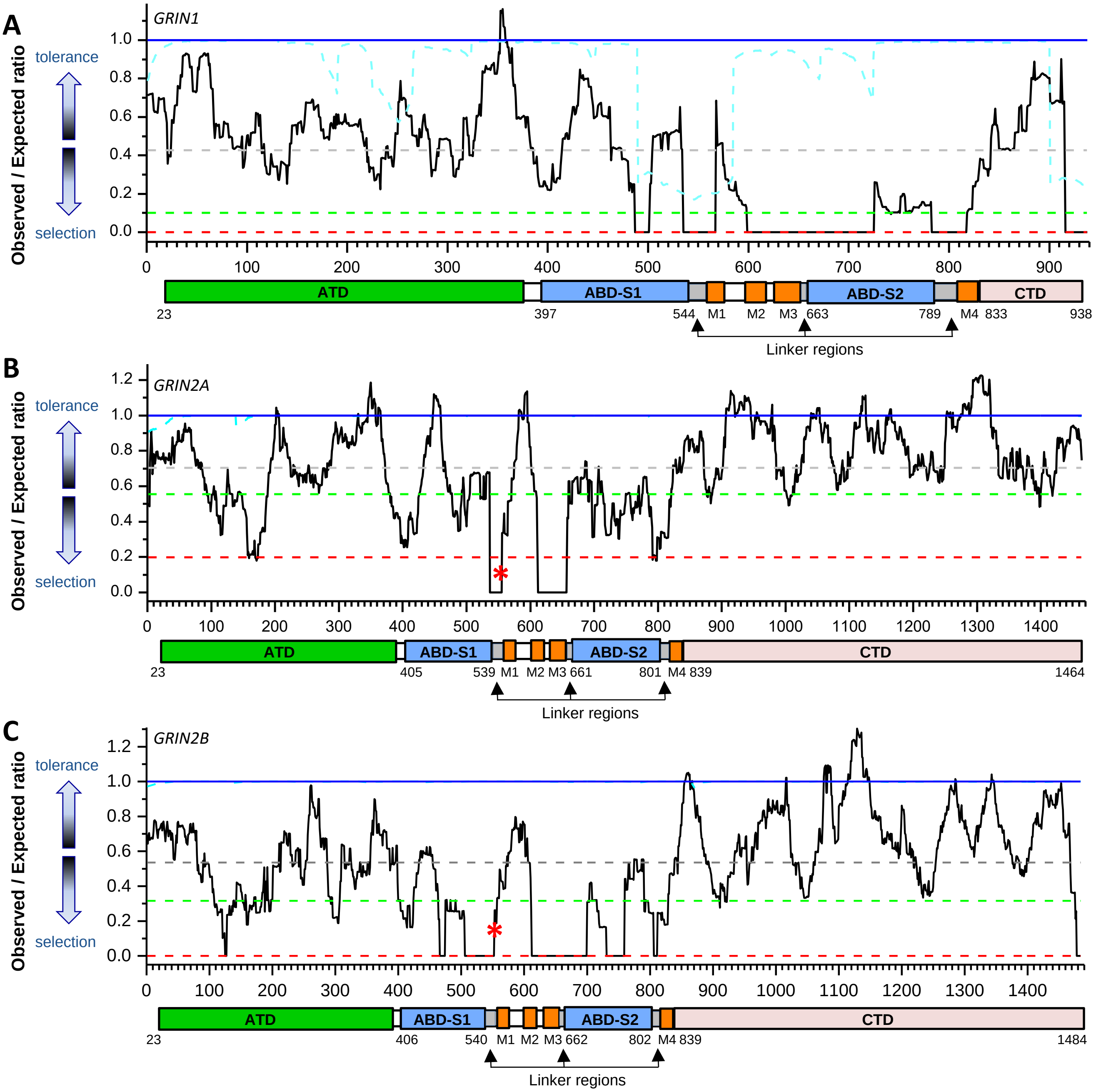

S6 Figure. OE-ratio calculated from ExAC (related to **METHODS**, **Figure-1,** and **RESULTS**). **A**, GluN1, **B**, GluN2A and **C**, GluN2B sliding window OE-ratio estimates (black full line), neutrality expected OE-ratio estimates derived from ExAC server accessed April, 2016 (blue full line), median OE-ratio for the gene (dark grey dashed line), 25th percentile of OE-ratio (green dashed line), 5th percentile of OE-ratio (red dashed line). The proportion of the 60,706 ExAC samples that had at least 10-fold coverage to be able to call a variant at the residue (cyan dashed line). The asterisk shows the Pro552 in GluN2A and Pro 553 in GluN2B.

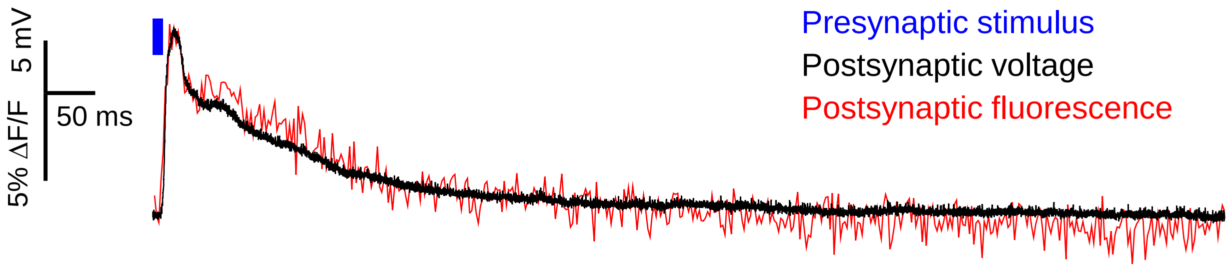

S7 Figure. Simultaneous electrical and optical voltage recording form neurons (related to **Figure-7** and **RESULTS**). Simultaneous fluorescence and manual patch clamp measurements of an excitatory post-synaptic potential in a neuron expressing a QuasAr voltage indicator. Presynaptic CheRiff-expressing neurons were stimulated with a single flash of blue light at the start of the measurement. See Lou et al (2016) for current recording methods.

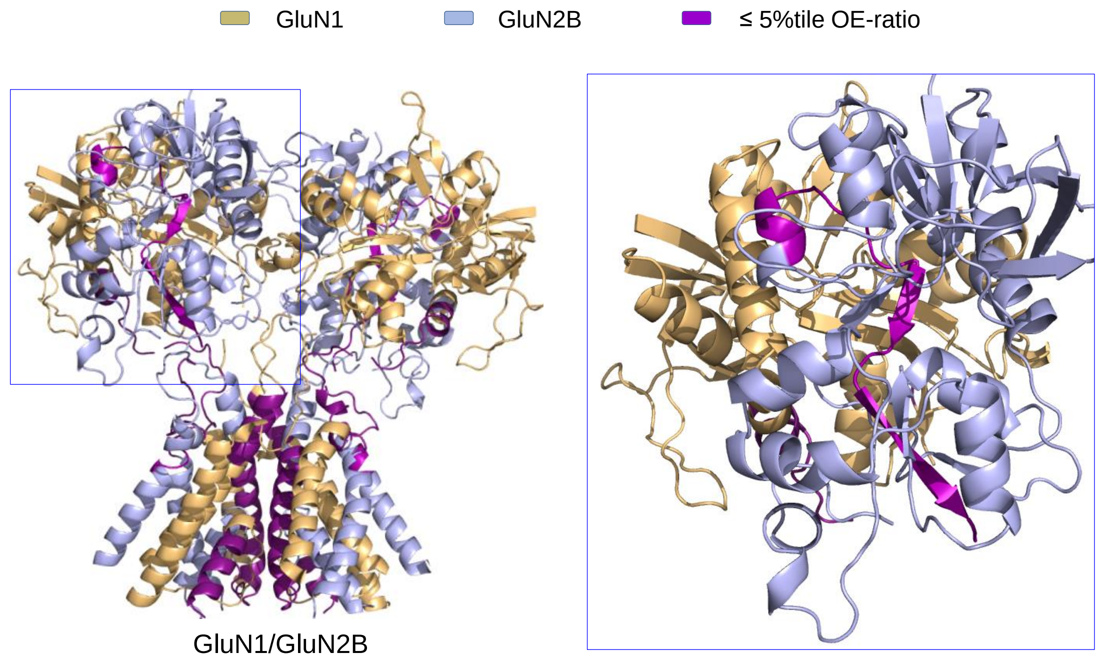

S8 Figure. Regional purifying selection of GluN1/GluN2B (related to **Figure-9** and **Discussion**). Left panel shows a ribbon structure of the GluN1/GluN2B receptors without the amino terminal domain. GluN1 is tan and GluN2B is light blue; regions with purple color reflect an OE-ratio below the 5th percentile, indicating the regions under the strongest purifying selection. Side view of agonist binding domain (ABD) in an expanded panel (*right*) shows a strong purifying selection (in purple) in regions of GluN1 S2 connecting with pre-M4 helix and GluN2B S1 connecting with pre-M1 helix.

S1 Table. Patient ascertained de novo mutations (related to Table-1)

HGNC	GRCh37/hg19	HGVSc	HGVSp	Database Phenotype	PUBMED
GRIN1	chr9:140057162G>A	NM_007327.3:c.1984G>A	NP_015566.1:p.Glu662Lys	Mental retardation, autosomal dominant 8	21376300
GRIN1	chr9:140058120G>A	NM_007327.3:c.2443G>A	NP_015566.1:p.Gly815Arg	Musculoskeletal/Structural (child onset); Seizures	25356970
GRIN1	chr9:140056647C>G	NM_007327.3:c.1656C>G	NP_015566.1:p.Asp552Glu	Epileptic encephalopathy early onset with involuntary movements developmental delay & intellectual disability	25864721
GRIN1	chr9:140056647C>A	NM_007327.3:c.1656C>A	NP_015566.1:p.Asp552Glu	Epileptic encephalopathy nonsyndromic	26482601
GRIN1	chr9:140056661C>G	NM_007327.3:c.1670C>G	NP_015566.1:p.Pro557Arg	Intellectual disability	25167861
GRIN1	chr9:140057101G>A	NM_007327.3:c.1923G>A	NP_015566.1:p.Met641Ile	Epileptic encephalopathy early onset with involuntary movements developmental delay & intellectual disability	25864721
GRIN1	chr9:140057118A>C	NM_007327.3:c.1940A>C	NP_015566.1:p.Tyr647Ser	Infantile spasms	23934111
GRIN1	chr9:140057128C>G	NM_007327.3:c.1950C>G	NP_015566.1:p.Asn650Lys	Epileptic encephalopathy early onset with involuntary movements developmental delay & intellectual disability	25864721
GRIN1	chr9:140058120G>C	NM_007327.3:c.2443G>C	NP_015566.1:p.Gly815Arg	Epileptic encephalopathy early onset with involuntary movements developmental delay & intellectual disability	25864721
GRIN1	chr9:140058090C>T	NM_007327.3:c.2413C>T	NP_015566.1:p.Pro805Ser	Developmental Delay	DDD - biorxiv
GRIN1	chr9:140057361T>C	NM_007327.3:c.2077T>C	NP_015566.1:p.Phe693Leu	Developmental Delay	DDD - biorxiv
GRIN1	chr9:140057658G>A	NM_007327.3:c.2209G>A	NP_015566.1:p.Glu737Lys	Intellectual disability	27479843
GRIN2A	chr16:9943635A>G	NM_000833.4:c.1306T>C	NP_000824.1:p.Cys436Arg	Partial epilepsy atypical benign	23933819
GRIN2A	chr16:9934513C>T	NM_000833.4:c.1642G>A	NP_000824.1:p.Ala548Thr	Landau-Kleffner syndrome	23933820
GRIN2A	chr16:9928084G>C	NM_000833.4:c.1655C>G	NP_000824.1:p.Pro552Arg	Focal epilepsy with speech disorder with or without mental retardation	23033978
GRIN2A	chr16:9923442G>T	NM_000833.4:c.1845C>A	NP_000824.1:p.Asn615Lys	Focal epilepsy with speech disorder with or without mental retardation	20890276
GRIN2A	chr16:9923342G>C	NM_000833.4:c.1945C>G	NP_000824.1:p.Leu649Val	Focal epilepsy with speech disorder with or without mental retardation	23033978
GRIN2A	chr16:9923333A>C	NM_000833.4:c.1954T>G	NP_000824.1:p.Phe652Val	Focal epilepsy with speech disorder with or without mental retardation	23933820
GRIN2A	chr16:9923330T>C	NM_000833.4:c.1957A>G	NP_000824.1:p.Met653Val	Developmental Delay	DDD - biorxiv
GRIN2A	chr16:9923328C>T	NM_000833.4:c.1959G>A	NP_000824.1:p.Met653Ile	Intellectual disability	27479843
GRIN2A	chr16:9916208A>G	NM_000833.4:c.2081T>C	NP_000824.1:p.Ile694Thr	Landau-Kleffner syndrome	23933820
GRIN2A	chr16:9916194G>A	NM_000833.4:c.2095C>T	NP_000824.1:p.Pro699Ser	Benign epilepsy with centrotemporal spikes	23933819
GRIN2A	chr16:9862869G>T	NM_000833.4:c.2434C>A	NP_000824.1:p.Leu812Met	Epileptic encephalopathy	24504326
GRIN2A	chr16:9862854T>C	NM_000833.4:c.2449A>G	NP_000824.1:p.Met817Val	Global developmental delay & epilepsy	24903190
GRIN2A	chr16:9862853A>G	NM_000833.4:c.2450T>C	NP_000824.1:p.Met817Thr	Intellectual disability	27479843
GRIN2B	chr12:13769479T>C	NM_000834.3:c.1238A>G	NP_000825.2:p.Glu413Gly	Mental retardation, autosomal dominant 6	ClinVar Submission
GRIN2B	chr12:13768560C>T	NM_000834.3:c.1367G>A	NP_000825.2:p.Cys456Tyr	Mental retardation, autosomal dominant 6	23160955
GRIN2B	chr12:13768545C>A	NM_000834.3:c.1382G>T	NP_000825.2:p.Cys461Phe	Lennox-Gastaut syndrome	23934111
GRIN2B	chr12:13768132C>T	NM_000834.3:c.1570G>A	NP_000825.2:p.Asp524Asn	Intellectual disability	27479843
GRIN2B	chr12:13768083C>T	NM_000834.3:c.1619G>A	NP_000825.2:p.Arg540His	Epileptic encephalopathy, early infantile, 27	24272827
GRIN2B	chr12:13764781G>A	NM_000834.3:c.1658C>T	NP_000825.2:p.Pro553Leu	Mental retardation, autosomal dominant 6	23033978
GRIN2B	chr12:13764767C>T	NM_000834.3:c.1672G>A	NP_000825.2:p.Val558Ile	Intellectual disability	27479843
GRIN2B	chr12:13761703T>A	NM_000834.3:c.1844A>T	NP_000825.2:p.Asn615Ile	Epileptic encephalopathy, early infantile, 27	24272827
GRIN2B	chr12:13761702G>C	NM_000834.3:c.1845C>G	NP_000825.2:p.Asn615Lys	Developmental Delay	DDD - biorxiv
GRIN2B	chr12:13761694A>C	NM_000834.3:c.1853T>G	NP_000825.2:p.Val618Gly	Epileptic encephalopathy, early infantile, 27	24272827
GRIN2B	chr12:13761664G>A	NM_000834.3:c.1883C>T	NP_000825.2:p.Ser628Phe	Developmental Delay	DDD - biorxiv
GRIN2B	chr12:13761641C>G	NM_000834.3:c.1906G>C	NP_000825.2:p.Ala636Pro	Intellectual disability	23718928
GRIN2B	chr12:13761562T>G	NM_000834.3:c.1985A>C	NP_000825.2:p.Gln662Pro	Partial seizures & infantile spasms with intellectual / developmental disabilities	26544041
GRIN2B	chr12:13724865G>A	NM_000834.3:c.2044C>T	NP_000825.2:p.Arg682Cys	Mental retardation, autosomal dominant 6	20890276
GRIN2B	chr12:13724856T>G	NM_000834.3:c.2053A>C	NP_000825.2:p.Thr685Pro	Epileptic encephalopathy, early infantile, 27	ClinVar Submission
GRIN2B	chr12:13724849G>C	NM_000834.3:c.2060C>G	NP_000825.2:p.Pro687Arg	Developmental Delay	DDD - biorxiv
GRIN2B	chr12:13724844C>T	NM_000834.3:c.2065G>A	NP_000825.2:p.Gly689Ser	Developmental Delay	DDD - biorxiv
GRIN2B	chr12:13724793T>C	NM_000834.3:c.2116A>G	NP_000825.2:p.Met706Val	Intellectual disability	27479843
GRIN2B	chr12:13720138C>T	NM_000834.3:c.2419G>A	NP_000825.2:p.Glu807Lys	Developmental Delay	DDD - biorxiv
GRIN2B	chr12:13720098C>T	NM_000834.3:c.2459G>A	NP_000825.2:p.Gly820Glu	Intellectual disability	25356899
GRIN2B	chr12:13720098C>G	NM_000834.3:c.2459G>C	NP_000825.2:p.Gly820Ala	Developmental Delay	DDD - biorxiv

DDD – biorxiv: http://biorxiv.org/content/biorxiv/early/2016/04/22/049056.full.pdf

The rows highlighted in orange are the S1-M1 mutations associated with diseases

S2 Table. Deactivation time course after glycine removal for GluN1-P557R and GluN2A-P552R (related to **Figure-4)**

	Di-heteromeric Receptors			Tri-heteromeric Receptors		
	**WT N1/N2A**	**N1/N2A-P552R**	**N1-P557R/N2A**	**N2A/N2A**	**N2A-P552R/N2A**	**N2A-P552R/N2A-P552R**
**Amplitude (peak, pA/pF)**	67 ± 12	18 ± 5.1#	4.5 ± 2.0#	57 ± 15	29 ± 4.3	11 ± 2.7#$
**Amplitude (SS, pA/pF)**	44 ± 8.5	---	3.9 ± 1.7*	49 ± 11	24 ± 3.4	---
**I_{SS}/I_{PEAK}%**	70 ± 4.5 %	---	88 ± 1.2 %*	88 ± 2.8 %	85 ± 4.9 %	---
**Rise time (ms)**	13 ± 0.75	874 ± 75#	8.6 ± 1.4	15 ± 1.1	14 ± 1.3	1176 ± 22#$
**τ_{FAST} (ms)**	91 ± 5.2	1093 ± 96#	415 ± 148#	127 ± 9.6	387 ± 62	1690 ± 90#$
**τ_{SLOW} (ms)**	594 ± 87	3034 ± 1263#	953 ± 99	1346 ± 242	1800 ± 254	2368 ± 304#
**%τ_{FAST}**	92 ± 1.9 %	89 ± 6.5 %	57 ± 14.5 %#	95 ± 1.5 %	81 ± 3.0 %	91 ± 3.8 %
**τ_W(ms)**	125 ± 7.6	1243 ± 84#	801 ± 103#	177 ± 9.5	621 ± 75#	1915 ± 85#$
**n**	17	12	7	8	7	17

Data are from human NMDARs. All parameters describing the time course of the macroscopic current were from responses to 1.5 sec glutamate application.

# $p < 0.05$ compared to corresponding WT receptors; one way ANOVA, Tukey post hoc

$ $p < 0.05$ compared to N2A-P552R/N2A; one way ANOVA, Tukey post hoc

* $p < 0.05$ compared to corresponding WT receptors, unpaired t-test

See **S6 Table** for F statistics.

S3 Table. Sensitivity of GluN2A-P552R to endogenous negative modulators (related to **RESULTS)**

	WT N1/N2A	N1/N2A-P552R	P-value[#]
**Mg^{2+}, IC_{50}, μM (n)**	20 ± 3.8 (6)	13 ± 2.2 (8)	0.062
**Proton, $I_{pH6.8}/I_{pH7.6}$% (n)**	44 ± 1.0% (12)	47 ± 1.6% (11)	0.108
**Zn^{2+}, IC_{50}, nM (n)**	27 ± 5.7 (24)	8.9 ± 0.4 (24)	< 0.001
**%inhibition by saturating Zn^{2+}**	60 ± 2.7% (23)	91 ± 1.7% (22)	< 0.001

Data are from human NMDARs.

[#] p-values for unpaired t-tests comparing WT N1/N2A to N1/N2A-P552R

S4 Table. Statistical analysis for data in Table-3.

	GluN1/GluN2A					GluN1/GluN2B				
	ANOVA		Post hoc Tukey's P-value			ANOVA		Post hoc Tukey's P-value		
	F statistic	P value	GluN1-D552E/N2A	GluN1-P557R/N2A	GluN1/N2A-A548T	F statistic	P value	GluN1-D552E/N2B	GluN1-P557R/N2B	GluN1/N2B-P553L
**Amplitude (peak)**	F (3,32) = 6.884	0.001	0.0082	0.0074	0.0028	F (3,27) = 10.33	0.0001	0.0024	0.001	0.0009
**Glutamate, EC_{50}**	F (3,50) = 307.8	<0.0001	<0.0001	<0.0001	<0.0001	F (2,21) = 256.7	<0.0001	0.0001	<0.0001	-
**Glycine, EC_{50}**	F (3,38) = 146.6	<0.0001	<0.0001	<0.0001	<0.0001	F (2,21) = 86.34	<0.0001	0.0048	<0.0001	-

S5 Table. Statistical analysis for data in Table-4.

	Di-heteromeric NMDA Receptors	Tri-heteromeric NMDA Receptors				
	GluN2A vs GluN2A-P552R	ANOVA		Post hoc Tukey's P-value		
	unpaired t-test p value	F statistic	P value	N2A/N2A vs N2A-P552R/N2A	N2A/N2A vs N2A-P552R/N2A-P552R	N2A/N2A-P552R vs N2A-P552R/N2A-P552R
**Glutamate, EC_{50}**	< 0.0001	F (2,77) = 136.9	<0.0001	<0.0001	<0.0001	<0.0001
**Glycine, EC_{50},**	< 0.0001	F (2,71) = 331	<0.0001	<0.0001	<0.0001	<0.0001
**Amplitude (peak, pA/pF)**	0.9289	F (2,22) = 0.011	0.9891	---	---	---
**Amplitude (SS, pA/pF)***	---	---	---	0.6242	---	---
**I_{SS}/I_{PEAK}%***	---	---	---	0.1478	---	---
**Rise time (ms)**	< 0.0001	F (2,23) = 1662	<0.0001	0.9958	<0.0001	<0.0001
**t_{FAST} (ms)**	< 0.0001	F (2,23) = 110.7	<0.0001	0.4742	<0.0001	<0.0001
**t_{SLOW} (ms)**	---	F (2,14) = 20.7	<0.0001	0.7029	0.0001	0.0002
**%t_{FAST}**	0.0547	F (2,23) = 11.12	0.0004	0.001	0.9869	0.0014
**t_W(ms)**	< 0.0001	F (2,23) = 152.7	<0.0001	0.0672	<0.0001	<0.0001
**Charge transfer pA ms/pF**	0.00061	F (2,20) = 3.707	0.0427	0.5520	<0.05	0.1938
**Mean Open Time (ms)**	---	F (2,28) = 39.18	<0.0001	0.9996	<0.0001	<0.0001
**Current Amplitude (pA)**	---	F(2,28) = 32.01	<0.0001	0.4623	<0.0001	<0.0001
**Open t1 (ms)**	---	F (2,28) = 3.816	0.0342	0.9861	0.1011	0.0424
**Open t2 (ms)**	---	F (2,28) = 33.33	<0.0001	0.9999	<0.0001	<0.0001
**Open Area 1 (%)**	---	F (2,28) = 11.21	0.0003	0.9915	0.0016	0.0007
**Open probability**	0.2184	---	---	---	---	---

*unpaired t-test

S6 Table. Statistical analyses for S2 Table.

	Di-heteromeric Receptors				Tri-heteromeric Receptors				
	ANOVA		p value		ANOVA		p value		
	F statistic	P value	N1/N2A vs N1/N2A-P552R	N1/N2A vs N1-P557R/N2A	F statistic	P value	N2A/N2A vs N2A-P552R/N2A	N2A/N2A vs N2A-P552R/N2A-P552R	N2A-P552R/N2A vs N2A-P552R/N2A-P552R
**Amplitude (peak, pA/pF)**	F (2,33) = 9.869	0.0004	0.0037	0.0018	F (2,29) = 11.37	0.0002	0.0542	0.0001	0.2076
**Amplitude (SS, pA/pF)***	---	---	---	0.0073	---	---	0.0761	---	---
**I_{SS}/I_{PEAK}%***	---	---	---	0.0152	---	---	0.5758	---	---
**Rise time (ms)**	F (2,35) = 143.9	<0.0001	<0.0001	0.9975	F (2,39) = 1369	<0.0001	0.9987	<0.0001	<0.0001
**t_{FAST} (ms)**	F (2,35) = 44.7	<0.0001	<0.0001	0.0224	F (2,39) = 118.5	<0.0001	0.1479	<0.0001	<0.0001
**t_{SLOW} (ms)**	F (2,19) = 6.178	0.0086	0.0068	0.8666	F (2,22) = 8.969	0.0014	0.7856	0.0012	0.0061
**%t_{FAST}**	F (2,35) = 6.432	0.0042	0.9517	0.0044	F (2,39) = 2.988	0.062	---	---	---
**t_W(ms)**	F (2,35) = 93.03	<0.0001	<0.0001	<0.0001	F (2,39) = 147.9	<0.0001	0.0045	<0.0001	<0.0001

* unpaired t-tests

S7 Table. Statistical analysis for Table-6.

	unpaired t-test p value	
	**GluN1/GluN2A vs. GluN1-P557R/GluN2A**	**GluN1/GluN2B vs. GluN1/GluN2B-P553R**
**Amplitude (peak, pA/pF)**	0.0443	0.5815
**Amplitude (SS, pA/pF)**	0.0321	---
**I_{SS}/I_{PEAK}%**	0.0006	---
**Rise time (ms)**	0.8589	< 0.0001
**t_{FAST} (ms)**	< 0.0001	< 0.0001
**t_{SLOW} (ms)**	0.4272	0.0013
**%t_{FAST}**	0.0201	0.9024
**t_W (ms)**	< 0.0001	< 0.0001
**Charge transfer, pA·ms/pF**	0.48208	---

S8 Table. Statistical analysis for Table-7.

	Q and K ANOVA		Post hoc Tukey's P-value vs WT 2A		G, A, I, and L ANOVA		Post hoc Tukey's P-value vs WT 2A			
	**F statistic**	**P value**	**P552Q**	**P552K**	**F statistic**	**P value**	**P552G**	**P552A**	**P552I**	**P552L**
**Glutamate, EC_{50}**	F (6,36) = 124.8	<0.0001	<0.0001	0.9473	<0.0001	<0.0001	<0.0001	0.9473	<0.0001	0.0606
**Glycine, EC_{50}**	F (6,79) = 83.39	<0.0001	<0.0001	<0.0001	0.9999	0.0275	<0.0001	<0.0001	0.0003	0.3416
**Amplitude (peak, pA/pF)**	F (2, 27) = 19.16	<0.0001	0.0002	<0.0001	F (4, 58) = 1.517	0.2091	---	---	---	---
**Amplitude (SS, pA/pF)**	---	---	0.0002*	---	F (4, 58) = 43.78	<0.0001	<0.0001	<0.0001	<0.0001	<0.0001
**I_{SS}/I_{PEAK}**	---	---	<0.0001*	---	F (4, 58) = 43.78	<0.0001	<0.0001	<0.0001	<0.0001	<0.0001
**t_W desensitization (ms)**	---	---	<0.0001	---	F (4, 59) = 29.62	<0.0001	<0.0001	<0.0001	0.998	<0.0001
**Rise time (ms)**	F (2, 28) = 6131	<0.0001	0.9786	<0.0001	F (4, 57) = 5.376	0.001	0.0022	0.9969	0.5998	0.9994
**t_{FAST} (ms)**	F (2, 28) = 153.2	<0.0001	0.9945	<0.0001	F (4, 58) = 10.72	<0.0001	<0.0001	0.0806	0.0003	0.0009
**t_{SLOW} (ms)**	---	---	0.6758*	---	F (4, 44) = 3.181	0.0222	0.7861	0.9441	0.8359	0.0395
**%t_{FAST}**	F (2, 28) = 3.667	0.0385	0.0634	0.999	F (4, 59) = 1.547	0.2004	---	---	---	---
**t_W(ms)**	F (2, 28) = 219.9	<0.0001	0.8239	<0.0001	F (4, 58) = 4.52	0.003	0.079	0.7566	0.0021	0.213

*unpaired t-test

S9 Table. Statistical Data for Figure-8.

A. Repeated Measures ANOVA/Bonferroni: Luciferase Assays (0.3 µg DNA/well)

	P value	F statistic	WT GluN2A (- mem) vs. WT GluN2A (+ mem)	WT GluN2A (- mem) vs. GluN2A-P552R (- mem)	GluN2A-P552R (- mem) vs. GluN2A-P552R (+ mem)
Viability (% of control; n=7)	< 0.0001	F (3, 6, 18) = 64.07	< 0.001	< 0.05	< 0.001

B. Repeated Measures ANOVA/Bonferroni: Luciferase Assays (0.6 µg DNA/well)

	P value	F statistic	WT GluN2A (- mem) vs. WT GluN2A (+ mem)	WT GluN2A (- mem) vs. GluN2A-P552R (- mem)	GluN2A-P552R (- mem) vs. GluN2A-P552R (+ mem)
Viability (% of control; n=8)	< 0.0001	F (3, 7, 21) = 23.11	< 0.01	ns	< 0.001

C. Repeated Measures ANOVA/Bonferroni: Cell Counts (0.6 µg DNA/well)

	P value	F statistic	WT GluN2A (- mem) vs. WT GluN2A (+ mem)	WT GluN2A (- mem) vs. GluN2A-P552R (- mem)	GluN2A-P552R (- mem) vs. GluN2A-P552R (+ mem)
Viability (% of control; n=6)	0.0079	F (3, 5, 15) = 5.77	ns	ns	< 0.01
