# Supplementary material for: Molecular Mechanism of Disease-Associated Mutations in the Pre-M1 Helix of NMDA Receptors and Potential Rescue Pharmacology
Source: PLoS Genet. 2017 Jan 17;13(1):e1006536. doi: 10.1371/journal.pgen.1006536 (PMC5240934; doi:10.1371/journal.pgen.1006536)
Supplement: S4 Fig — Additional experimental results are shown for neurons transfected with GluN2A-P552R. Morphological features of rat cortical neurons in culture (DIV 18–19) expressing GFP and either GluN2A WT (0.6 μ g; see Methods and Fig 8), or GluN2A-P552R (0.6 μ g) for 24 hours. Blebs are a telltale and nearly ubiquitous sign of neuronal expression of GluN2A-P552R, but not GluN2A WT. Panels are representative of 5 independent transfection experiments for each vector, not necessarily paired across rows. Scale bar = 100 μm. (PDF) [file pgen.1006536.s004.pdf]

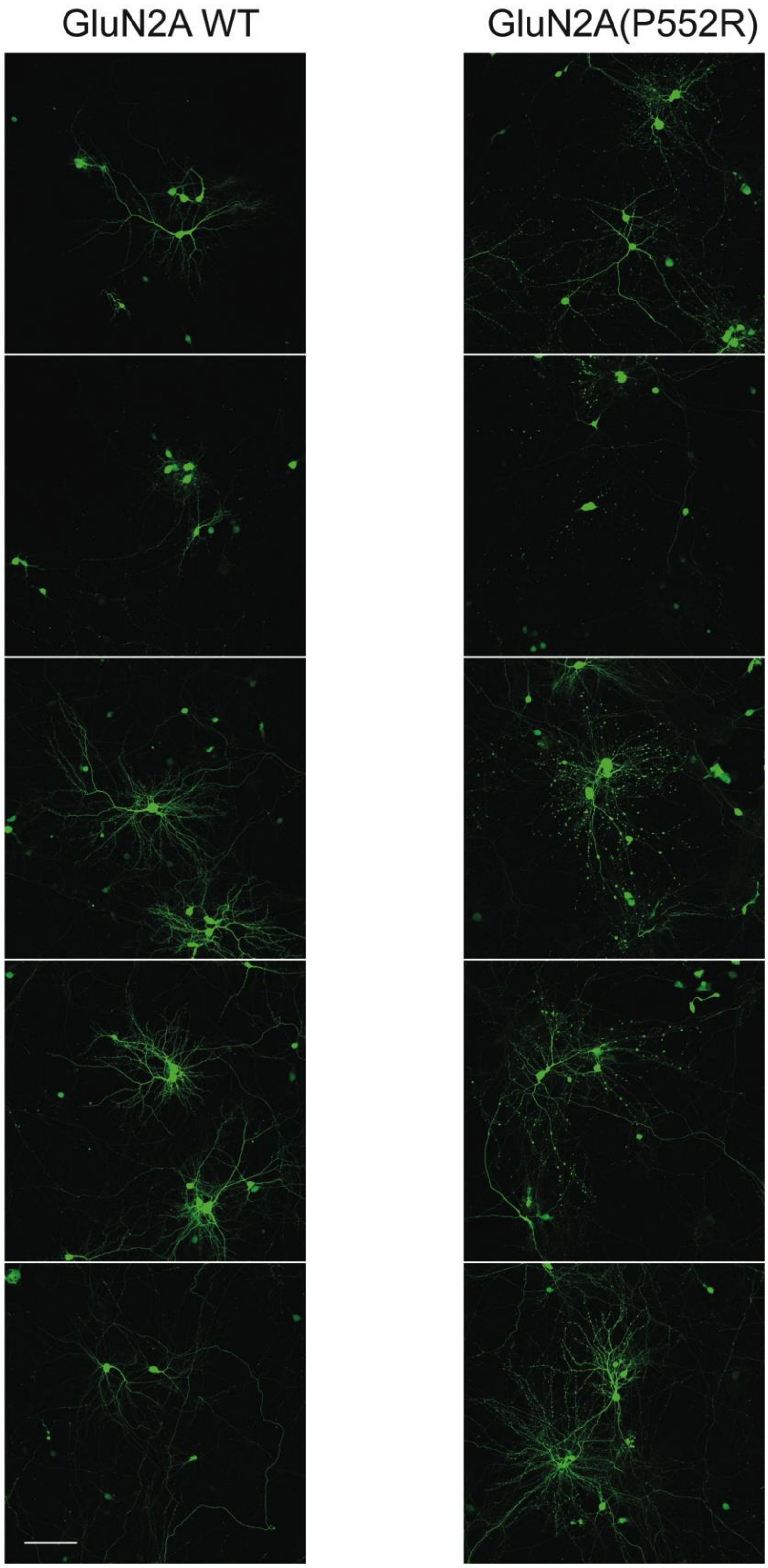

S4 Figure. Comparison of blebbing produced by transfection of neurons with GluN2A-P552R cDNA (related to **Figure-8, S5 Fig,** and **RESULTS**)**.** Morphological features of rat cortical neurons in culture (DIV 18-19) expressing GFP and either GluN2A WT (0.6 μ g; see **Methods** and **Fig. 8**), or GluN2A-P552R (0.6 μ g) for 24 hours. Blebs are a telltale and nearly ubiquitous sign of neuronal expression of GluN2A-P552R, but not GluN2A WT. Panels are representative of 5 independent transfection experiments for each vector, not necessarily paired across rows. Scale bar = 100 μm.
