# Supplementary material for: Molecular Mechanism of Disease-Associated Mutations in the Pre-M1 Helix of NMDA Receptors and Potential Rescue Pharmacology
Source: PLoS Genet. 2017 Jan 17;13(1):e1006536. doi: 10.1371/journal.pgen.1006536 (PMC5240934; doi:10.1371/journal.pgen.1006536)
Supplement: S5 Fig — Quantification of blebbing in neurons transfected with GluN2A WT and GluN2A-P552R cDNA. Morphological features of rat cortical neurons in culture (DIV 18–19) expressing GFP and either empty vector, GluN2A WT (0.3 μ g; see Methods and Fig 8), or GluN2A-P552R (0.3 μ g) for 24 hours. Although very rarely some dendritic blebs are observed in WT GluN2A-expressing neurons (e.g. see third panel from the top, middle row), blebs are a telltale and nearly ubiquitous sign of neuronal expression of GluN2A-P552R. Panels are representative of 11 different fields obtained from three separate coverslips for each condition for one representative experiment. We utilized an unbiased object count program (NIS elements, Nikon) to obtain the total number of blebs per field. The mean intensity for each field was utilized to set the threshold intensity and all objects from 2–100 μm were counted (cell bodies were excluded). Circularity was set to 0.25, with 1 being a perfect circle. Although these parameters detected objects in vector-expressing cells, these were attributed to spines or intrinsic dendritic tortuosity. Vector: 29.8 ± 6.2 objects/field; GluN2A WT: 59.2 ± 11.8; GluN2A(P552R): 115.4 ± 12.5. No statistical difference was observed between vector and WT; significant differences were observed between vector and mutant, and WT and mutant (p<0.001, 0.01, respectively; ANOVA/Tukey). Please note that we used a different pinhole in the confocal microscope (1.7) than the one used in S4 Fig (1.2) to increase the signal-to-noise ratio, which aided in the quantification procedure. Scale bar = 100 μm. (PDF) [file pgen.1006536.s005.pdf]

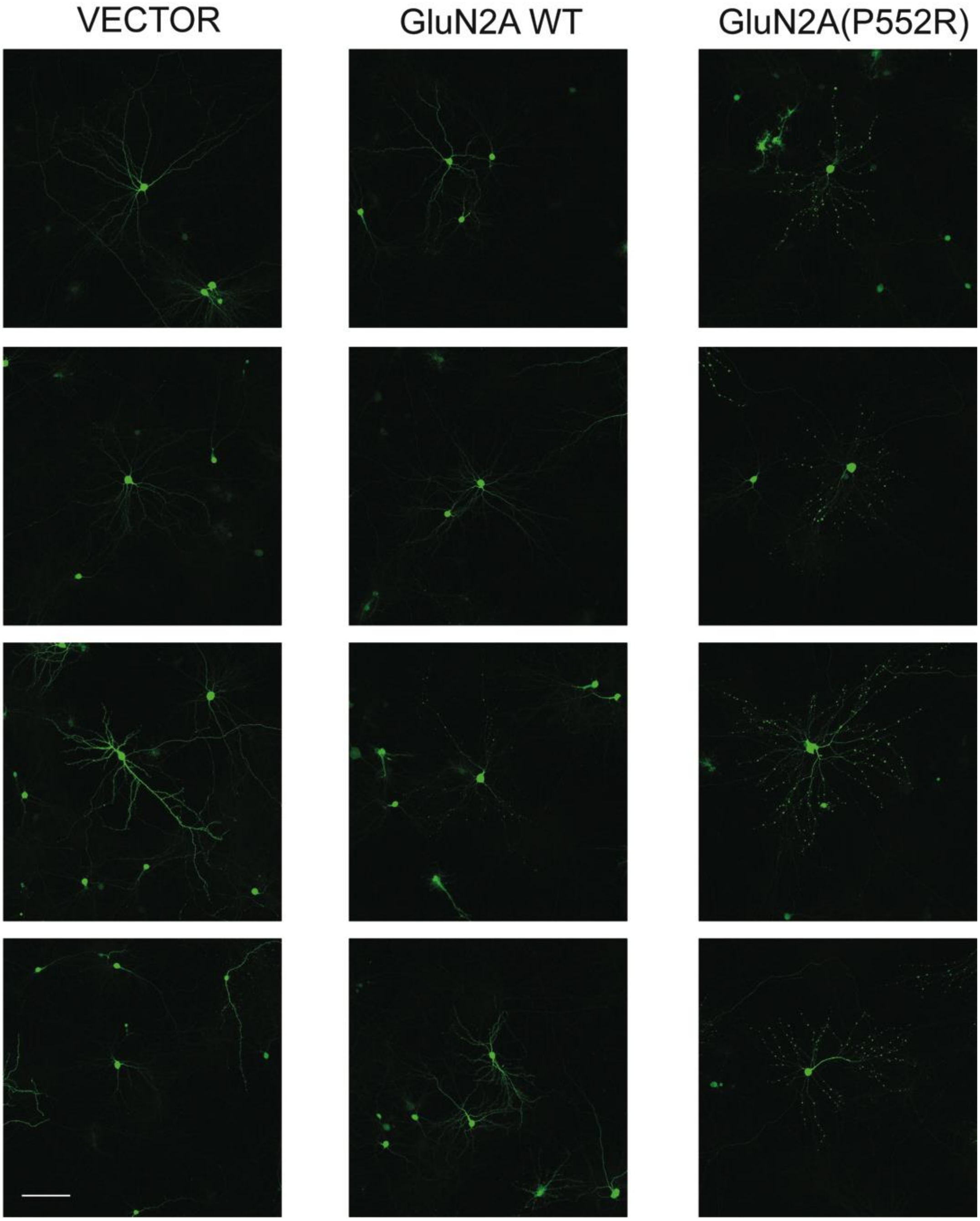

S5 Figure. Quantification of GluN2A-P552R induced blebbing in transfected neurons (related to **Figure-8, S4 Fig,** and **RESULTS**). Morphological features of rat cortical neurons in culture (DIV 18-19) expressing GFP and either empty vector, GluN2A WT (0.3 μ g; see Methods and Fig. 8), or GluN2A-P552R (0.3 μ g) for 24 hours. Although very rarely some dendritic blebs are observed in WT GluN2A-expressing neurons (e.g. see third panel from the top, middle row), blebs are a telltale and nearly ubiquitous sign of neuronal expression of GluN2A-P552R. Panels are representative of 11 different fields obtained from three separate coverslips for each condition for one representative experiment. We utilized an unbiased object count program (NIS elements, Nikon) to obtain the total number of blebs per field. The mean intensity for each field was utilized to set the threshold intensity and all objects from 2-100 μm were counted (cell bodies were excluded). Circularity was set to 0.25, with 1 being a perfect circle. Although these parameters detected objects in vector-expressing cells, these were attributed to spines or intrinsic dendritic tortuosity. Vector: 29.8 ± 6.2 objects/field; GluN2A WT: 59.2 ± 11.8; GluN2A(P552R): 115.4 ± 12.5. No statistical difference was observed between vector and WT; significant differences were observed between vector and mutant, and WT and mutant ($p<0.001$, 0.01, respectively; ANOVA/Tukey). Please note that we used a different pinhole in the confocal microscope (1.7) than the one used in S4 Figure (1.2) to increase the signal-to-noise ratio, which aided in the quantification procedure. Scale bar = 100 μm.
