# Supplementary material for: Molecular Mechanism of Disease-Associated Mutations in the Pre-M1 Helix of NMDA Receptors and Potential Rescue Pharmacology
Source: PLoS Genet. 2017 Jan 17;13(1):e1006536. doi: 10.1371/journal.pgen.1006536 (PMC5240934; doi:10.1371/journal.pgen.1006536)
Supplement: S6 Fig — Sliding window OE-ratio estimates (black full line), neutrality expected OE-ratio estimates derived from ExAC server. A, GluN1, B, GluN2A and C, GluN2B sliding window OE-ratio estimates (black full line), neutrality expected OE-ratio estimates derived from ExAC server accessed April, 2016 (blue full line), median OE-ratio for the gene (dark grey dashed line), 25th percentile of OE-ratio (green dashed line), 5th percentile of OE-ratio (red dashed line). The proportion of the 60,706 ExAC samples that had at least 10-fold coverage to be able to call a variant at the residue (cyan dashed line). The asterisk shows the Pro552 in GluN2A and Pro 553 in GluN2B. (PDF) [file pgen.1006536.s006.pdf]

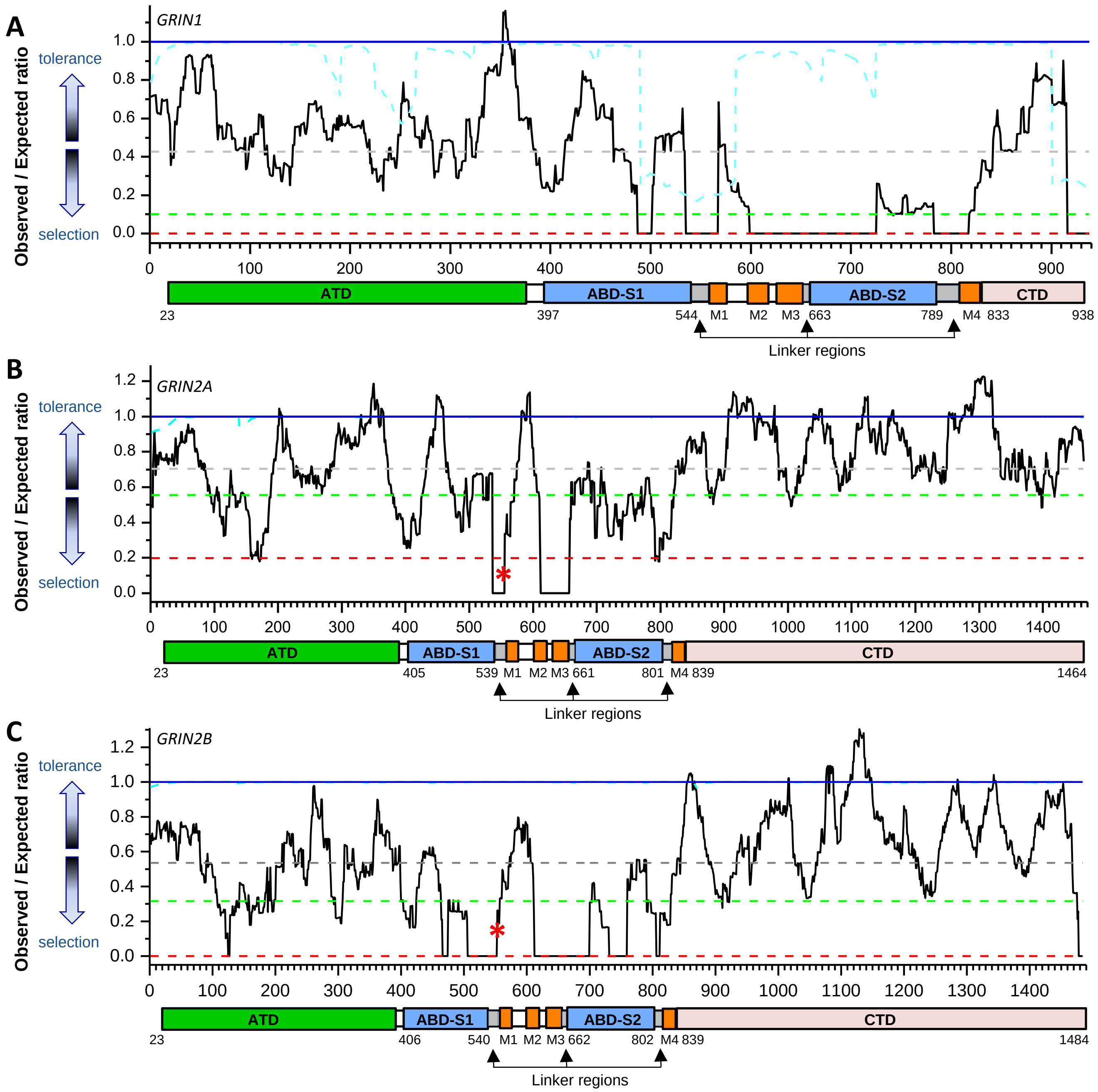

S6 Figure. OE-ratio calculated from ExAC (related to **METHODS**, **Figure-1,** and **RESULTS**). **A**, GluN1, **B**, GluN2A and **C**, GluN2B sliding window OE-ratio estimates (black full line), neutrality expected OE-ratio estimates derived from ExAC server accessed April, 2016 (blue full line), median OE-ratio for the gene (dark grey dashed line), 25th percentile of OE-ratio (green dashed line), 5th percentile of OE-ratio (red dashed line). The proportion of the 60,706 ExAC samples that had at least 10-fold coverage to be able to call a variant at the residue (cyan dashed line). The asterisk shows the Pro552 in GluN2A and Pro 553 in GluN2B.
