# Supplementary material for: Molecular Mechanism of Disease-Associated Mutations in the Pre-M1 Helix of NMDA Receptors and Potential Rescue Pharmacology
Source: PLoS Genet. 2017 Jan 17;13(1):e1006536. doi: 10.1371/journal.pgen.1006536 (PMC5240934; doi:10.1371/journal.pgen.1006536)
Supplement: S7 Fig — Simultaneous fluorescence and manual patch clamp measurements of an excitatory post-synaptic potential in a neuron expressing a QuasAr voltage indicator. Presynaptic CheRiff-expressing neurons were stimulated with a single flash of blue light at the start of the measurement. See Lou et al (2016) for current recording methods. (PDF) [file pgen.1006536.s007.pdf]

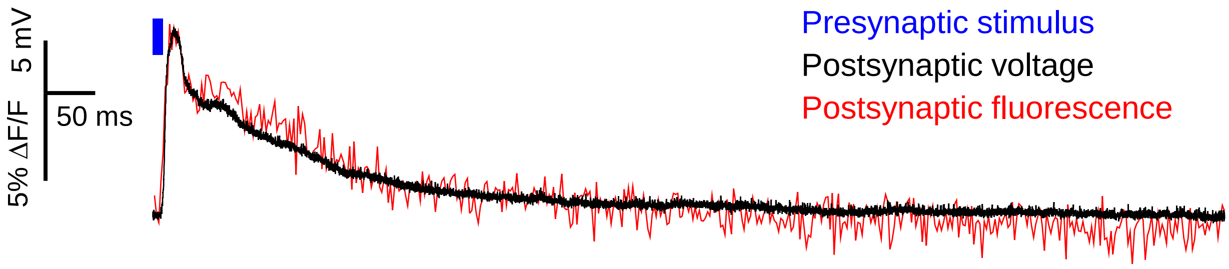

S7 Figure. Simultaneous electrical and optical voltage recording form neurons (related to **Figure-7** and **RESULTS**). Simultaneous fluorescence and manual patch clamp measurements of an excitatory post-synaptic potential in a neuron expressing a QuasAr voltage indicator. Presynaptic CheRiff-expressing neurons were stimulated with a single flash of blue light at the start of the measurement. See Lou et al (2016) for current recording methods.
