# Supplementary material for: Molecular Mechanism of Disease-Associated Mutations in the Pre-M1 Helix of NMDA Receptors and Potential Rescue Pharmacology
Source: PLoS Genet. 2017 Jan 17;13(1):e1006536. doi: 10.1371/journal.pgen.1006536 (PMC5240934; doi:10.1371/journal.pgen.1006536)
Supplement: S8 Fig — Left panel shows a ribbon structure of the GluN1/GluN2B receptors without the amino terminal domain. GluN1 is tan and GluN2B is light blue; regions with purple color reflect an OE-ratio below the 5th percentile, indicating the regions under the strongest purifying selection. Side view of agonist binding domain (ABD) in an expanded panel (right) shows a strong purifying selection (in purple) in regions of GluN1 S2 connecting with pre-M4 helix and GluN2B S1 connecting with pre-M1 helix. (PDF) [file pgen.1006536.s008.pdf]

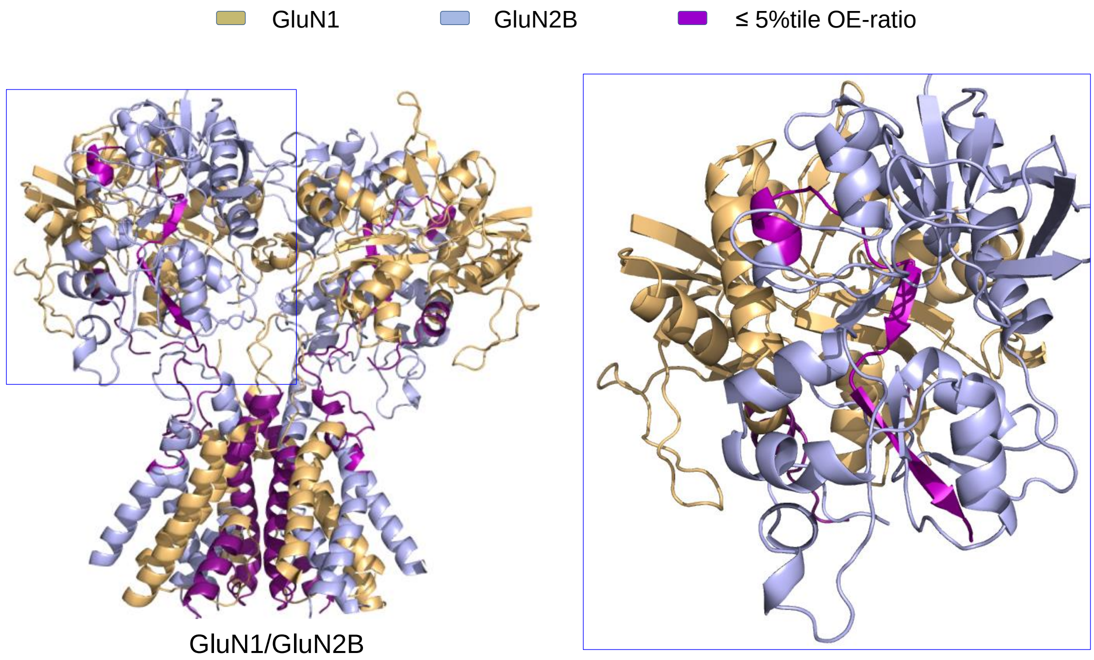

S8 Figure. Regional purifying selection of GluN1/GluN2B (related to **Figure-9** and **Discussion**). Left panel shows a ribbon structure of the GluN1/GluN2B receptors without the amino terminal domain. GluN1 is tan and GluN2B is light blue; regions with purple color reflect an OE-ratio below the 5th percentile, indicating the regions under the strongest purifying selection. Side view of agonist binding domain (ABD) in an expanded panel (*right*) shows a strong purifying selection (in purple) in regions of GluN1 S2 connecting with pre-M4 helix and GluN2B S1 connecting with pre-M1 helix.
