# Supplementary material for: Molecular Mechanism of Disease-Associated Mutations in the Pre-M1 Helix of NMDA Receptors and Potential Rescue Pharmacology
Source: PLoS Genet. 2017 Jan 17;13(1):e1006536. doi: 10.1371/journal.pgen.1006536 (PMC5240934; doi:10.1371/journal.pgen.1006536)
Supplement: S1 Table — (PDF) [file pgen.1006536.s009.pdf]

S1 Table. Patient ascertained de novo mutations (related to Table-1)

HGNC	GRCh37/hg19	HGVSc	HGVSp	Database Phenotype	PUBMED
GRIN1	chr9:140057162G>A	NM_007327.3:c.1984G>A	NP_015566.1:p.Glu662Lys	Mental retardation, autosomal dominant 8	21376300
GRIN1	chr9:140058120G>A	NM_007327.3:c.2443G>A	NP_015566.1:p.Gly815Arg	Musculoskeletal/Structural (child onset); Seizures	25356970
GRIN1	chr9:140056647C>G	NM_007327.3:c.1656C>G	NP_015566.1:p.Asp552Glu	Epileptic encephalopathy early onset with involuntary movements developmental delay & intellectual disability	25864721
GRIN1	chr9:140056647C>A	NM_007327.3:c.1656C>A	NP_015566.1:p.Asp552Glu	Epileptic encephalopathy nonsyndromic	26482601
GRIN1	chr9:140056661C>G	NM_007327.3:c.1670C>G	NP_015566.1:p.Pro557Arg	Intellectual disability	25167861
GRIN1	chr9:140057101G>A	NM_007327.3:c.1923G>A	NP_015566.1:p.Met641Ile	Epileptic encephalopathy early onset with involuntary movements developmental delay & intellectual disability	25864721
GRIN1	chr9:140057118A>C	NM_007327.3:c.1940A>C	NP_015566.1:p.Tyr647Ser	Infantile spasms	23934111
GRIN1	chr9:140057128C>G	NM_007327.3:c.1950C>G	NP_015566.1:p.Asn650Lys	Epileptic encephalopathy early onset with involuntary movements developmental delay & intellectual disability	25864721
GRIN1	chr9:140058120G>C	NM_007327.3:c.2443G>C	NP_015566.1:p.Gly815Arg	Epileptic encephalopathy early onset with involuntary movements developmental delay & intellectual disability	25864721
GRIN1	chr9:140058090C>T	NM_007327.3:c.2413C>T	NP_015566.1:p.Pro805Ser	Developmental Delay	DDD - biorxiv
GRIN1	chr9:140057361T>C	NM_007327.3:c.2077T>C	NP_015566.1:p.Phe693Leu	Developmental Delay	DDD - biorxiv
GRIN1	chr9:140057658G>A	NM_007327.3:c.2209G>A	NP_015566.1:p.Glu737Lys	Intellectual disability	27479843
GRIN2A	chr16:9943635A>G	NM_000833.4:c.1306T>C	NP_000824.1:p.Cys436Arg	Partial epilepsy atypical benign	23933819
GRIN2A	chr16:9934513C>T	NM_000833.4:c.1642G>A	NP_000824.1:p.Ala548Thr	Landau-Kleffner syndrome	23933820
GRIN2A	chr16:9928084G>C	NM_000833.4:c.1655C>G	NP_000824.1:p.Pro552Arg	Focal epilepsy with speech disorder with or without mental retardation	23033978
GRIN2A	chr16:9923442G>T	NM_000833.4:c.1845C>A	NP_000824.1:p.Asn615Lys	Focal epilepsy with speech disorder with or without mental retardation	20890276
GRIN2A	chr16:9923342G>C	NM_000833.4:c.1945C>G	NP_000824.1:p.Leu649Val	Focal epilepsy with speech disorder with or without mental retardation	23033978
GRIN2A	chr16:9923333A>C	NM_000833.4:c.1954T>G	NP_000824.1:p.Phe652Val	Focal epilepsy with speech disorder with or without mental retardation	23933820
GRIN2A	chr16:9923330T>C	NM_000833.4:c.1957A>G	NP_000824.1:p.Met653Val	Developmental Delay	DDD - biorxiv
GRIN2A	chr16:9923328C>T	NM_000833.4:c.1959G>A	NP_000824.1:p.Met653Ile	Intellectual disability	27479843
GRIN2A	chr16:9916208A>G	NM_000833.4:c.2081T>C	NP_000824.1:p.Ile694Thr	Landau-Kleffner syndrome	23933820
GRIN2A	chr16:9916194G>A	NM_000833.4:c.2095C>T	NP_000824.1:p.Pro699Ser	Benign epilepsy with centrotemporal spikes	23933819
GRIN2A	chr16:9862869G>T	NM_000833.4:c.2434C>A	NP_000824.1:p.Leu812Met	Epileptic encephalopathy	24504326
GRIN2A	chr16:9862854T>C	NM_000833.4:c.2449A>G	NP_000824.1:p.Met817Val	Global developmental delay & epilepsy	24903190
GRIN2A	chr16:9862853A>G	NM_000833.4:c.2450T>C	NP_000824.1:p.Met817Thr	Intellectual disability	27479843
GRIN2B	chr12:13769479T>C	NM_000834.3:c.1238A>G	NP_000825.2:p.Glu413Gly	Mental retardation, autosomal dominant 6	ClinVar Submission
GRIN2B	chr12:13768560C>T	NM_000834.3:c.1367G>A	NP_000825.2:p.Cys456Tyr	Mental retardation, autosomal dominant 6	23160955
GRIN2B	chr12:13768545C>A	NM_000834.3:c.1382G>T	NP_000825.2:p.Cys461Phe	Lennox-Gastaut syndrome	23934111
GRIN2B	chr12:13768132C>T	NM_000834.3:c.1570G>A	NP_000825.2:p.Asp524Asn	Intellectual disability	27479843
GRIN2B	chr12:13768083C>T	NM_000834.3:c.1619G>A	NP_000825.2:p.Arg540His	Epileptic encephalopathy, early infantile, 27	24272827
GRIN2B	chr12:13764781G>A	NM_000834.3:c.1658C>T	NP_000825.2:p.Pro553Leu	Mental retardation, autosomal dominant 6	23033978
GRIN2B	chr12:13764767C>T	NM_000834.3:c.1672G>A	NP_000825.2:p.Val558Ile	Intellectual disability	27479843
GRIN2B	chr12:13761703T>A	NM_000834.3:c.1844A>T	NP_000825.2:p.Asn615Ile	Epileptic encephalopathy, early infantile, 27	24272827
GRIN2B	chr12:13761702G>C	NM_000834.3:c.1845C>G	NP_000825.2:p.Asn615Lys	Developmental Delay	DDD - biorxiv
GRIN2B	chr12:13761694A>C	NM_000834.3:c.1853T>G	NP_000825.2:p.Val618Gly	Epileptic encephalopathy, early infantile, 27	24272827
GRIN2B	chr12:13761664G>A	NM_000834.3:c.1883C>T	NP_000825.2:p.Ser628Phe	Developmental Delay	DDD - biorxiv
GRIN2B	chr12:13761641C>G	NM_000834.3:c.1906G>C	NP_000825.2:p.Ala636Pro	Intellectual disability	23718928
GRIN2B	chr12:13761562T>G	NM_000834.3:c.1985A>C	NP_000825.2:p.Gln662Pro	Partial seizures & infantile spasms with intellectual / developmental disabilities	26544041
GRIN2B	chr12:13724865G>A	NM_000834.3:c.2044C>T	NP_000825.2:p.Arg682Cys	Mental retardation, autosomal dominant 6	20890276
GRIN2B	chr12:13724856T>G	NM_000834.3:c.2053A>C	NP_000825.2:p.Thr685Pro	Epileptic encephalopathy, early infantile, 27	ClinVar Submission
GRIN2B	chr12:13724849G>C	NM_000834.3:c.2060C>G	NP_000825.2:p.Pro687Arg	Developmental Delay	DDD - biorxiv
GRIN2B	chr12:13724844C>T	NM_000834.3:c.2065G>A	NP_000825.2:p.Gly689Ser	Developmental Delay	DDD - biorxiv
GRIN2B	chr12:13724793T>C	NM_000834.3:c.2116A>G	NP_000825.2:p.Met706Val	Intellectual disability	27479843
GRIN2B	chr12:13720138C>T	NM_000834.3:c.2419G>A	NP_000825.2:p.Glu807Lys	Developmental Delay	DDD - biorxiv
GRIN2B	chr12:13720098C>T	NM_000834.3:c.2459G>A	NP_000825.2:p.Gly820Glu	Intellectual disability	25356899
GRIN2B	chr12:13720098C>G	NM_000834.3:c.2459G>C	NP_000825.2:p.Gly820Ala	Developmental Delay	DDD - biorxiv

DDD – biorxiv: http://biorxiv.org/content/biorxiv/early/2016/04/22/049056.full.pdf

The rows highlighted in orange are the S1-M1 mutations associated with diseases
