# Supplementary material for: Molecular Mechanism of Disease-Associated Mutations in the Pre-M1 Helix of NMDA Receptors and Potential Rescue Pharmacology
Source: PLoS Genet. 2017 Jan 17;13(1):e1006536. doi: 10.1371/journal.pgen.1006536 (PMC5240934; doi:10.1371/journal.pgen.1006536)
Supplement: S2 Table — (PDF) [file pgen.1006536.s010.pdf]

S2 Table. Deactivation time course after glycine removal for GluN1-P557R and GluN2A-P552R (related to **Figure-4)**

	**Di-heteromeric Receptors**			**Tri-heteromeric Receptors**		
	**WT N1/N2A**	**N1/N2A-P552R**	**N1-P557R/N2A**	**N2A/N2A**	**N2A-P552R/N2A**	**N2A-P552R/N2A-P552R**
**Amplitude (peak, pA/pF)**	67 ± 12	18 ± 5.1#	4.5 ± 2.0#	57 ± 15	29 ± 4.3	11 ± 2.7#$
**Amplitude (SS, pA/pF)**	44 ± 8.5	---	3.9 ± 1.7*	49 ± 11	24 ± 3.4	---
**I_{SS}/I_{PEAK}%**	70 ± 4.5 %	---	88 ± 1.2 %*	88 ± 2.8 %	85 ± 4.9 %	---
**Rise time (ms)**	13 ± 0.75	874 ± 75#	8.6 ± 1.4	15 ± 1.1	14 ± 1.3	1176 ± 22#$
**τ_{FAST} (ms)**	91 ± 5.2	1093 ± 96#	415 ± 148#	127 ± 9.6	387 ± 62	1690 ± 90#$
**τ_{SLOW} (ms)**	594 ± 87	3034 ± 1263#	953 ± 99	1346 ± 242	1800 ± 254	2368 ± 304#
**%τ_{FAST}**	92 ± 1.9 %	89 ± 6.5 %	57 ± 14.5 %#	95 ± 1.5 %	81 ± 3.0 %	91 ± 3.8 %
**τ_W(ms)**	125 ± 7.6	1243 ± 84#	801 ± 103#	177 ± 9.5	621 ± 75#	1915 ± 85#$
**n**	17	12	7	8	7	17

Data are from human NMDARs. All parameters describing the time course of the macroscopic current were from responses to 1.5 sec glutamate application.

# $p < 0.05$ compared to corresponding WT receptors; one way ANOVA, Tukey post hoc

$ $p < 0.05$ compared to N2A-P552R/N2A; one way ANOVA, Tukey post hoc

* $p < 0.05$ compared to corresponding WT receptors, unpaired t-test

See **S6 Table** for F statistics.
