# Supplementary material for: Molecular Mechanism of Disease-Associated Mutations in the Pre-M1 Helix of NMDA Receptors and Potential Rescue Pharmacology
Source: PLoS Genet. 2017 Jan 17;13(1):e1006536. doi: 10.1371/journal.pgen.1006536 (PMC5240934; doi:10.1371/journal.pgen.1006536)
Supplement: S3 Table — (PDF) [file pgen.1006536.s011.pdf]

S3 Table. Sensitivity of GluN2A-P552R to endogenous negative modulators (related to **RESULTS)**

	WT N1/N2A	N1/N2A-P552R	P-value[#]
**Mg^{2+}, IC_{50}, μM (n)**	20 ± 3.8 (6)	13 ± 2.2 (8)	0.062
**Proton, $I_{pH6.8}/I_{pH7.6}$% (n)**	44 ± 1.0% (12)	47 ± 1.6% (11)	0.108
**Zn^{2+}, IC_{50}, nM (n)**	27 ± 5.7 (24)	8.9 ± 0.4 (24)	< 0.001
**%inhibition by saturating Zn^{2+}**	60 ± 2.7% (23)	91 ± 1.7% (22)	< 0.001

Data are from human NMDARs.

[#] p-values for unpaired t-tests comparing WT N1/N2A to N1/N2A-P552R
