# Supplementary material for: Molecular Mechanism of Disease-Associated Mutations in the Pre-M1 Helix of NMDA Receptors and Potential Rescue Pharmacology
Source: PLoS Genet. 2017 Jan 17;13(1):e1006536. doi: 10.1371/journal.pgen.1006536 (PMC5240934; doi:10.1371/journal.pgen.1006536)
Supplement: S4 Table — (PDF) [file pgen.1006536.s012.pdf]

## S4 Table. Statistical analysis for data in Table-3.

| | GluN1/GluN2A | | | | | GluN1/GluN2B | | | | |
|---|---|---|---|---|---|---|---|---|---|---|
| | ANOVA | | Post hoc Tukey's P-value | | | ANOVA | | Post hoc Tukey's P-value | | |
| | F statistic | P value | GluN1-D552E/N2A | GluN1-P557R/N2A | GluN1/N2A-A548T | F statistic | P value | GluN1-D552E/N2B | GluN1-P557R/N2B | GluN1/N2B-P553L |
| **Amplitude (peak)** | F (3,32) = 6.884 | 0.001 | 0.0082 | 0.0074 | 0.0028 | F (3,27) = 10.33 | 0.0001 | 0.0024 | 0.001 | 0.0009 |
| **Glutamate, $EC_{50}$** | F (3,50) = 307.8 | <0.0001 | <0.0001 | <0.0001 | <0.0001 | F (2,21) = 256.7 | <0.0001 | 0.0001 | <0.0001 | - |
| **Glycine, $EC_{50}$** | F (3,38) = 146.6 | <0.0001 | <0.0001 | <0.0001 | <0.0001 | F (2,21) = 86.34 | <0.0001 | 0.0048 | <0.0001 | - |
