# Supplementary material for: Molecular Mechanism of Disease-Associated Mutations in the Pre-M1 Helix of NMDA Receptors and Potential Rescue Pharmacology
Source: PLoS Genet. 2017 Jan 17;13(1):e1006536. doi: 10.1371/journal.pgen.1006536 (PMC5240934; doi:10.1371/journal.pgen.1006536)
Supplement: S5 Table — (PDF) [file pgen.1006536.s013.pdf]

S5 Table. Statistical analysis for data in Table-4.

	Di-heteromeric NMDA Receptors	Tri-heteromeric NMDA Receptors				
	GluN2A vs GluN2A-P552R	ANOVA		Post hoc Tukey's P-value		
	unpaired t-test p value	F statistic	P value	N2A/N2A vs N2A-P552R/N2A	N2A/N2A vs N2A-P552R/N2A-P552R	N2A/N2A-P552R vs N2A-P552R/N2A-P552R
**Glutamate, EC_{50}**	< 0.0001	F (2,77) = 136.9	<0.0001	<0.0001	<0.0001	<0.0001
**Glycine, EC_{50},**	< 0.0001	F (2,71) = 331	<0.0001	<0.0001	<0.0001	<0.0001
**Amplitude (peak, pA/pF)**	0.9289	F (2,22) = 0.011	0.9891	---	---	---
**Amplitude (SS, pA/pF)***	---	---	---	0.6242	---	---
**I_{SS}/I_{PEAK}%***	---	---	---	0.1478	---	---
**Rise time (ms)**	< 0.0001	F (2,23) = 1662	<0.0001	0.9958	<0.0001	<0.0001
**t_{FAST} (ms)**	< 0.0001	F (2,23) = 110.7	<0.0001	0.4742	<0.0001	<0.0001
**t_{SLOW} (ms)**	---	F (2,14) = 20.7	<0.0001	0.7029	0.0001	0.0002
**%t_{FAST}**	0.0547	F (2,23) = 11.12	0.0004	0.001	0.9869	0.0014
**t_W(ms)**	< 0.0001	F (2,23) = 152.7	<0.0001	0.0672	<0.0001	<0.0001
**Charge transfer pA ms/pF**	0.00061	F (2,20) = 3.707	0.0427	0.5520	<0.05	0.1938
**Mean Open Time (ms)**	---	F (2,28) = 39.18	<0.0001	0.9996	<0.0001	<0.0001
**Current Amplitude (pA)**	---	F(2,28) = 32.01	<0.0001	0.4623	<0.0001	<0.0001
**Open t1 (ms)**	---	F (2,28) = 3.816	0.0342	0.9861	0.1011	0.0424
**Open t2 (ms)**	---	F (2,28) = 33.33	<0.0001	0.9999	<0.0001	<0.0001
**Open Area 1 (%)**	---	F (2,28) = 11.21	0.0003	0.9915	0.0016	0.0007
**Open probability**	0.2184	---	---	---	---	---

*unpaired t-test
