# Supplementary material for: Molecular Mechanism of Disease-Associated Mutations in the Pre-M1 Helix of NMDA Receptors and Potential Rescue Pharmacology
Source: PLoS Genet. 2017 Jan 17;13(1):e1006536. doi: 10.1371/journal.pgen.1006536 (PMC5240934; doi:10.1371/journal.pgen.1006536)
Supplement: S6 Table — (PDF) [file pgen.1006536.s014.pdf]

S6 Table. Statistical analyses for S2 Table.

	Di-heteromeric Receptors				Tri-heteromeric Receptors				
	ANOVA		p value		ANOVA		p value		
	F statistic	P value	N1/N2A vs N1/N2A-P552R	N1/N2A vs N1-P557R/N2A	F statistic	P value	N2A/N2A vs N2A-P552R/N2A	N2A/N2A vs N2A-P552R/N2A-P552R	N2A-P552R/N2A vs N2A-P552R/N2A-P552R
**Amplitude (peak, pA/pF)**	F (2,33) = 9.869	0.0004	0.0037	0.0018	F (2,29) = 11.37	0.0002	0.0542	0.0001	0.2076
**Amplitude (SS, pA/pF)***	---	---	---	0.0073	---	---	0.0761	---	---
**I_{SS}/I_{PEAK}%***	---	---	---	0.0152	---	---	0.5758	---	---
**Rise time (ms)**	F (2,35) = 143.9	<0.0001	<0.0001	0.9975	F (2,39) = 1369	<0.0001	0.9987	<0.0001	<0.0001
**t_{FAST} (ms)**	F (2,35) = 44.7	<0.0001	<0.0001	0.0224	F (2,39) = 118.5	<0.0001	0.1479	<0.0001	<0.0001
**t_{SLOW} (ms)**	F (2,19) = 6.178	0.0086	0.0068	0.8666	F (2,22) = 8.969	0.0014	0.7856	0.0012	0.0061
**%t_{FAST}**	F (2,35) = 6.432	0.0042	0.9517	0.0044	F (2,39) = 2.988	0.062	---	---	---
**t_W(ms)**	F (2,35) = 93.03	<0.0001	<0.0001	<0.0001	F (2,39) = 147.9	<0.0001	0.0045	<0.0001	<0.0001

* unpaired t-tests
