# Supplementary material for: Molecular Mechanism of Disease-Associated Mutations in the Pre-M1 Helix of NMDA Receptors and Potential Rescue Pharmacology
Source: PLoS Genet. 2017 Jan 17;13(1):e1006536. doi: 10.1371/journal.pgen.1006536 (PMC5240934; doi:10.1371/journal.pgen.1006536)
Supplement: S7 Table — (PDF) [file pgen.1006536.s015.pdf]

S7 Table. Statistical analysis for Table-6.

	unpaired t-test p value	
	GluN1/GluN2A vs. GluN1-P557R/GluN2A	GluN1/GluN2B vs. GluN1/GluN2B-P553R
**Amplitude (peak, pA/pF)**	0.0443	0.5815
**Amplitude (SS, pA/pF)**	0.0321	---
**I_{SS}/I_{PEAK}%**	0.0006	---
**Rise time (ms)**	0.8589	< 0.0001
**t_{FAST} (ms)**	< 0.0001	< 0.0001
**t_{SLOW} (ms)**	0.4272	0.0013
**%t_{FAST}**	0.0201	0.9024
**t_{W} (ms)**	< 0.0001	< 0.0001
**Charge transfer, pA·ms/pF**	0.48208	---
