# Supplementary material for: Molecular Mechanism of Disease-Associated Mutations in the Pre-M1 Helix of NMDA Receptors and Potential Rescue Pharmacology
Source: PLoS Genet. 2017 Jan 17;13(1):e1006536. doi: 10.1371/journal.pgen.1006536 (PMC5240934; doi:10.1371/journal.pgen.1006536)
Supplement: S8 Table — (PDF) [file pgen.1006536.s016.pdf]

**S8 Table. Statistical analysis for Table-7.**

| | Q and K ANOVA | | Post hoc Tukey's P-value vs WT 2A | | G, A, I, and L ANOVA | | Post hoc Tukey's P-value vs WT 2A | | | |
|---|---|---|---|---|---|---|---|---|---|---|
| | **F statistic** | **P value** | **P552Q** | **P552K** | **F statistic** | **P value** | **P552G** | **P552A** | **P552I** | **P552L** |
| **Glutamate, $EC_{50}$** | F (6,36) = 124.8 | <0.0001 | <0.0001 | 0.9473 | <0.0001 | <0.0001 | <0.0001 | 0.9473 | <0.0001 | 0.0606 |
| **Glycine, $EC_{50}$** | F (6,79) = 83.39 | <0.0001 | <0.0001 | <0.0001 | 0.9999 | 0.0275 | <0.0001 | <0.0001 | 0.0003 | 0.3416 |
| **Amplitude (peak, pA/pF)** | F (2, 27) = 19.16 | <0.0001 | 0.0002 | <0.0001 | F (4, 58) = 1.517 | 0.2091 | --- | --- | --- | --- |
| **Amplitude (SS, pA/pF)** | --- | --- | 0.0002* | --- | F (4, 58) = 43.78 | <0.0001 | <0.0001 | <0.0001 | <0.0001 | <0.0001 |
| **$I_{SS}/I_{PEAK}$** | --- | --- | <0.0001* | --- | F (4, 58) = 43.78 | <0.0001 | <0.0001 | <0.0001 | <0.0001 | <0.0001 |
| **$t_W$ desensitization (ms)** | --- | --- | <0.0001 | --- | F (4, 59) = 29.62 | <0.0001 | <0.0001 | <0.0001 | 0.998 | <0.0001 |
| **Rise time (ms)** | F (2, 28) = 6131 | <0.0001 | 0.9786 | <0.0001 | F (4, 57) = 5.376 | 0.001 | 0.0022 | 0.9969 | 0.5998 | 0.9994 |
| **$t_{FAST}$ (ms)** | F (2, 28) = 153.2 | <0.0001 | 0.9945 | <0.0001 | F (4, 58) = 10.72 | <0.0001 | <0.0001 | 0.0806 | 0.0003 | 0.0009 |
| **$t_{SLOW}$ (ms)** | --- | --- | 0.6758* | --- | F (4, 44) = 3.181 | 0.0222 | 0.7861 | 0.9441 | 0.8359 | 0.0395 |
| **%$t_{FAST}$** | F (2, 28) = 3.667 | 0.0385 | 0.0634 | 0.999 | F (4, 59) = 1.547 | 0.2004 | --- | --- | --- | --- |
| **$t_W$(ms)** | F (2, 28) = 219.9 | <0.0001 | 0.8239 | <0.0001 | F (4, 58) = 4.52 | 0.003 | 0.079 | 0.7566 | 0.0021 | 0.213 |

*unpaired t-test
