# Supplementary material for: Molecular Mechanism of Disease-Associated Mutations in the Pre-M1 Helix of NMDA Receptors and Potential Rescue Pharmacology
Source: PLoS Genet. 2017 Jan 17;13(1):e1006536. doi: 10.1371/journal.pgen.1006536 (PMC5240934; doi:10.1371/journal.pgen.1006536)
Supplement: S9 Table — (PDF) [file pgen.1006536.s017.pdf]

S9 Table. Statistical Data for Figure-8.

A. Repeated Measures ANOVA/Bonferroni: Luciferase Assays (0.3 µg DNA/well)

	**P value**	**F statistic**	**WT GluN2A (- mem) vs. WT GluN2A (+ mem)**	**WT GluN2A (- mem) vs. GluN2A-P552R (- mem)**	**GluN2A-P552R (- mem) vs. GluN2A-P552R (+ mem)**
Viability (% of control; n=7)	< 0.0001	F (3, 6, 18) = 64.07	< 0.001	< 0.05	< 0.001

B. Repeated Measures ANOVA/Bonferroni: Luciferase Assays (0.6 µg DNA/well)

	**P value**	**F statistic**	**WT GluN2A (- mem) vs. WT GluN2A (+ mem)**	**WT GluN2A (- mem) vs. GluN2A-P552R (- mem)**	**GluN2A-P552R (- mem) vs. GluN2A-P552R (+ mem)**
Viability (% of control; n=8)	< 0.0001	F (3, 7, 21) = 23.11	< 0.01	ns	< 0.001

C. Repeated Measures ANOVA/Bonferroni: Cell Counts (0.6 µg DNA/well)

	**P value**	**F statistic**	**WT GluN2A (- mem) vs. WT GluN2A (+ mem)**	**WT GluN2A (- mem) vs. GluN2A-P552R (- mem)**	**GluN2A-P552R (- mem) vs. GluN2A-P552R (+ mem)**
Viability (% of control; n=6)	0.0079	F (3, 5, 15) = 5.77	ns	ns	< 0.01
